# Evaluating New Chemistry to Drive Molecular Discovery: Fit for Purpose?

**DOI:** 10.1002/anie.201604193

**Published:** 2016-08-30

**Authors:** Daniel J. Foley, Adam Nelson, Stephen P. Marsden

**Affiliations:** ^1^ School of ChemistryUniversity of LeedsLeedsLS2 9JTUK; ^2^ Astbury Centre for Structural Molecular BiologyUniversity of LeedsLeedsLS2 9JTUK

**Keywords:** computational tools, lead-oriented synthesis, molecular discovery, molecular properties, synthetic chemistry

## Abstract

As our understanding of the impact of specific molecular properties on applications in discovery‐based disciplines improves, the extent to which published synthetic methods meet (or do not meet) desirable criteria is ever clearer. Herein, we show how the application of simple (and in many cases freely available) computational tools can be used to develop a semiquantitative understanding of the potential of new methods to support molecular discovery. This analysis can, among other things, inform the design of improved substrate scoping studies; direct the prioritization of specific exemplar structures for synthesis; and substantiate claims of potential future applications for new methods.

## 1 Introduction

The exploration of chemical space is an enduring challenge associated with the discovery of functional small molecules. To address this challenge, synthetic methods need to enable the exploration of diverse and novel molecular property space that is relevant to the specific discovery application in question. Guidelines have been formulated to help steer chemists towards property space that is relevant to particular classes of functional small molecules, including drugs^1^ and agrochemicals.^2^ In spite of this, the synthetic toolkit that currently dominates molecular discovery is remarkably narrow,^3^ which not only contributes to our collective unsystematic and uneven exploration of chemical space,^4^ but can also drive discovery away from optimal property space!3a

Recent high‐profile articles authored by industrial scientists have challenged the academic community to develop innovative synthetic methods that align with future discovery needs.^5^, ^6^, ^7^, ^8^, ^9^, ^10^ In many cases, compounds that would provide good starting points for discovery have substantially different properties to those of final optimized^1^, ^2^ functional molecules, and specific attributes of screening compounds,^5^ fragments,^6^ building blocks,^7^ and robust reactions^8^ for drug discovery applications have been clearly described. Such articles can galvanize the academic synthetic chemistry community. In addition, major funded initiatives can facilitate the translation of synthetic approaches developed in academia: for example, the European Lead Factory has harnessed innovative synthetic methods in the construction of its distinctive compound collection.^11^

We argue, however, that in order to substantiate the potential value of new synthetic methods for discovery applications, it is necessary for researchers to demonstrate that (and contextualize how) appropriate property space may be targeted.^12^ Although computational tools for library enumeration and molecular property prediction are widely used within industry, such tools are not yet widely used by the academic synthetic chemistry community, with only limited examples to date.^13^

Herein, we illustrate some of the limitations imposed by the failure to appropriately benchmark substrate scope; we demonstrate that simple and readily available computational methods can help design thorough investigations of the scope and limitations of new synthetic methods; and we show that these analyses can help identify future applications of emerging methods to support molecular discovery.

## 2 Demonstrating the Scope of Synthetic Methods: Not All “R Groups” are Equal

The take‐up of new synthetic methods by end‐user laboratories is, in part,^12^ reliant on demonstrating that the substrate scope is relevant to discovery applications. For example, studies should ideally demonstrate that a new method is compatible with substrates bearing heterocyclic and functionalized substituents, in addition to carbocyclic and unfunctionalized variants. In many array syntheses, the presence of polar functionality in reactants or reagents correlates strongly with unsuccessful reactions, due either to intrinsic failure of the chemistry or to purification issues. Arrays of produced compounds hence tend to be systematically less polar than designed, an undesirable outcome termed “logP drift”.^5^ An unrepresentative focus on specific structural features such as particular aromatic regioisomers can also cause unanticipated issues. For example, a historic bias towards *p*‐substitution has been observed in medicinal chemistry, which tends to reduce the three‐dimensionality of the possible products.4b

**Figure 1:**
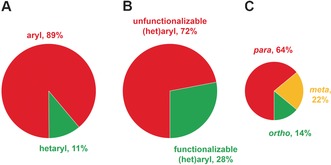
Reported variable groups in synthetic methodology papers in the first issues in 2016 of *Angew. Chem. Int. Ed*. and *J. Am. Chem. Soc*. (see the Supporting Information). A) Variation in terms of aryl and hetaryl substitution. B) The proportion of functionalizable (het)aryl versus unfunctionalizable (het)aryl groups incorporated. C) Variation of phenyl substitution. Charts are scaled according to the number of examples in the data set [504 examples for (A) and (B), 321 examples for (C)].

To illustrate the bias in the reported substrate scope of new reactions within the published literature, we analyzed the synthetic methodology papers that appeared in the first issues of *Angew. Chem. Int. Ed*. (23 papers) and *J. Am. Chem. Soc*. (6 papers) in 2016 (Figure 1). Specifically, we recorded the range of variable aryl and hetaryl groups that had been reported.

Some trends were apparent. First, the variation of (substituted) phenyl groups tends to be investigated much more thoroughly than that of hetaryl groups (Figure 1 A). Second, there are relatively few examples in which the introduced (het)aryl group bears functionalizable handles (such as halides, (protected) amines and alcohols, carbonyl and carboxyl groups, and boronic acid derivatives; Figure 1 B). Finally, the introduction of *p*‐substituted phenyl rings is demonstrated much more frequently than other substitution patterns (Figure 1 C), paralleling historic medicinal chemistry investigations.4b

The prominence of particular types of substituent may accurately describe the actual scope of the synthetic method, or may simply reflect the fact that a limited range of substrates was investigated experimentally. To distinguish between these possibilities, we suggest that best practice of researchers should be to routinely investigate a wider range of substrates, and that all unsuccessful examples should be reported (for example, in the Supporting Information). Of course, coupled with this, reviewers will need to regard the reporting of such “negative” results not necessarily as a limitation of the reported method, but rather as adding value to the reader and prospective end user, and indeed as a spur for future developments and improvements to existing methods. In the course of the above analysis, despite the undesirable bias in the variable groups reported when viewed in aggregate, there were some examples of good practice, for example, a new method for α‐(het)arylation of α‐aminomethyltrifluoroborates using dual Ir/Ni catalysis, in which a very wide range of substituted aryl and hetaryl groups was explored (25 % heteroaryl; >60 % functionalizable).^14^ More generally, we note that Glorius and Collins have described a very useful screening approach that can help identify robust reactions that are particularly tolerant of a broad range of functional groups,^15^ which is now being adopted by others.^16^

Given the apparent unsystematic exploration (or reporting) of molecular property space in many published studies, we demonstrate below how computational tools may facilitate the design of thorough analyses of the scope of new synthetic methods.

## 3 Aligning Emerging Synthetic Methods with Specific Discovery Applications

The identification of potential applications of new synthetic methods may be facilitated by computational tools for the enumeration and analysis of virtual libraries. Herein, we have used our open‐access computational tool Lead Likeness And Molecular Analysis (LLAMA);^17^ however, other open‐access tools (e.g., KNIME)^18^ and many commercial tools are also available. Such tools could also be used by synthetic chemistry researchers to select reactants that allow the full scope of their methods to be demonstrated. In most cases, we used a standard set of typical medicinal chemistry capping groups to decorate the scaffolds (see the Supporting Information). Herein, we highlight some recent exciting synthetic methods and provide evidence to suggest that they have the potential to drive the discovery of different classes of functional small molecules. For clarity, our analyses focus on specific combinations of molecular properties in each case to demonstrate the point in question, though clearly in industrial applications, the full range of relevant properties needs to be considered. Attempts to capture performance against a range of molecular property measures have been made, for example in a CNS drug‐likeness score,^19^ a metric capturing the chemical beauty of drugs,^20^ and a lead‐likeness penalty.^17^

### 3.1 Methods to Target Property Space Relevant to Discovery Applications

**Figure 2:**
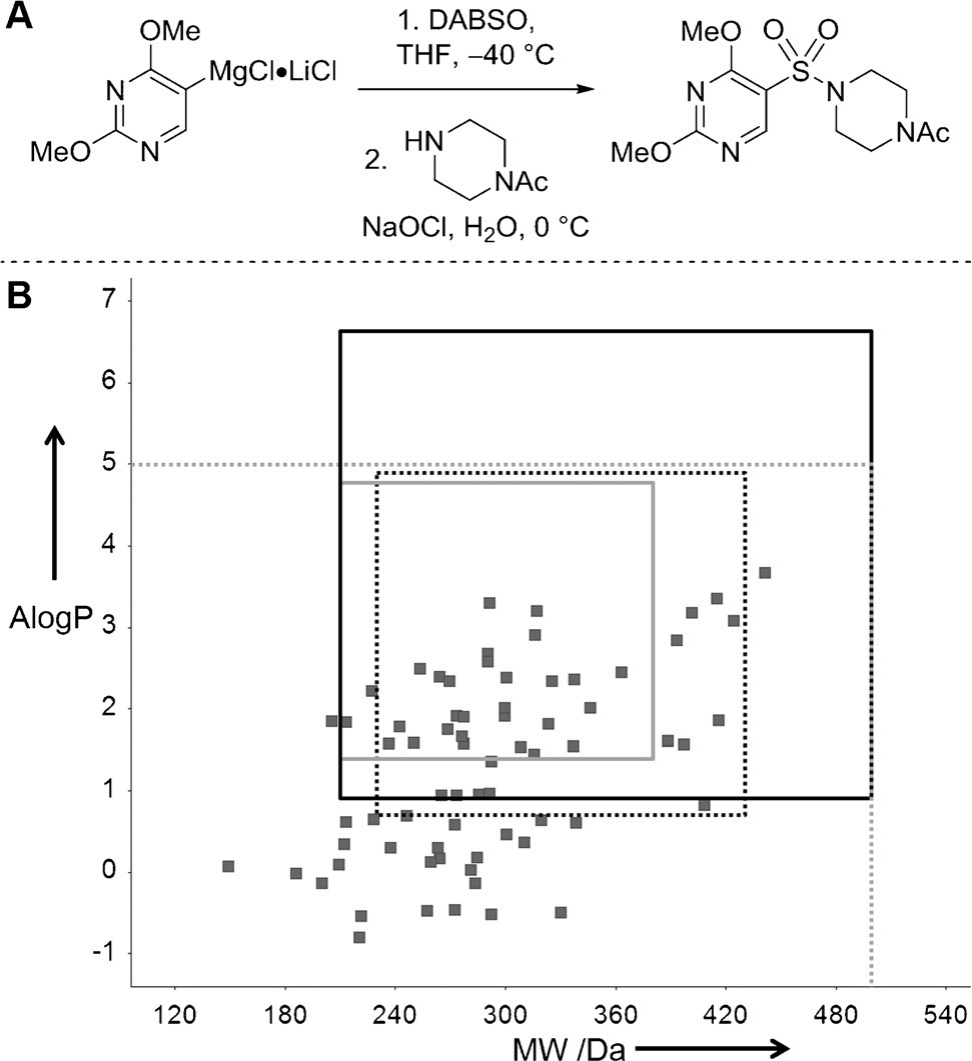
Willis’ approach to the synthesis of sulfonamides from organometallic reagents, DABSO, and amines.^21^ A) Synthesis of an exemplar sulfonamide. B) Molecular properties of the products of an array of 7 organometallic reagents and 10 amines described in the paper. The property space corresponding to Lipinski's guidelines for orally bioavailable drugs (dashed grey line), and Clarke's parameters for insecticides (black line), fungicides (solid grey line), and herbicides (dashed black line) are indicated. DABSO is the bis‐SO_2_ adduct of 1,4‐diazabicyclo[2.2.2]octane (DABCO).

As discussed in Section 2 above, it is highly desirable that any presentation of a new synthetic method examines an appropriately broad substrate set, for example in terms of chemical functionality and substitution patterns, but significant additional useful information can be garnered by examining the properties of the product set as a whole. Willis and co‐workers have developed a novel synthesis of sulfonamides in which an organometallic reagent is reacted with a sulfur dioxide equivalent (DABSO) to give a product that is then directly reacted with an aqueous solution of an amine under oxidative conditions (Figure 2 A).^21^ The method was found to be implementable in array format, and using a combination of 7 organometallic reagents and 10 amines, the synthesis of 65 (of 70 possible) products was successfully demonstrated. The diverse range of functionalized substrates used in this investigation was particularly notable.

The authors’ own molecular property analysis suggested that their method has the potential to support both drug and agrochemical discovery. Our analysis (Figure 2 B) confirms that all of the array products have molecular weights and calculated lipophilicities (AlogP) consistent with Lipinski's guidelines for orally bioavailable drugs. Moreover, a significant proportion also have molecular weights and lipophilicities that meet Clarke's guidelines2a for insecticides (60 % of the compounds; 210≤MW≤500 and 0.9≤logP≤6.6), fungicides (41 % of the compounds; 210≤MW≤380 and 1.4≤logP≤4.8), and herbicides (57 % of the compounds; 230≤MW≤430 and 0.7≤logP≤4.9). Whilst other molecular properties are also relevant in drug and agrochemical discovery, this simple analysis confirms the potential value of the method to support future discovery needs.

### 3.2 Design of Investigations to Test the Scope and Limitations of Synthetic Methods

**Figure 3:**
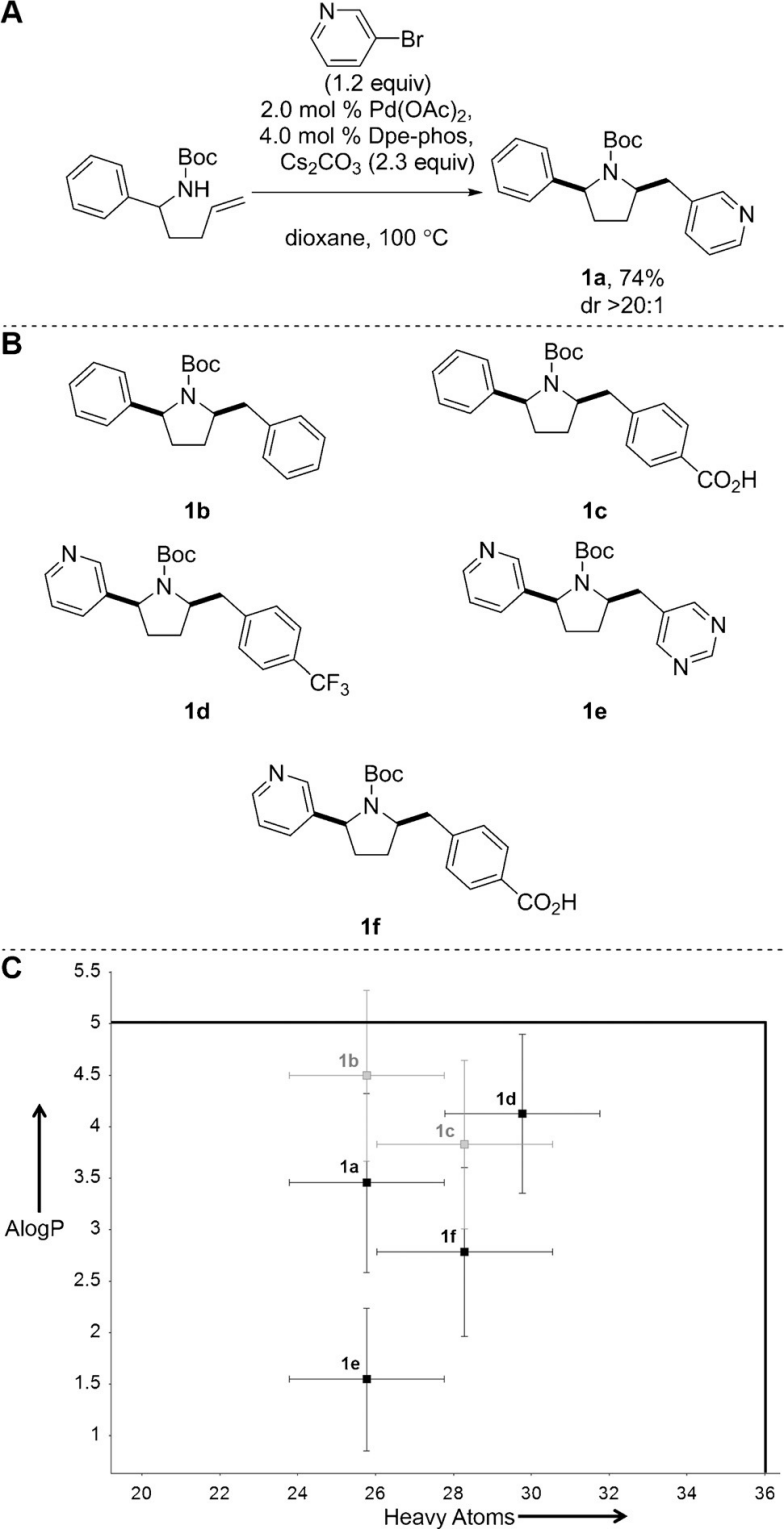
Synthesis of 2,5‐disubstituted pyrrolidines using Pd‐catalyzed aminoarylation reactions. A) Synthesis of pyrrolidine **1 a**, as reported by Wolfe.22a B) Pyrrolidines that might also be accessible using the method. C) Mean molecular properties of virtual libraries derived from the scaffolds **1**. In each case, the Boc group was removed and the scaffold was decorated once using a range of standard capping groups. Lipinski's guidelines for orally bioavailable drugs are indicated by the solid black line. Novel scaffolds (compared to a random 2 % selection of the ZINC database) are shown in black, whilst known substructures are shown in grey. Standard deviations are indicated.

Molecular property analyses could prospectively also be used to design investigations into the scope and limitations of new synthetic methods, for example by prioritizing those substrate and reagent combinations that enable the most representative examination of molecular property space. We noted the virtues of Wolfe's Pd‐catalyzed aminoarylation reactions for the synthesis of diverse pyrrolidines **1** (Figure 3 A).^22^ Distinctively, the approach enables both the synthesis of the scaffold and the introduction of a variable substituent on carbon through the aryl halide component.

We sought to demonstrate the application of aminoarylations to the synthesis of drug‐relevant scaffolds. To guide this study, we determined the molecular properties of virtual libraries derived from the previously reported scaffold (**1 a**) as well as five related scaffolds that might also be prepared (Figure 3 B): in each case, the scaffolds were decorated once with the set of exemplar capping groups. Notably, the mean calculated lipophilicity of the derived libraries could be varied enormously (ca. 3 AlogP units; Figure 3 C) within the highly structurally conserved series. We investigated the synthesis of scaffolds **1 d**–**f** experimentally, and demonstrated that all three scaffolds could be prepared in good (60–87 %) yield and with high diastereoselectivity.13c The synthetic approach was subsequently exploited in the synthesis of more than 500 compounds that were added to the compound collection of the European Lead Factory.

### 3.3 Identification of Novel Scaffolds for Drug Discovery Applications

**Figure 4:**
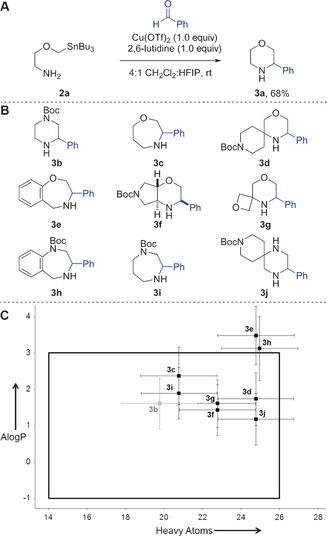
Bode's approach to nitrogen‐containing heterocycles using SnAP reagents.^23^ A) Exemplar reaction of benzaldehyde with a SnAP reagent. B) Additional scaffolds that have been, or might be, prepared from benzaldehyde using the approach. C) Mean molecular properties of virtual libraries derived from scaffolds **3** after one decoration reaction. Novel scaffolds are shown in black, whilst those that are found as substructures in a random 2 % sample from the ZINC database are shown in grey. Standard deviations are shown. Lead‐like molecular property space^5^ is indicated by the black box.

The ability to elaborate diverse, novel scaffolds for library enrichment in a synthetically efficient manner is a significant challenge. Bode and co‐workers have developed SnAP reagents that enable the synthesis of structurally diverse heterocycles from aldehydes or ketones (Figure 4).^23^ As an example, condensation of the amino‐substituted stannane **2 a** with benzaldehyde gives the corresponding morpholine **3 a** (Figure 4 A). A range of SnAP reagents (many commercially available) has been described, each leading to distinctive heterocyclic scaffolds. The potential value of the products as building blocks for drug discovery has been noted.^24^

To demonstrate potential applications of the approach, we considered a range of building blocks that might be prepared by the combination of known SnAP reagents with benzaldehyde (Figure 4 B). In each case, to complement the diversity possible by varying the aldehyde reactant, the building blocks were additionally decorated once using the exemplar capping groups (see the Supporting Information). The molecular properties of the resulting virtual libraries are shown in Figure 4 C.

We note that all of the scaffolds (**3 b**–**j**) have at least some derivatives with lead‐like molecular properties.^5^ However, with the set of capping groups used, scaffolds **3 b**, **3 c**, **3 f**, **3 g**, and **3 i** enable lead‐like property space to be targeted most effectively. Of course, the molecular properties of the derivatives may be tuned by varying the specific aldehyde and/or capping groups used. However, as others have noted,^25^ the presence of benzo‐fused rings such as those in **3 e** and **3 h** have a very significant effect on the properties of derivatives. In terms of molecular novelty, we noted that (after virtual Boc deprotection) only **3 b** is found as a substructure in a search of a random 2 % selection from the ZINC database. We conclude that the scaffolds **3 c**,**d**, **3 f**, **3 g**, and **3 i**,**j** may enable significant novel regions of lead‐like property space to be explored.

Drug discovery against central nervous system (CNS) targets raises the additional challenge of penetration of the blood–brain barrier. The challenge is exacerbated in the search for high‐quality lead molecules, which generally need to be both smaller and less lipophilic than the final drug candidates.^5^

**Figure 5:**
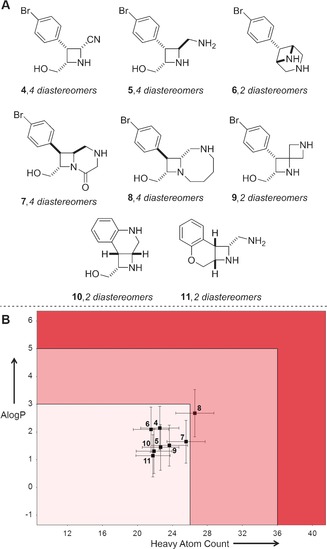
Assessment of the relevance of scaffolds to CNS drug discovery. A) The scaffolds considered in this study.^26^ B) Mean molecular properties of virtual libraries derived from the scaffolds **4**–**11** after one decoration. Standard deviations are shown. Molecular property space is shaded according to Pfizer's guidelines for relevance to CNS drug discovery (pale pink: optimal, dark pink: transitional area, red: undesirable).^19^

Marcaurelle and co‐workers have described the synthesis of a range of azetidine‐based scaffolds (**4**–**11**) that were designed to meet the needs of CNS drug discovery (Figure 5 A).^26^ We decorated each scaffold once using our standard set of medicinal chemistry capping groups. To establish relevance to CNS drug discovery, we assessed the resulting virtual libraries against established guidelines for CNS drugs (Figure 5 B).^19^ We note that 81 % of the compounds satisfied guidelines for both molecular size and lipophilicity (AlogP≤3; heavy atoms≤26), whilst the rest of the compounds fell into a transitional area (3<AlogP<5 or 26≤heavy atoms≤36). We also analyzed the molecular properties of the compounds on a per‐scaffold basis, and concluded that, using our decoration strategy, the scaffolds **4**–**6** and **9**–**11** allow CNS drug‐like space to be targeted most effectively. Marcaurelle and co‐workers have also described the pairwise decoration of **9** to yield more than 1900 compounds. The cell permeability of seven exemplar final compounds were measured experimentally, confirming their suitability for transport in the gut and at the blood–brain barrier.

### 3.4 Establishing the Shape Diversity of sp^3^‐Rich Small‐Molecule Libraries

**Figure 6:**
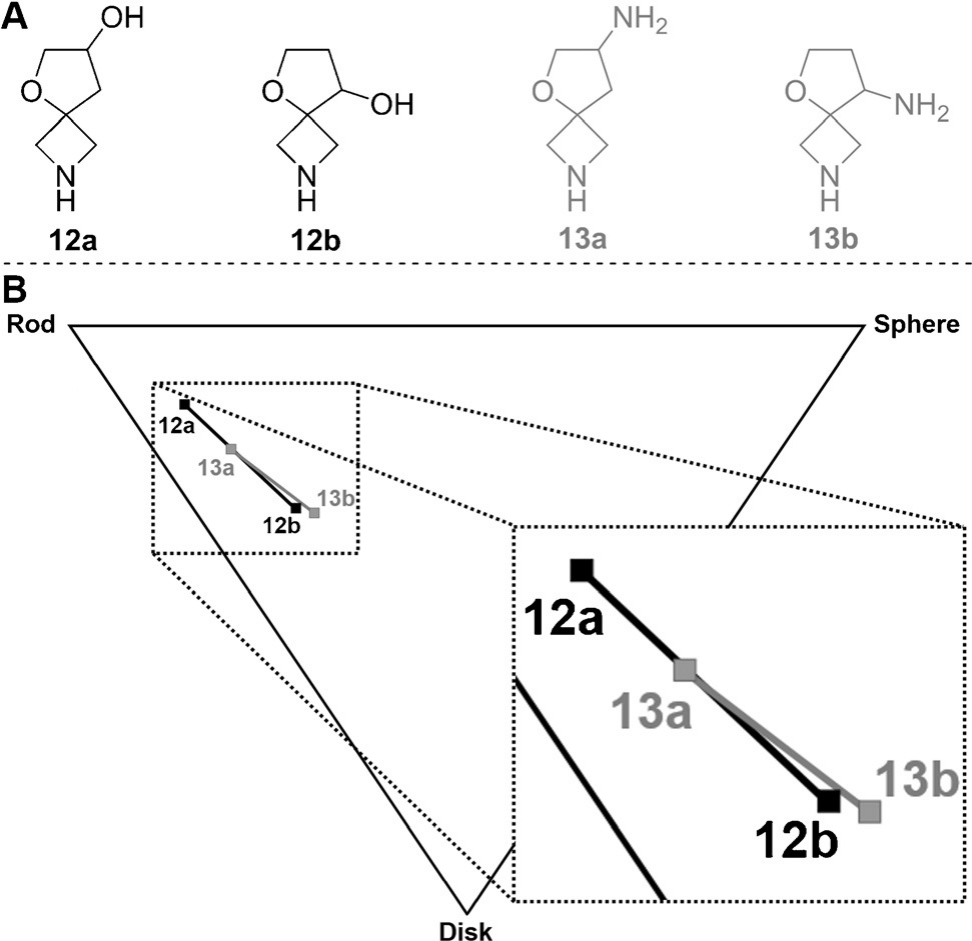
Evaluation of the shape diversity of virtual libraries based on spirocyclic scaffolds reported by Carreira and Rogers‐Evans.^27^ A) The scaffolds evaluated in this study. B) Mean principal moments of inertia of virtual libraries generated by decoration of the scaffolds once or twice using the standard set of capping groups. See the Supporting Information for further details.

Novel sp^3^‐rich molecular scaffolds that combine well‐defined molecular topologies with functional‐group handles for diversification have been noted to have significant value in drug discovery applications.^6^, ^7^, 25a However, the impact of a scaffold on the three‐dimensionality of its derivatives is often not obvious by simple inspection. Carreira and Rogers‐Evans have developed efficient syntheses of small sp^3^‐rich spirocyclic scaffolds (such as **12** and **13**; Figure 6 A).^27^ We analyzed the diversity of molecular shapes that may be explored through decoration of these scaffolds. Similar analyses could also be undertaken for other synthetic approaches to diverse molecular scaffolds.^28^

We enumerated virtual libraries in which four scaffolds **12 a**/**b** and **13 a**/**b** were decorated once or twice with the standard set of capping reagents. While there are several metrics by which shape diversity can be assessed,^29^, ^30^ we used principal moments of inertia (PMI) plots. PMIs were determined for a low‐lying conformer of each compound, and the mean PMIs for the compounds based on each scaffold are presented in Figure 6 B. We note that the shape of the resulting compounds depends critically on the position of the functionalizable groups within the scaffolds (and hence the vectors that may be explored). Compounds derived from the scaffolds **12 a** and **13 a** are systematically more linear than those derived from the regioisomeric scaffolds **12 b** and **13 b**; however, the significant difference in mean PMIs between scaffolds **12 a** and **13 a** (versus the closely aligned values for **12 b**/**13 b**) is notable and could not have been predicted by simple inspection.

**Figure 7:**
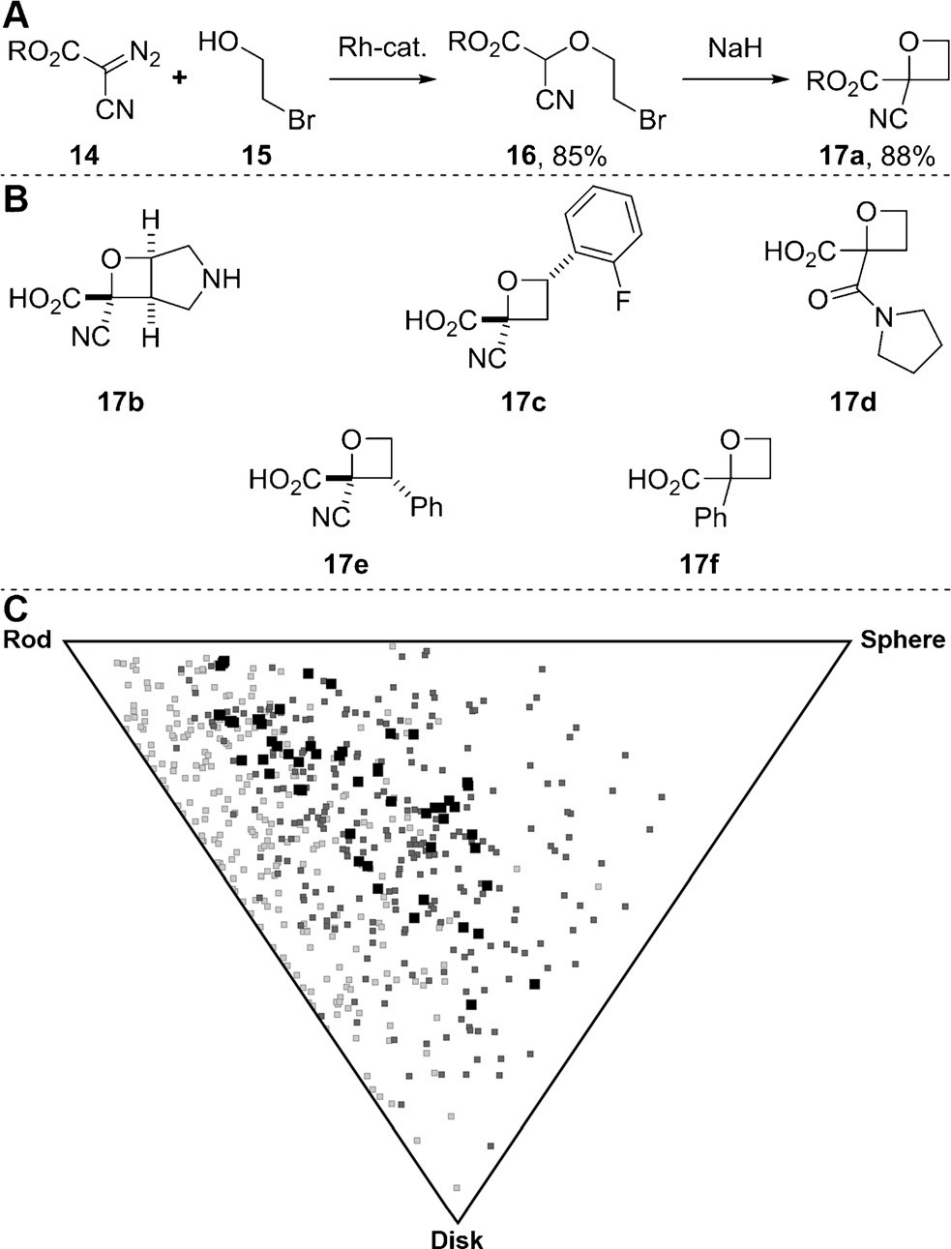
Bull's approach to substituted oxetanes.^31^ A) Synthesis of an exemplar oxetane (R=Bn, which was virtually removed before the computational study). B) Other scaffolds that were combined with 28 small amines to yield a virtual library of amides (see the Supporting Information). C) PMI plot of the 61 fragment‐like compounds found in the virtual library (black), 257 fragments randomly selected from the e‐molecules database (light gray) and 261 fragments randomly selected from the GDB‐17 database (dark gray). A plane‐of‐best‐fit analysis (Ref. 30) is also provided in the Supporting Information.

Bull and co‐workers have developed an efficient method for the synthesis of diverse substituted oxetanes, which were designed to explore three‐dimensional fragment space (Figure 7).^31^ As an example, Rh‐catalyzed reaction of the diazo compound **14** with the 2‐bromo alcohol **15** gave the corresponding O−H insertion product **16**; subsequent base‐mediated cyclization gave the substituted oxetane **17 a** (Figure 7 A).

To assess the potential value of the method to yield distinctive fragments, we enumerated a virtual library of amides by combining six deprotected scaffolds (Figure 7 B) with 28 small commercially available amines (see the Supporting Information). 36 % (61/168) of the virtual products had fragment‐like properties (9≤heavy atoms≤17; −1≤AlogP≤3). We compared the shapes of low‐lying conformers of these 61 fragments with those of 257 commercially available fragments and 261 randomly chosen fragments from the GDB‐17 database of exhaustively enumerated compounds (Figure 7 C).^32^ The GDB‐17 database provides an insight into the shape diversity of all possible fragments; this potential diversity is poorly sampled by commercially available fragments, which tend to lie close to the rod–disk edge of a PMI plot. Our analysis shows that Bull's scaffolds can be decorated to yield fragments that are significantly more three‐dimensional than commercially available fragments.

## 4 Conclusions and Future Perspectives

We have demonstrated that simple, readily available computational tools can be used to inform a semiquantitative understanding of the likely value of new chemical methods to the discovery sciences. While examples of the retrospective use of these tools are beginning to appear in the literature, we argue that it is important that they are now used to ensure that the scope and limitations of new methods are fully established. The information from such analyses can be used, among other things, to design suitably representative substrate sets against which to test reactions, or to prioritize the synthesis of “high‐value” scaffolds from a range of possible products. We argue (as have others in related contexts)^33^ that such an approach should not be regarded as a restriction, but rather as a challenge and inspiration for the academic synthetic chemistry community. A better, shared understanding of the scope and limitations of new methods will ultimately lead to more‐rapid uptake of the most valuable methods, thereby benefitting both the end‐user community (availability of trustworthy new tools) and the academic authors (higher citation, demonstrable impact).

## Biographical Information

*Daniel Foley graduated with an MChem degree from the University of Manchester in 2011, which included an industrial placement at Syngenta. He undertook a Ph.D. supervised by Steve Marsden and Adam Nelson at the University of Leeds*, *which focused on the development of synthetic approaches to lead‐like molecules (2011–2015). Daniel was awarded an EPSRC Doctoral Prize Fellowship (2015–2017) to realize the biological relevance of his novel synthetic approaches*.

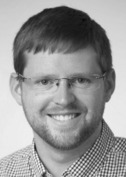

## Biographical Information

*Adam Nelson is Professor of Chemical Biology at the University of Leeds*, *and is immediate past Director of the Astbury Centre for Structural Molecular Biology. His research focuses on the development of innovative synthetic approaches and the application of synthesis to biological problems. He currently leads the review and selection of library proposals from across the EU within the European Lead Factory*.

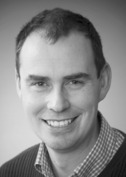

## Biographical Information

*Steve Marsden is Professor of Organic Chemistry and Head of the School of Chemistry at the University of Leeds. His research interests centre on the development of new (mainly catalytic) methods and application to the synthesis of biologically relevant motifs*, *with a special interest in quaternary hydroxy and amino acids. He recently held a Royal Society Industry Fellowship (2008–2010)*.

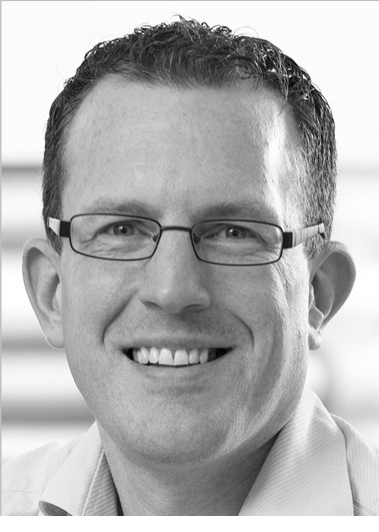

## Supporting information

As a service to our authors and readers, this journal provides supporting information supplied by the authors. Such materials are peer reviewed and may be re‐organized for online delivery, but are not copy‐edited or typeset. Technical support issues arising from supporting information (other than missing files) should be addressed to the authors.

Supplementary
