# Supplementary material for: Evaluating New Chemistry to Drive Molecular Discovery: Fit for Purpose?
Source: Angew Chem Int Ed Engl. 2016 Aug 30;55(44):13650–7. doi: 10.1002/anie.201604193 (PMC5113762; doi:10.1002/anie.201604193)
Supplement: Supplementary file 1 — Supplementary [file ANIE-55-13650-s001.pdf]

Angewandte Chemie
Eine Zeitschrift der Gesellschaft Deutscher Chemiker

Supporting Information

Evaluating New Chemistry to Drive Molecular Discovery: Fit for Purpose?

Daniel J. Foley, Adam Nelson, and Stephen P. Marsden**

anie_201604193_sm_miscellaneous_information.pdf

Supporting Information

Contents:

1.0 Data mining analysis 2

2.0 Virtual library enumeration using LLAMA 12

2.1 LLAMA Standard Capping Groups (LSCGs) 13

2.2 Analysis of Willis' methodology 14

2.3 Analysis of aminoarylation-derived scaffolds 1a-f 16

2.4 Analysis of SnAP-derived scaffolds 3b-j 19

2.5 Analysis of azetidine-based Scaffolds 4-11 23

2.6 Analysis of spirocylic scaffolds 12-13 29

2.7 Analysis of oxetane scaffolds 17a-e 34

2.7.1 Substructure filters 44

3.0 References 53

1.0 Data mining analysis

Synthetic methodologies from the first issues of *Angew. Chem. Int Ed*. (23 papers) and *J. Am. Chem. Soc*. (6 papers) in 2016 were assessed. The number of variable (het)aryl groups were considered for methodologies where (het)aryls were incorporated (and close to the site of reaction) in the key bond-forming step(s) of the methodology. The full study is shown in Table S1. The results are summarized in Table S2.

entry	Ref.	figure/ scheme/ table	no. (het)aryl examples	no. hetaryl examples	hetaryl identity	no. aryl examples	substitution patterns of phenyl substituents					Unfunctional-izable hetaryls	Functional-izable hetaryls	functionalizable hetaryl identity	Unfunctional-izable aryls	Functional-izable aryls	functionalizable aryl identity	total unfunctional-izable	total functional-izable
							unsubstituted	o-	m-	p-	complex								
1	2016ACIE218; S. Chang	Table 2, R1	5	0		5	1	0	0	4	0	0	0		2	3	p-Cl, p-Br, p-OTIPS	2	3
	DOI: 10.1002/anie.201508669	Table 3, R1	5	0		5	1	0	3	1	0	0	0		2	3	m-Cl, m-Br, m-OTIPS	2	3
		Table 4	1	0		1	0	0	0	0	1	0	0		1	0		1	0
		**SUM**	**11**	**0**		**11**	**2**	**0**	**3**	**5**	**1**	**0**	**0**		**5**	**6**		**5**	**6**
2	2016ACIE232; A. B. Smith	Table 2	3	2	2-pyridyl, 2-thiophenyl	1	1	0	0	0	0	2	0		1	0		3	0
	DOI: 10.1002/anie.201509342	**SUM**	**3**	**2**		**1**	**1**	**0**	**0**	**0**	**0**	**2**	**0**		**1**	**0**		**3**	**0**
3	2016ACIE237; Z-Q. Liu	Table 2, R	16	3	2-furyl, 3-benzofuryl, 2-thiophenyl	13	1	2	2	5	3	3	0		11	2	p-Cl, p-Br	14	2
	DOI: 10.1002/anie.201509537	Table 3, R	5	0		5	1	0	0	4	0	0	0		4	1	p-Cl	4	1
		**SUM**	**21**	**3**		**18**	**2**	**2**	**2**	**9**	**3**	**3**	**0**		**15**	**3**		**18**	**3**
4	2016ACIE254, G. A. Molander	Scheme 3	11	3	2-benzothiophenyl, coumerin-yl, 5-pyrimidyl	8	0	0	1	7	0	2	1	CN (on coumarin)	1	7	p-CN, p-(NHAc), p-CO2Me, p-CHO, m-(COMe), p-(SO2NH2), p-OH	3	8

	DOI: 10.1002/anie.201506147	Scheme 4 and 5	13	3	2-thiophenyl, 2-imidazyl, 2-pyrimidyl	10	0	0	5	5	0	2	1	4-thiophenyl-SO2NH2	4	6	m-(COCH3), p-CN, m-CN, p-(Bpin), m-OH, m-B(OH)2,	6	7
		**SUM**	**24**	**6**		**18**	**0**	**0**	**6**	**12**	**0**	**4**	**2**		**5**	**13**		**9**	**15**
5	2016ACIE264, L. Ackermann	Scheme 1	11	0		11	1	5	2	3	0	0	0		9	2	o-Cl, o-NO2	9	2
	DOI: 10.1002/anie.201507801	Scheme 2	10	0		10	0	3	1	3	3	0	0		9	1	p-Cl	9	1
		Scheme 3	7	0		7	1	3	1	0	2	0	0		5	2	[p-Br,o-Me], o-I	5	2
		**SUM**	**28**	**0**		**28**	**2**	**11**	**4**	**6**	**5**	**0**	**0**		**23**	**5**		**23**	**5**
6	2016ACIE268, A. T. Biju	Scheme 1, R	11	1	2-furyl	10	1	1	1	6	1	1	0		7	3	p-Cl, p-NO2, m-Br	8	3
	DOI: 10.1002/anie.201507802	Scheme 2, R1	7	0		7	1	1	0	5	0	0	0		5	2	p-Br, p-NO2	5	2
		Scheme 2, R2	5	0		5	1	0	0	4	0	0	0		4	1	p-Br	4	1
		**SUM**	**23**	**1**		**22**	**3**	**2**	**1**	**15**	**1**	**1**	**0**		**16**	**6**		**17**	**6**
7	2016ACIE273, J. Wang	Scheme 2, Ar1/Ar2	9	0		9	1	2	1	5	0	0	0		8	1	p-Cl	8	1
	DOI: 10.1002/anie.201509711	Scheme 3, R	5	0		5	1	0	0	3	1	0	0		4	1	p-Cl	4	1
		Scheme 4, Ar	4	0		4	0	0	1	2	1	0	0		3	1	[o-Br,p-Cl]	3	1
		**SUM**	**18**	**0**		**18**	**2**	**2**	**2**	**10**	**2**	**0**	**0**		**15**	**3**		**15**	**3**
8	2016ACIE278, T. Shishido	Table 2 Ar	9	0		9	1	0	1	4	3	0	0		9	0		9	0
	DOI:	Table 2	5	1	3-	4	1	0	0	2	1	1	0		4	0		5	0

	10.1002/anie.201507814	R/R'			thiophenyl														
		**SUM**	**14**	**1**		**13**	**2**	**0**	**1**	**6**	**4**	**1**	**0**		**13**	**0**		**14**	**0**
9	2016ACIE307, G-J. Deng	Table 1, R	22	2	2-furyl, 2-thiophenyl	20	1	2	5	10	2	2	0		10	10	p-Cl, p-Br, p-I, p-NO2, m-Cl, m-Br, m-NO2, o-Cl, o-NO2, [m-Cl,p-Cl]	12	10
	DOI: 10.1002/anie.201508076	Scheme 2, R1	5	5	indolyl	0	0	0	0	0	0	3	2	6-Cl, 6-Br	0	0		3	2
		Scheme 2, R2	11	2	2-thiophenyl, 2-indolyl	9	1	1	3	4	0	2	0		6	3	p-Br, m-Cl, o-Cl	8	3
		**SUM**	**38**	**9**		**29**	**2**	**3**	**8**	**14**	**2**	**7**	**2**		**16**	**13**		**23**	**15**
10	2016ACIE317, A. J. Grenning	Scheme 2	1	0		1	0	0	0	1	0	0	0		1	0		1	0
	DOI: 10.1002/anie.201508100	Scheme 3	3	0		3	0	0	0	1	2	0	0		2	1	p-Cl	2	1
		Scheme 6	10	0		10	0	2	2	3	3	0	0		8	2	o-Cl, p-Cl	8	2
		**SUM**	**14**	**0**		**14**	**0**	**2**	**2**	**5**	**5**	**0**	**0**		**11**	**3**		**11**	**3**
11	2016ACIE321, D. Ma	Scheme 2, indole component	21	21	indolyl	0	0	0	0	0	0	14	7	[protected OH: 5-OBn or 5-OCOtBu], 5-Br, 5-Cl, 5-CN, 6-OBn, 6-Br, 6-Cl	0	0		14	7
	DOI: 10.1002/anie.201508117	**SUM**	**21**	**21**	**indolyl**	**0**	**0**	**0**	**0**	**0**	**0**	**14**	**7**		**0**	**0**		**14**	**7**
12	2016ACIE326, J. T. Reeves	Table 4	16	2	3-pyridyl, 5-indolyl	14	1	1	3	7	2	2	0		10	4	p-Cl, p-Br, m-(COCH3), m-CO2Me	12	4
	DOI:	**SUM**	**16**	**2**	**3-**	**14**	**1**	**1**	**3**	**7**	**2**	**2**	**0**		**10**	**4**		**12**	**4**

	10.1002/anie.201508122				**pyridyl, 5-indolyl**														
13	2016ACIE331, H. Yan	Table 2, R1	11	2	2-furyl, 3-thiophenyl	9	1	1	0	5	2	2	0		8	1	p-Cl	10	1
	DOI: 10.1002/anie.201508127	Table 3, R3	4	1	3-thiophenyl	3	0	0	0	3	0	0	1	2,4,-dichlorothiophenyl	2	1	p-Cl	2	2
		**SUM**	**15**	**3**		**12**	**1**	**1**	**0**	**8**	**2**	**2**	**1**		**10**	**2**		**12**	**3**
14	2016ACIE336, N. Yoshikai	Scheme 1, R1	1	0		1	1	0	0	0	0	0	0		1	0		1	0
	DOI: 10.1002/anie.201508262	Scheme 1, R2	6	0		6	1	0	1	4	0	0	0		5	1	p-TMS	5	1
		Scheme 2, R1/R2	7	1	2-thiophenyl	6	1	1	1	3	0	1	0		5	1	p-Cl	6	1
		**SUM**	**14**	**1**		**13**	**3**	**1**	**2**	**7**	**0**	**1**	**0**		**11**	**2**		**12**	**2**
15	2016ACIE341, S. Ogoshi	Table 1, R1	6	0		6	1	0	0	4	1	0	0		5	1	p-Cl	5	1
	DOI: 10.1002/anie.201508266	Table 1, R2	13	1	2-thiophenyl	12	0	0	0	8	4	1	0		8	4	p-CO2Me, p-Br, p-(Bpin), p-CH(OEt)2	9	4
		**SUM**	**19**	**1**		**18**	**1**	**0**	**0**	**12**	**5**	**1**	**0**		**13**	**5**		**14**	**5**
16	2016ACIE345, Y. Yang	Table 1	1	0		1	0	0	0	0	1	0	0		1	0		1	0
		Scheme 2	4	0		4	0	0	0	0	4	0	0		4	0		4	0
	DOI: 10.1002/anie.201508294	Scheme 3, R2	4	0		4	1	0	1	0	2	0	0		4	0		4	0
		**SUM**	**9**	**0**		**9**	**1**	**0**	**1**	**0**	**7**	**0**	**0**		**9**	**0**		**9**	**0**
17	2016ACIE350, N.	Table 2, Ar	8	0		8	1	0	2	5	0	0	0		6	2	p-Cl, p-Br	6	2

	Jiao																		
	DOI: 10.1002/anie.201508347	Table 3, R	9	0		9	1	1	2	4	1	0	0		7	2	p-Cl, p-Br	7	2
		Table 4, Ar	7	0		7	1	0	2	4	0	0	0		5	2	p-Cl, p-Br	5	2
		**SUM**	**24**	**0**		**24**	**3**	**1**	**6**	**13**	**1**	**0**	**0**		**18**	**6**		**18**	**6**
18	2016ACIE359, N. Shibata	Table 2	8	0		8	1	1	1	4	1	0	0		6	2	p-Br, p-NO2	6	2
	DOI: 10.1002/anie.201508574	**SUM**	**8**	**0**		**8**	**1**	**1**	**1**	**4**	**1**	**0**	**0**		**6**	**2**		**6**	**2**
19	2016ACIE373, P. N. Liu	Scheme 2, R1	8	0		8	1	0	1	5	1	0	0		7	1	p-Cl	7	1
	DOI: 10.1002/anie.201508914	Scheme 2, R2/R3	9	0		9	1	0	2	6	0	0	0		7	2	p-Cl, m-Cl	7	2
		Scheme 3, R1	8	1	3-thiophenyl	7	1	0	1	5	0	1	0		6	1	p-Cl	7	1
		Scheme 3, R2/R3	9	0		9	1	0	2	6	0	0	0		7	2	p-Cl, m-Cl	7	2
		**SUM**	**34**	**1**		**33**	**4**	**0**	**6**	**22**	**1**	**1**	**0**		**27**	**6**		**28**	**6**
20	2016ACIE387, M. Beller	Table 1	2	0		2	0	0	0	0	2	0	0		2	0		2	0
	DOI: 10.1002/anie.201508575	Table 1, R'	2	0		2	1	0	0	1	0	0	0		1	1	p-Cl	1	1
		Scheme 2, R	7	0		7	0	0	0	0	7	0	0		5	2	5-Br, 5-NH2	5	2
		Scheme 4, R	1	0		1	1	0	0	0	0	0	0		1	0		1	0
		Scheme 5	1	0		1	0	0	0	0	1	0	0		1	0		1	0
		Scheme 6	1	0		1	0	0	0	0	1	0	0		1	0		1	0
		Scheme 6, R''	2	0		2	1	1	0	0	0	0	0		2	0		2	0

		**SUM**	**16**	**0**		**16**	**3**	**1**	**0**	**1**	**11**	**0**	**0**		**13**	**3**		**13**	**3**
21	2016ACIE404, P. Knochel	Scheme 3	1	0		1	0	0	1	0	0	0	0		1	0		1	0
	DOI: 10.1002/anie.201508719	Table 1	4	0		4	0	0	3	0	1	0	0		2	2	m-CN, [o-Cl,p-Cl]	2	2
		Scheme 4	1	0		1	0	0	0	0	1	0	0		0	1	[o-Ph,p-Br]	0	1
		Scheme 5	1	0		1	0	0	0	0	1	0	0		0	1	[o-Me,p-Br]	0	1
		Scheme 7	1	0		1	0	0	0	0	1	0	0		1	0		1	0
		**SUM**	**8**	**0**		**8**	**0**	**0**	**4**	**0**	**4**	**0**	**0**		**4**	**4**		**4**	**4**
22	2016ACIE413, K. Muniz	Figure 4	24	0		24	1	3	4	11	5	0	0		18	6	o-Cl, o-Br, p-Cl, p-Br, [o-F,p-Br], [o-F,m-Cl]	18	6
	DOI: 10.1002/anie.201507180	**SUM**	**24**	**0**		**24**	**1**	**3**	**4**	**11**	**5**	**0**	**0**		**18**	**6**		**18**	**6**
23	2016ACIE427, R. Wolf	Table 1	23	0		23	6	5	0	12	0	0	0		16	7	[Et,p-Br], [Me,p-Cl], [Me,p-Br], [CH2OH,p-Cl], [CH2OH,p-Br], [CH2OH,p-NO2], [CH2OH,p-CO2Me]	16	7
	DOI: 10.1002/anie.201507170	**SUM**	**23**	**0**		**23**	**6**	**5**	**0**	**12**	**0**	**0**	**0**		**16**	**7**		**16**	**7**
24	2016JACS44, M. P. Doyle	Table 1, R1	7	1	2-furyl	6	1	0	1	3	1	1	0		4	2	p-Cl, m-Cl	5	2
	DOI: 10.1021/jacs.5b10860	Table 1, R2	3	0		3	1	0	1	1	0	0	0		1	2	p-CO2Et, m-Br	1	2
		Scheme 2, R1	3	0		3	1	0	0	1	1	0	0		2	1	p-Cl	2	1
		Scheme 2, R2	2	0		2	1	0	0	1	0	0	0		1	1	p-CO2Et	1	1

		**SUM**	**15**	**1**		**14**	**4**	**0**	**2**	**6**	**2**	**1**	**0**		**8**	**6**		**9**	**6**
25	2016JACS84, B. Chattopadhyay	Table 2	20	0		20	0	4	4	8	4	0	0		14	6	p-Cl, p-CN, p-CO2Et, m-Br, o-Br, o-Bpin	14	6
	DOI: 10.1021/jacs.5b11683	Table 5	16	0		16	0	4	5	6	1	0	0		7	9	p-Cl, p-CN, (p-OH, p-OBoc, p-OBn), o-Cl, o-Br, o-Bpin, m-Br, m-Cl, m-CN	7	9
		**SUM**	**36**	**0**		**36**	**0**	**8**	**9**	**14**	**5**	**0**	**0**		**21**	**15**		**21**	**15**
26	2016JACS112, L. Deng	Table 2, Ar	7	1	2-thiophenyl	6	1	1	0	4	0	1	0		4	2	p-Cl, p-Br	5	2
	DOI: 10.1021/jacs.5b12522	**SUM**	**7**	**1**		**6**	**1**	**1**	**0**	**4**	**0**	**1**	**0**		**4**	**2**		**5**	**2**
27	2016JACS265, Y. Lan, Y. Lu	Table 3, R	1	0		1	1	0	0	0	0	0	0		1	0		1	0
	DOI: 10.1021/jacs.5b10524	Table 5, Ar	3	0		3	1	0	0	0	2	0	0		3	0		3	0
		**SUM**	**4**	**0**		**4**	**2**	**0**	**0**	**0**	**2**	**0**	**0**		**4**	**0**		**4**	**0**
28	2016JACS369, G. Dong	Table 3	7	0		7	0	0	0	0	7	0	0		5	2	[CO2Me], [Cl]	5	2
	DOI: 10.1021/jacs.5b11120	**SUM**	**7**	**0**		**7**	**0**	**0**	**0**	**0**	**7**	**0**	**0**		**5**	**2**		**5**	**2**
29	2016JACS416, J. Zhou	Scheme 2, R	4	0		4	1	0	1	1	1	0	0		2	2	m-Cl, p-Cl	2	2
	DOI: 10.1021/jacs.5b11476	Scheme 3, R1	6	1	2-thiophenyl	5	1	1	2	1	0	1	0		4	1	p-Cl	5	1

		SUM	10	1		9	2	1	3	2	1	1	0		6	3		7	3

Table S1. Data mining study (full).

entry	reference	no. (het)aryl examples	no. hetaryl examples	no. aryl examples	substitution patterns of phenyl substituents					unfunctionalizable hetaryls	functionalizable hetaryls	unfunctionalizable aryls	functionalizable aryls	total unfunctionalizable	total functionalizable
					unsubstituted	o-	m-	p-	complex						
1	2016ACIE218; S. Chang	11	0	11	2	0	3	5	1	0	0	5	6	5	6
2	2016ACIE232; A. B. Smith	3	2	1	1	0	0	0	0	2	0	1	0	3	0
3	2016ACIE237; Z-Q. Liu	21	3	18	2	2	2	9	3	3	0	15	3	18	3
4	2016ACIE254, G. A. Molander	24	6	18	0	0	6	12	0	4	2	5	13	9	15
5	2016ACIE264, L. Ackermann	28	0	28	2	11	4	6	5	0	0	23	5	23	5
6	2016ACIE268, A. T. Biju	23	1	22	3	2	1	15	1	1	0	16	6	17	6
7	2016ACIE273, J. Wang	18	0	18	2	2	2	10	2	0	0	15	3	15	3
8	2016ACIE278, T. Shishido	14	1	13	2	0	1	6	4	1	0	13	0	14	0
9	2016ACIE307, G-J. Deng	38	9	29	2	3	8	14	2	7	2	16	13	23	15
10	2016ACIE317, A. J. Grenning	14	0	14	0	2	2	5	5	0	0	11	3	11	3
11	2016ACIE321, D. Ma	21	21	0	0	0	0	0	0	14	7	0	0	14	7
12	2016ACIE326, J. T. Reeves	16	2	14	1	1	3	7	2	2	0	10	4	12	4
13	2016ACIE331, H. Yan	15	3	12	1	1	0	8	2	2	1	10	2	12	3
14	2016ACIE336, N. Yoshikai	14	1	13	3	1	2	7	0	1	0	11	2	12	2
15	2016ACIE341, S. Ogoshi	19	1	18	1	0	0	12	5	1	0	13	5	14	5
16	2016ACIE345, Y. Yang	9	0	9	1	0	1	0	7	0	0	9	0	9	0
17	2016ACIE350, N. Jiao	24	0	24	3	1	6	13	1	0	0	18	6	18	6
18	2016ACIE359, N. Shibata	8	0	8	1	1	1	4	1	0	0	6	2	6	2
19	2016ACIE373, P. N. Liu	34	1	33	4	0	6	22	1	1	0	27	6	28	6
20	2016ACIE387, M. Beller	16	0	16	3	1	0	1	11	0	0	13	3	13	3
21	2016ACIE404, P. Knochel	8	0	8	0	0	4	0	4	0	0	4	4	4	4
22	2016ACIE413, K. Muniz	24	0	24	1	3	4	11	5	0	0	18	6	18	6
23	2016ACIE427, R. Wolf	23	0	23	6	5	0	12	0	0	0	16	7	16	7
24	2016JACS44, M. P. Doyle	15	1	14	4	0	2	6	2	1	0	8	6	9	6
25	2016JACS84, B. Chattopadhyay	36	0	36	0	8	9	14	5	0	0	21	15	21	15
26	2016JACS112, L. Deng	7	1	6	1	1	0	4	0	1	0	4	2	5	2
27	2016JACS265, Y. Lan, Y. Lu	4	0	4	2	0	0	0	2	0	0	4	0	4	0
28	2016JACS369, G. Dong	7	0	7	0	0	0	0	7	0	0	5	2	5	2
29	2016JACS416, J. Zhou	10	1	9	2	1	3	2	1	1	0	6	3	7	3
**SUM**		**504**	**54**	**450**	**50**	**46**	**70**	**205**	**79**	**42**	**12**	**323**	**127**	**365**	**139**

Table S2. Data mining study (summary).

2.0 Virtual library enumeration using LLAMA

LLAMA (**L**ead **L**ikeness **A**nd **M**olecular **A**nalysis, https://llama.leeds.ac.uk) is an open-access, web based tool which may be used to decorate and analyse molecular scaffolds. Decoration is performed using a suite of pharmaceutically relevant chemical reactions and reactants to produce a virtual library of final compounds. A list of the reactants used to decorate scaffolds is given in Section 2.1 (Figure S1). Reactions that may be used in LLAMA to decorate scaffolds include: reductive amination, Suzuki coupling, Buchwald-Hartwig amination, sulfonamide formation, urea formation, alcohol alkylation, carbamate formation, secondary amide alkylation, secondary amide arylation, amide formation, alcohol arylation, urea alkylation, urea arylation (see https://llama.leeds.ac.uk for full details of these transformations). LLAMA calculates key molecular properties for the virtual compound libraries generated, such as AlogP, molecular weight, no. heavy atoms *etc*. In addition, low energy conformers are determined for each derivative.

For each study detailed in this paper the relevant SDF files were exported from LLAMA after virtual decoration of the scaffolds, Dotmatics Vortex (Vortex v2015.12.46651) was then used to prepare the charts.

2.1 LLAMA Standard Capping Groups (LSCGs)

Below are listed the 44 standard capping groups used by LLAMA to decorate scaffolds (Figure S1).

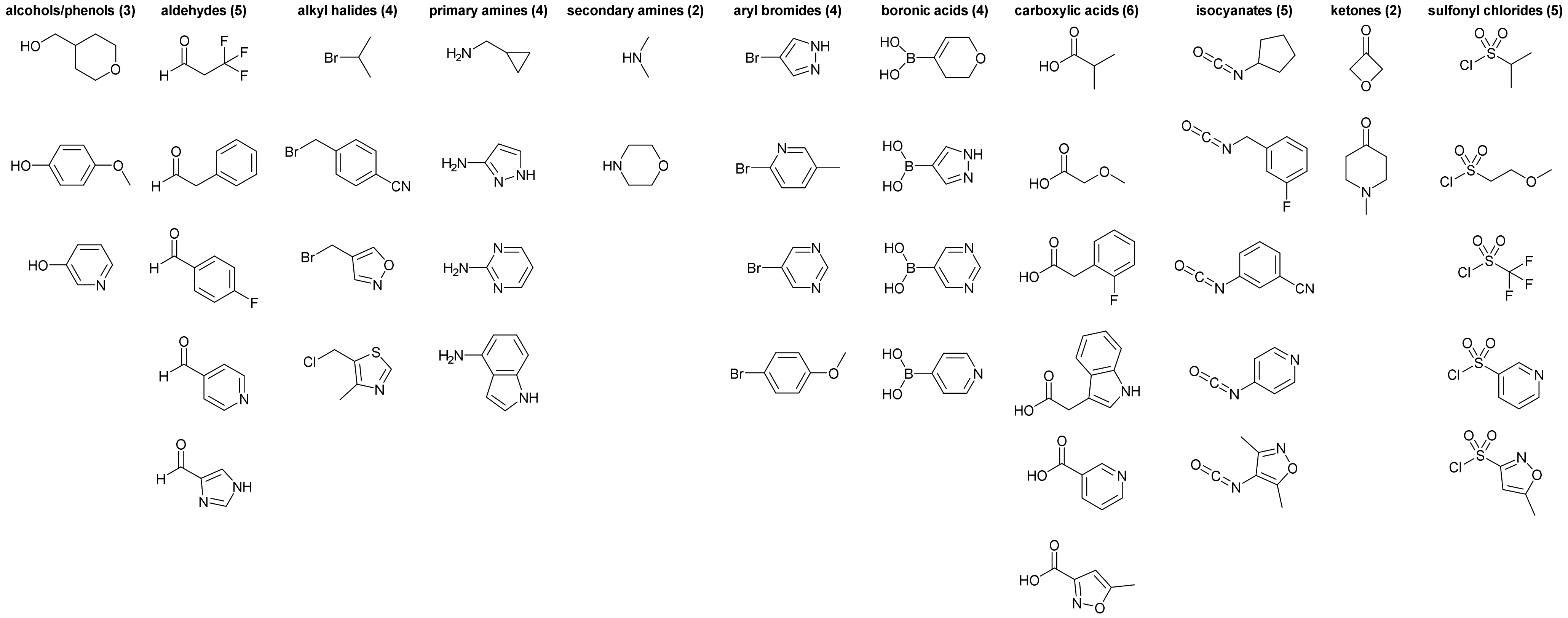

Figure S1. Standard capping reagents exploited by LLAMA for the enumeration of virtual compound libraries.

2.2 Analysis of Willis' methodology

As summarized in Figure S2, organometallic reagents (Figure S3) and amines (Figure S4) were subjected to the connective reaction to give sulfonamides. The reagents are known examples that were selected from Willis' paper.[1] The molecular properties were assessed using LLAMA (Figure 1 [Manuscript]). A PMI plot of low-lying conformers for each compound is shown in Figure S5.

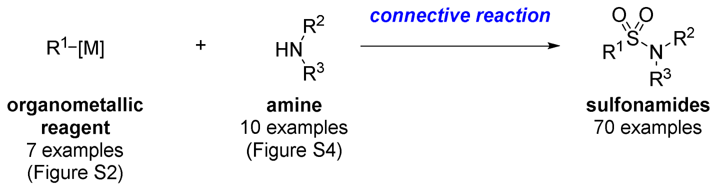

Figure S2. An overview of the chemical transformation delivered using Willis' connective reaction. [M] = Grignard/organolithium/organozinc reagent.

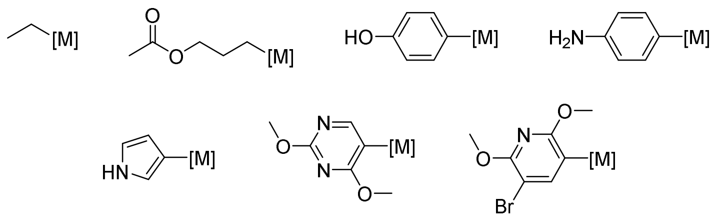

Figure S3. Organometallic reagents used in the analysis of Willis' methodology.

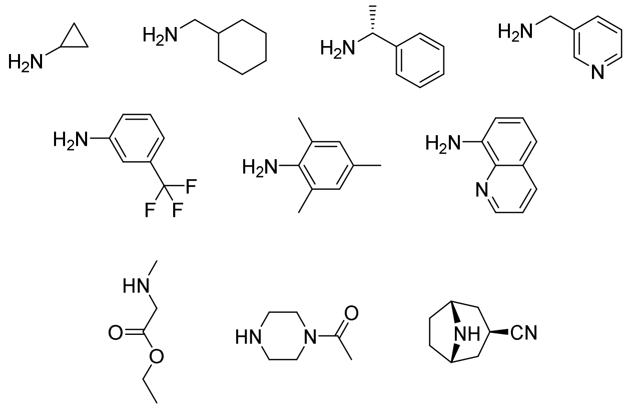

Figure S4. Amines used in the analysis of Willis' methodology.

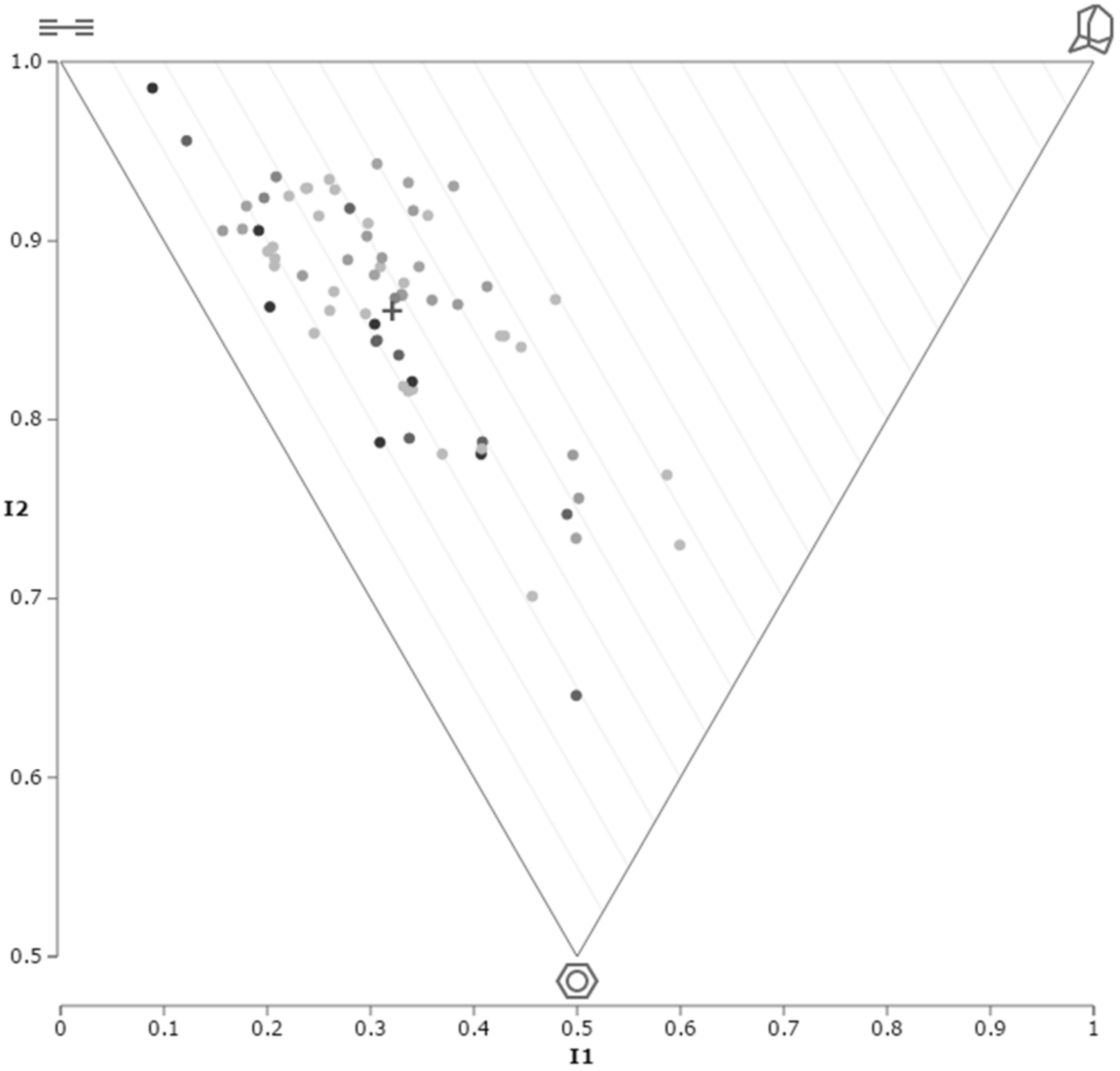

Figure S5. A PMI plot of the 70 virtual compounds generated by the process shown in Figure S2 (mean PMI coordinates: I1 = 0.321, I2 = 0.861 [denoted by **+**]).

2.3 Analysis of aminoarylation-derived scaffolds 1a-f

As summarized in Figure S6, the Boc-groups from compounds **1a-f** were virtually removed, and the resulting scaffolds **S1a-f** were decorated once using the Standard LLAMA Capping Groups (SLCGs, Figure S1). The following reaction types were enabled in LLAMA: reductive amination, Buchwald-Hartwig amination, sulfonamide formation, urea formation and amide formation. For each scaffold **S1a-f**, the average AlogP and number of heavy atoms of the derivatives were calculated (Table S3; Figure 2 [Manuscript]). Individual molecular property plots (AlogP versus number of Heavy Atoms) for compounds derived from scaffolds **S1a-f** are shown in Figure S7.

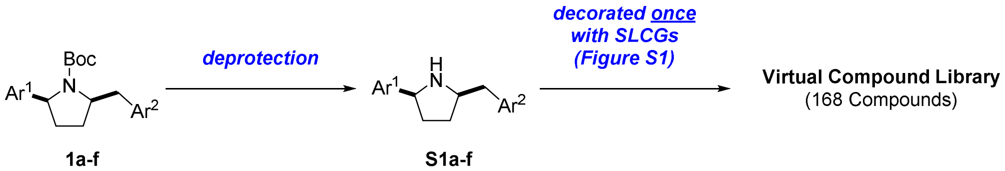

Figure S6. An overview of the decoration process for aminoarylation-derived scaffolds **1a-f**.

Scaffold	No. Derivatives	µAlogP	σAlogP	µHA	σHA
**1a**	26	3.46	0.87	25.77	1.99
**1b**	26	4.50	0.83	25.77	1.99
**1c**	32	3.83	0.82	28.28	2.26
**1d**	26	4.13	0.77	29.77	1.99
**1e**	26	1.55	0.69	25.77	1.99
**1f**	32	2.79	0.82	28.28	2.26

Table S3. Average molecular properties for the 168 derivative compounds with respect to the parent scaffold (values calculated using Dotmatics Vortex). This information was used to plot Figure 2 (manuscript).

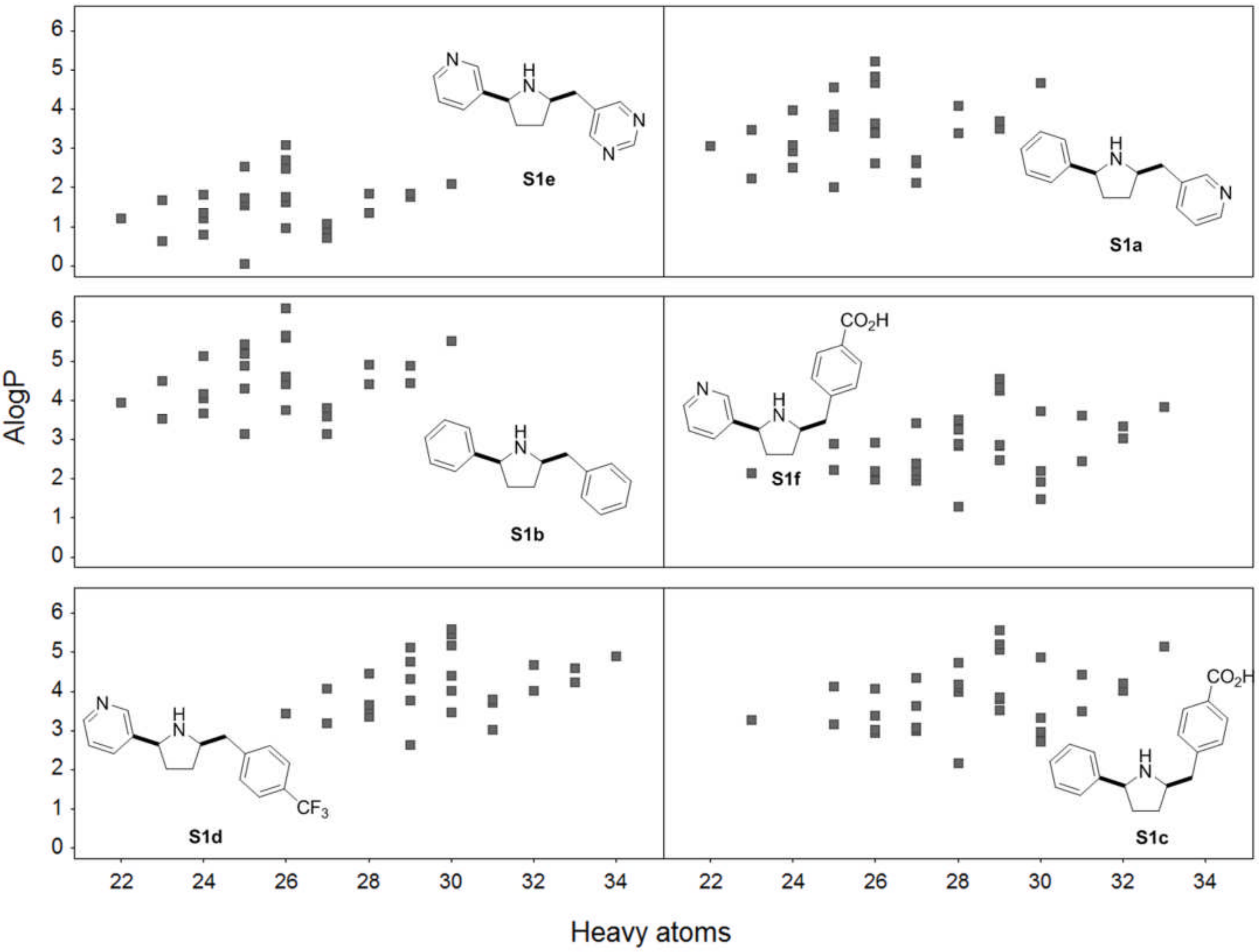

Figure S7. Molecular property analyses of the 168 derivatives (generated by the process shown in Figure S6) with respect to the parent scaffolds **S1a-f**.

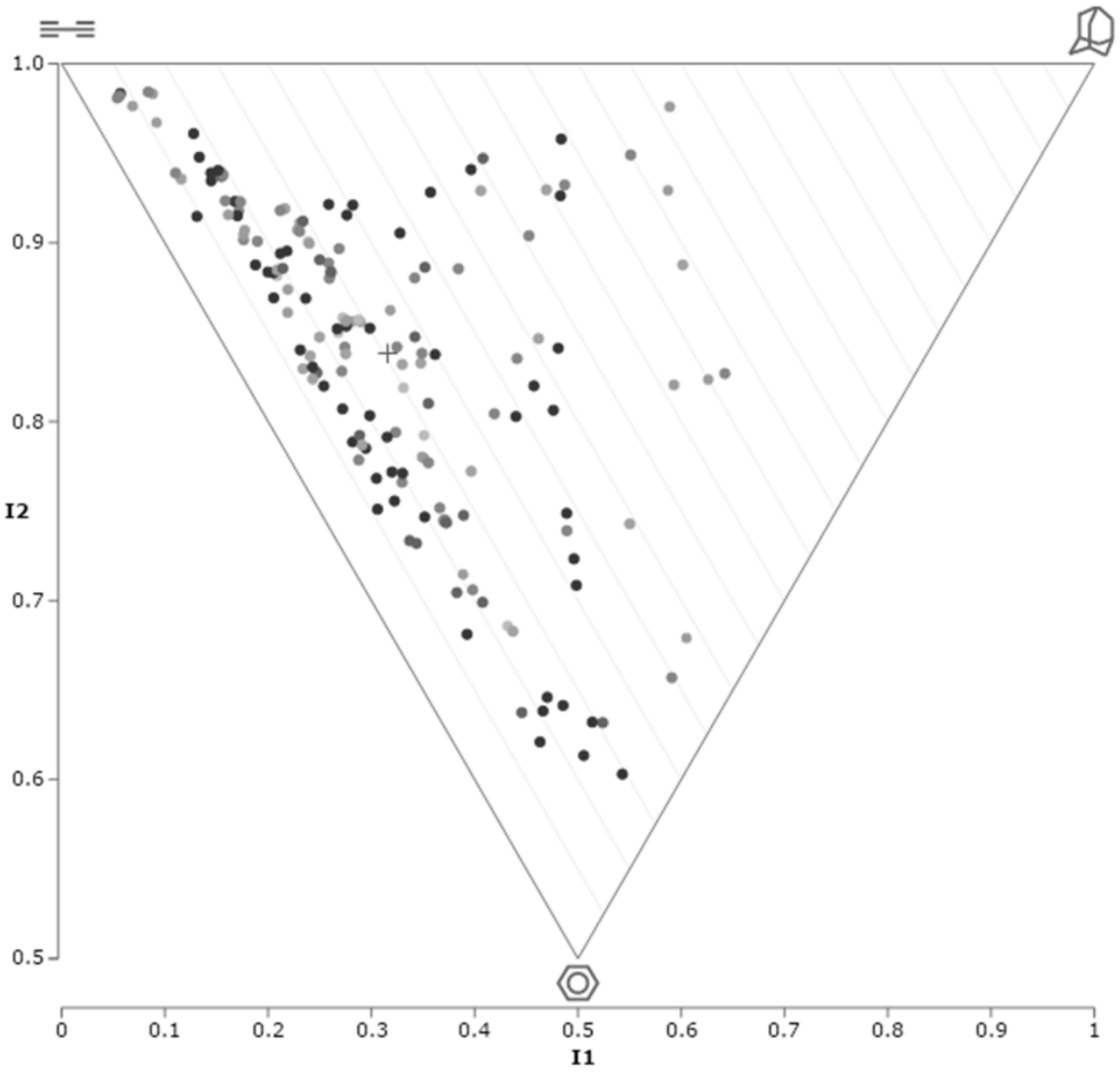

Figure S8. A PMI plot of the 168 virtual compounds generated by the process shown in Figure S6 (mean PMI coordinates: I1 = 0.316, I2 = 0.838 [denoted by **+**]).

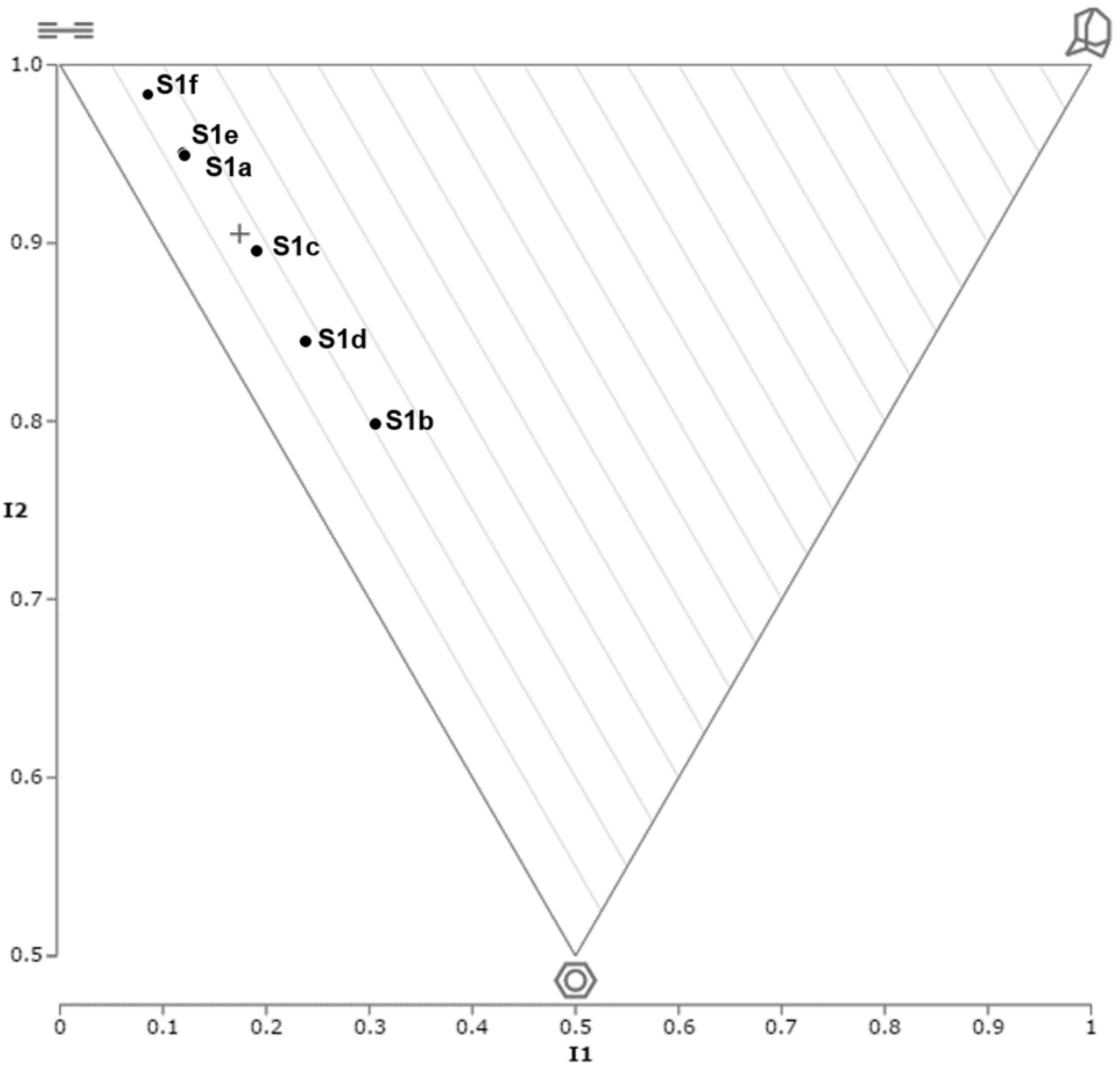

Figure S9 A PMI plot of scaffolds **S1a-f** before decoration (mean PMI coordinates: I1 = 0.174, I2 = 0.905 [denoted by **+**]).

2.4 Analysis of SnAP-derived scaffolds 3b-j

As summarized in Figure S10, any Boc-groups from compounds **3b-j** were virtually removed, and the resulting scaffolds **S3b-f** were decorated once using the Standard LLAMA Capping Groups (SLCGs, Figure S1). The following reaction types were enabled in LLAMA: reductive amination, Suzuki coupling, Buchwald-Hartwig amination, sulfonamide formation, urea formation, alcohol alkylation, carbamate formation, secondary amide alkylation, secondary amide arylation, amide formation, alcohol arylation. For each scaffold **S3b-j** the average AlogP and number of heavy atoms were calculated for the derived compounds (Table S4, Figure 3 [Manuscript]). Individual molecular property plots (AlogP versus number of Heavy Atoms) for compounds derived from scaffolds **S3b-j** are shown in Figure S11. A PMI plot of low-lying conformers for the 409 compounds derived through virtual decoration of scaffolds **S3b-j** is shown in Figure S12.

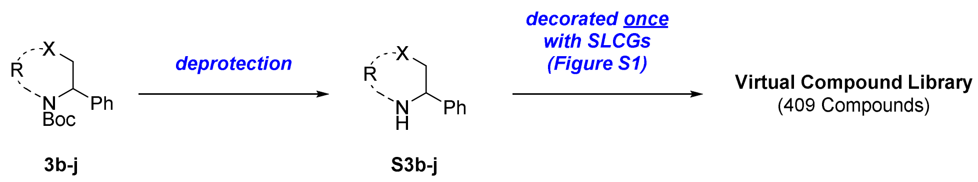

Figure S10. An overview of the decoration process for aminoarylation-derived scaffolds **3b-j**.

Scaffold	No. Derivatives	μAlogP	σAlogP	μHA	σHA
**S3b**	52	1.62	0.70	19.77	1.97
**S3c**	26	2.38	0.79	20.77	1.99
**S3d**	52	1.75	0.73	24.77	1.97
**S3e**	26	3.49	0.80	24.77	1.99
**S3f**	52	1.44	0.69	22.77	1.97
**S3g**	26	1.62	0.65	22.77	1.99
**S3h**	45	3.13	0.88	24.96	1.99
**S3i**	52	1.90	0.71	20.77	1.97
**S3j**	78	1.19	0.71	24.77	1.96

Table S4. Average molecular properties for the 409 derivative compounds generated in Figure S10 with respect to the parent scaffold (values calculated using Dotmatics Vortex).

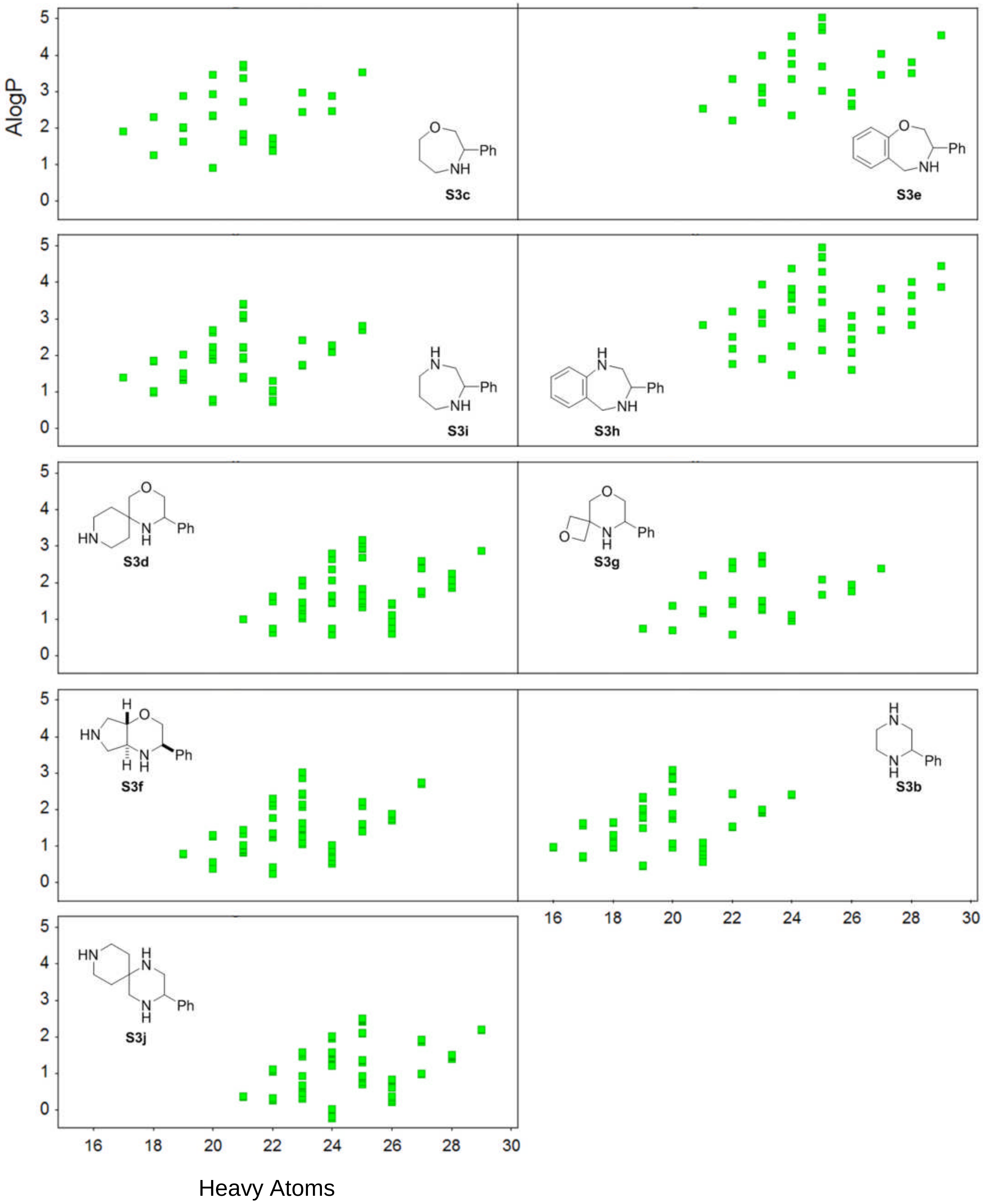

Figure S11. Molecular property analyses of the 409 virtual compounds derived through decoration of scaffolds **S3b-j** (Figure S10).

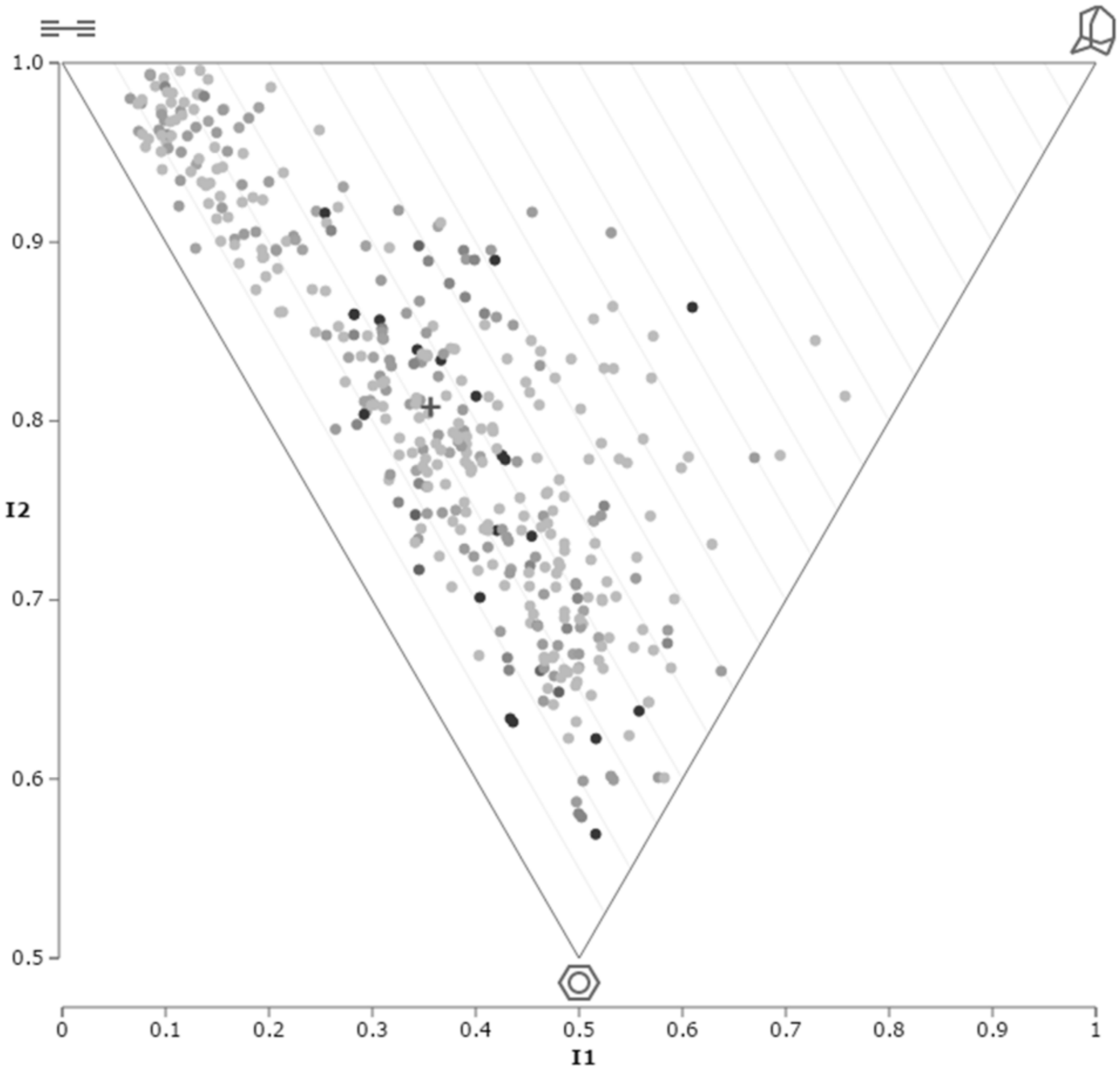

Figure S12. A PMI plot of the 409 virtual compounds generated by the process shown in Figure S10 (mean PMI coordinates: I1 = 0.356, I2 = 0.808 [denoted by **+**]).

2.5 Analysis of azetidine-based Scaffolds 4-11

As summarized in Figure S13, scaffolds **4-11** (Figure S14) were decorated once using the Standard LLAMA Capping Groups (SLCGs, Figure S1). The following reaction types were enabled in LLAMA: reductive amination, Suzuki coupling, Buchwald-Hartwig amination, sulfonamide formation, urea formation, alcohol alkylation, carbamate formation, secondary amide alkylation, secondary amide arylation, amide formation, alcohol arylation. For each scaffold **4-11** the average AlogP and number of heavy atoms were calculated for the derived compounds (Table S5, Figure 4 [Manuscript]). Individual molecular property plots (AlogP versus number of Heavy Atoms) for compounds derived from scaffolds **4-11** are shown in Figure S15. A PMI plot of low-lying conformers for the 1210 compounds derived through virtual decoration of scaffolds **4-11** is shown in Figure S16, whilst individual plots for each scaffold are shown in Figure S17.

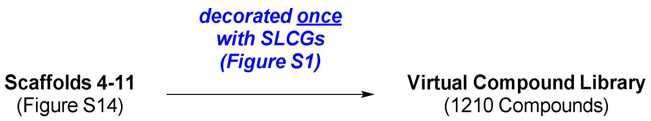

Figure S13. An overview of the decoration process for the azetidine-based scaffolds **4-11**.

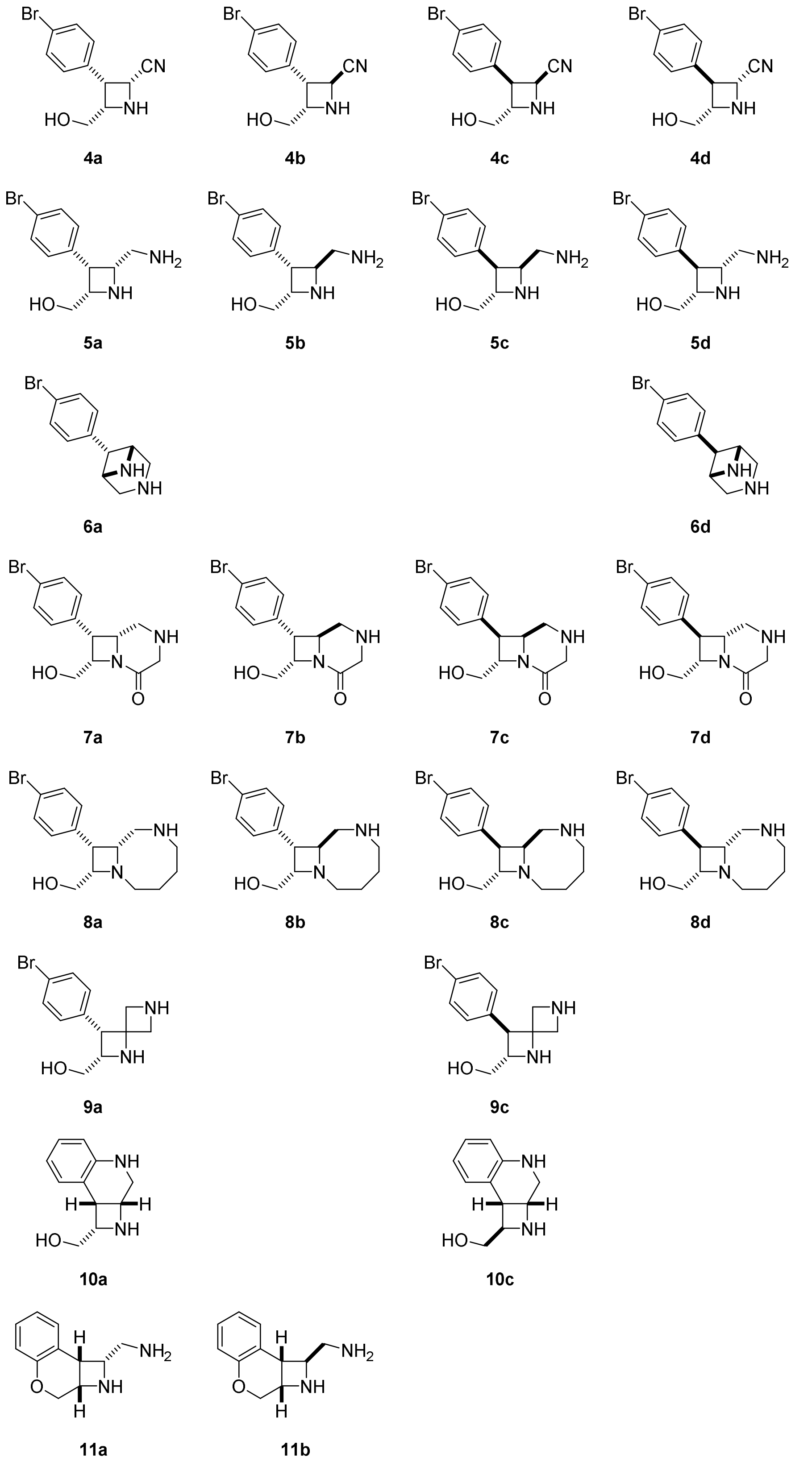

Figure S14. Azetidine-based scaffolds **4-11** included in the decoration process.

Scaffolds	No. Derivatives	μAlogP	σAlogP	μHA	σHA
**4a-d**	160	2.14	0.79	22.55	2.19
**5a-d**	264	1.45	0.83	22.64	2.10
**6a,d**	114	2.09	0.81	21.54	2.03
**7a-d**	160	1.65	0.77	25.55	2.19
**8a-d**	160	2.68	0.85	26.55	2.19
**9a,c**	132	1.51	0.74	23.64	2.10
**10a,c**	116	1.31	0.80	21.90	2.05
**11a-b**	104	1.15	0.76	21.77	1.96

Table S5. Average molecular properties for the 1210 derivative compounds generated by the process shown in Figure S13 with respect to parent scaffolds **4-11** (values calculated using Dotmatics Vortex).

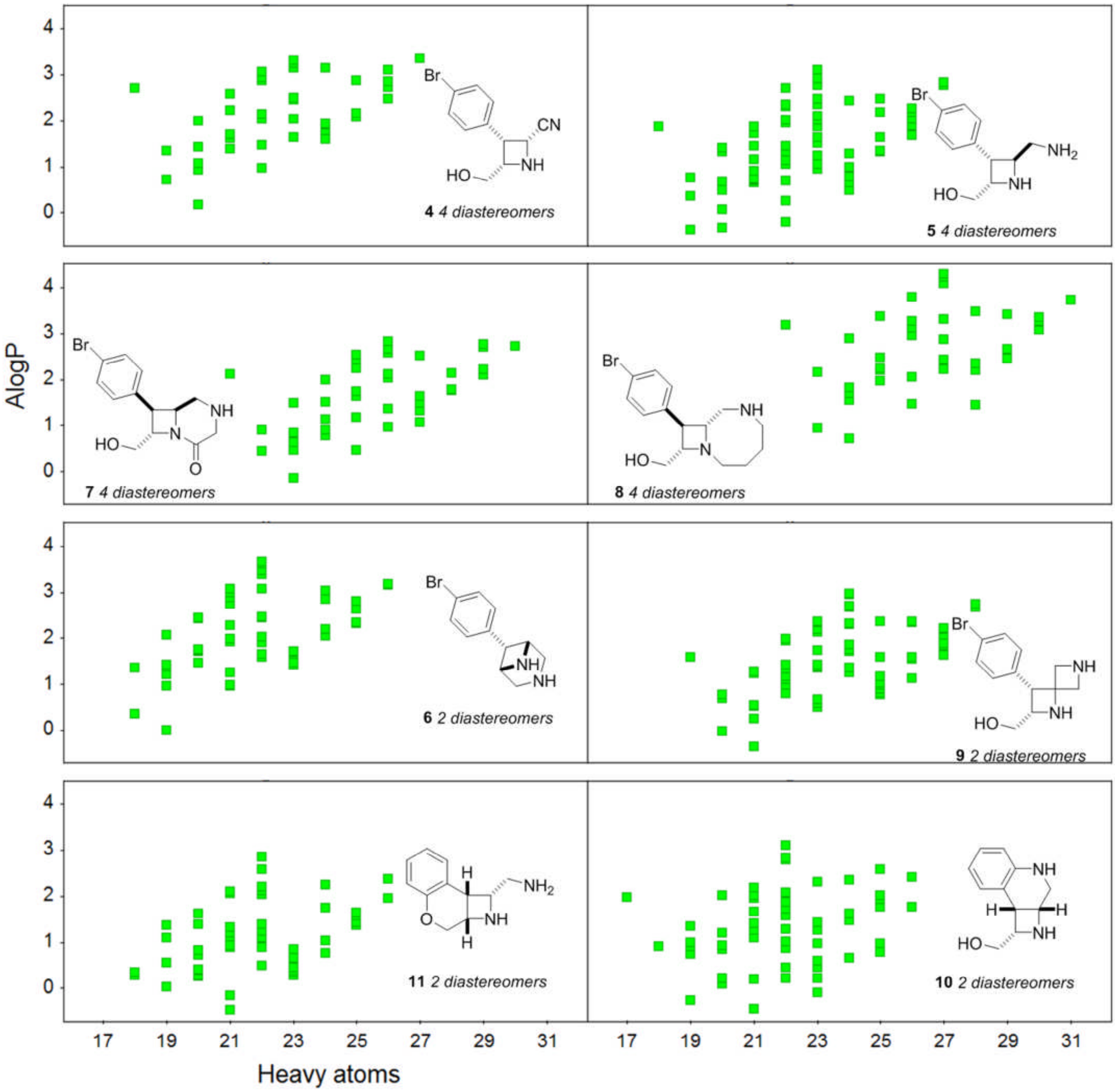

Figure S15. Molecular property analyses of the 1210 virtual compounds derived through decoration of scaffolds **4-11** (Figure S14).

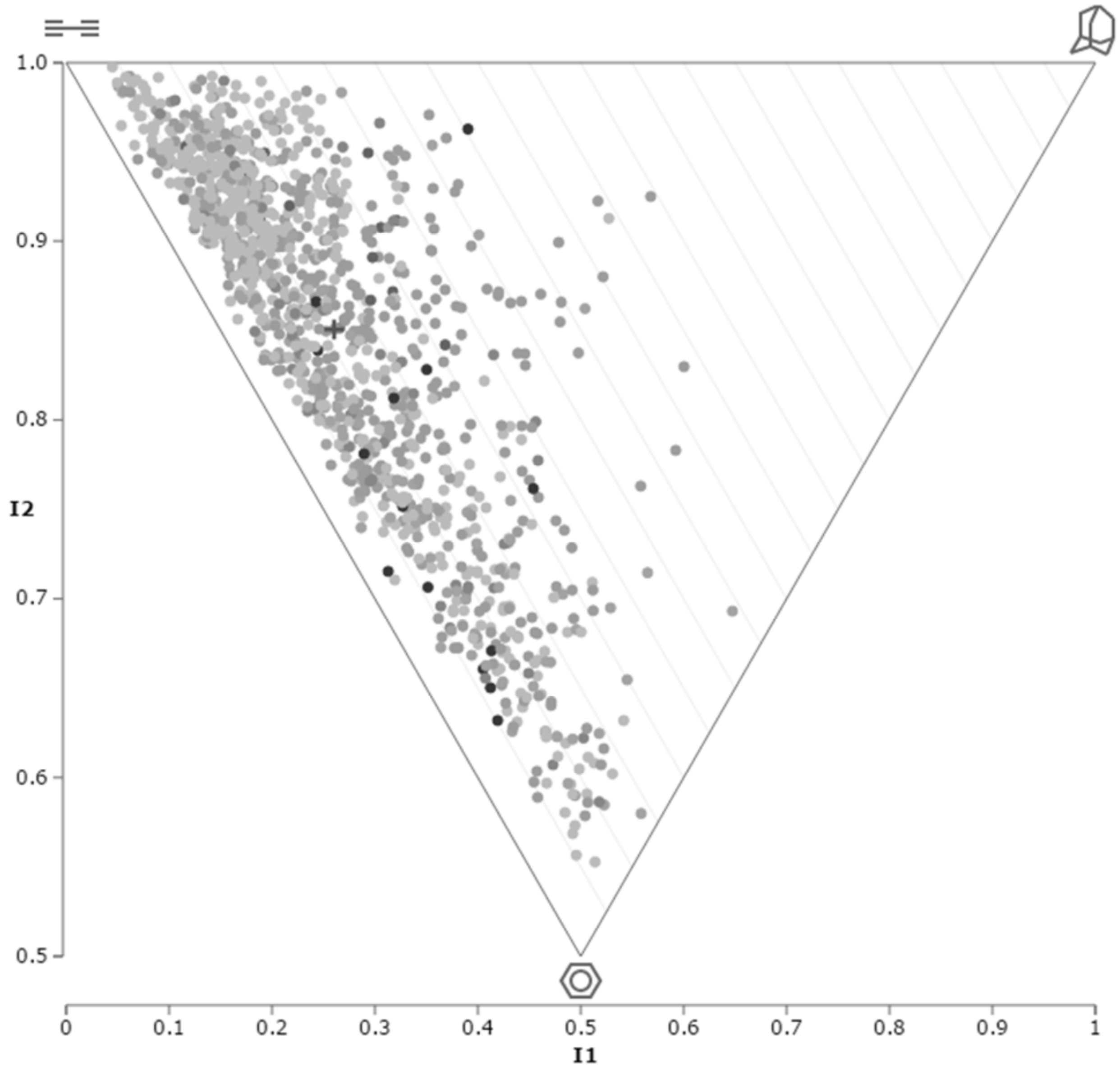

Figure S16. A PMI plot of the 1210 virtual compounds generated by the process shown in Figure S13 (mean PMI coordinates: I1 = 0.260, I2 = 0.850 [denoted by **+**]).

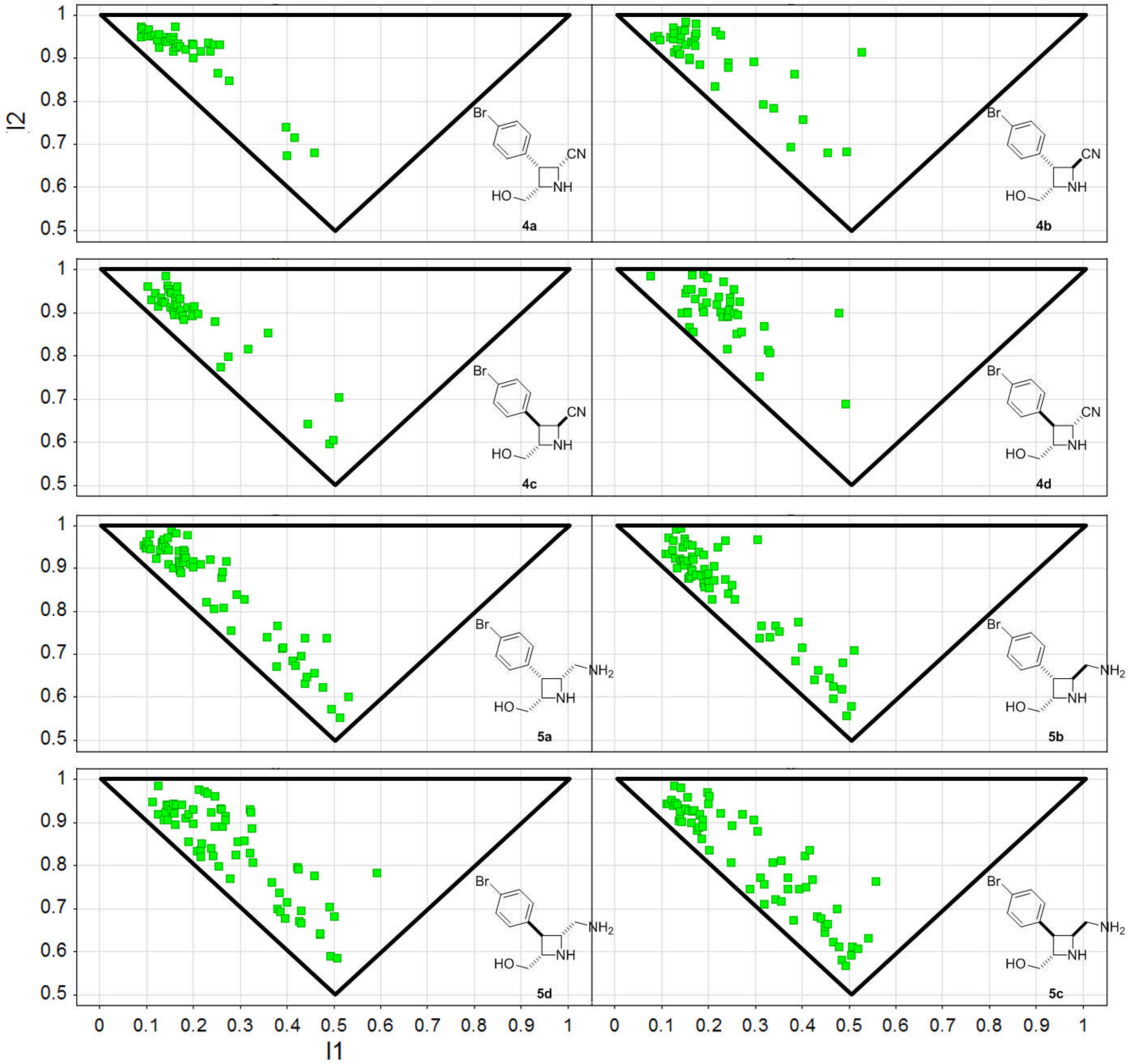
I2
I1
4a
4b
4c
4d
5a
5b
5d
5c
Br
CN
NH
HO
NH2

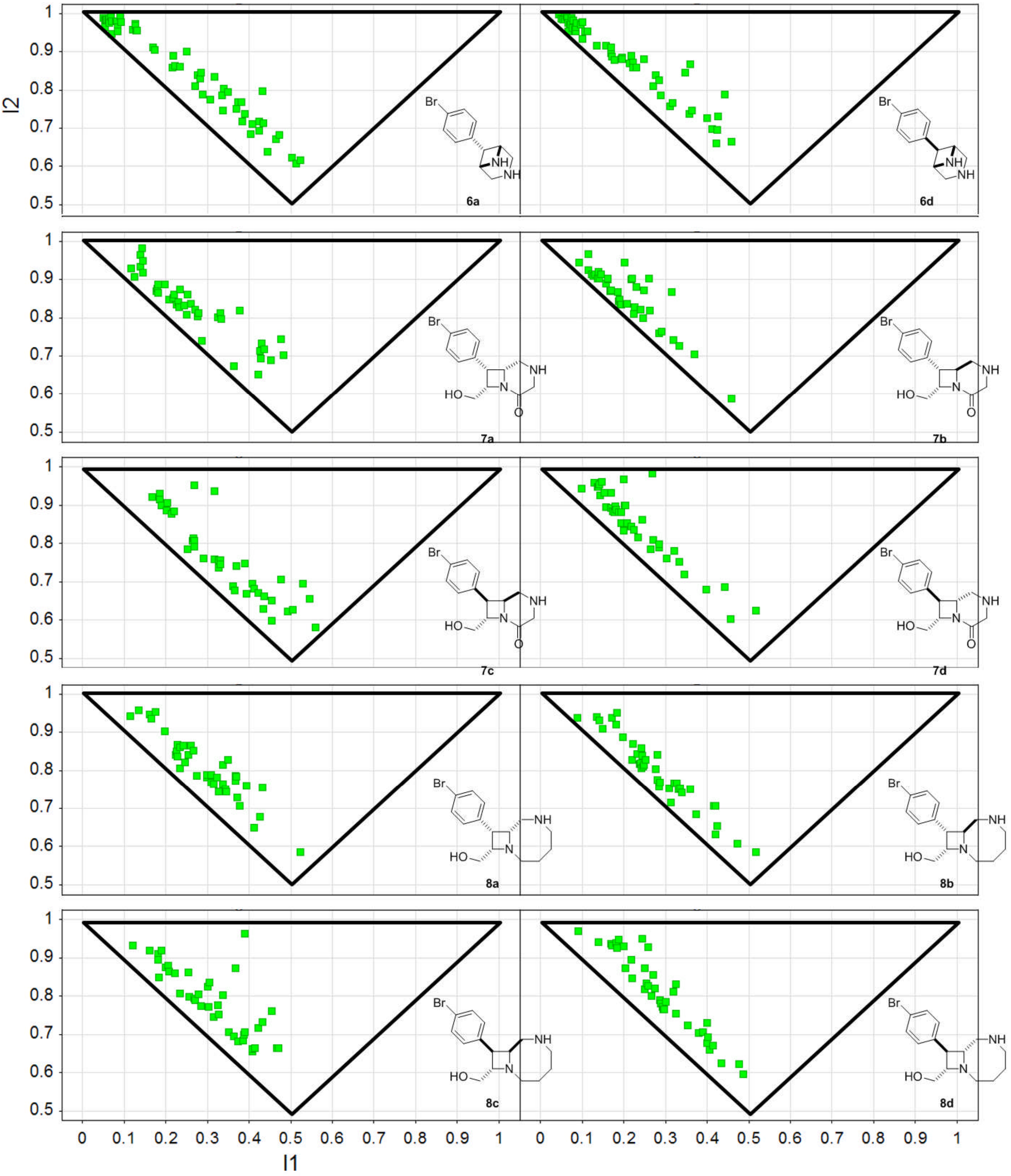

I2
I1
6a
6d
7a
7b
7c
7d
8a
8b
8c
8d

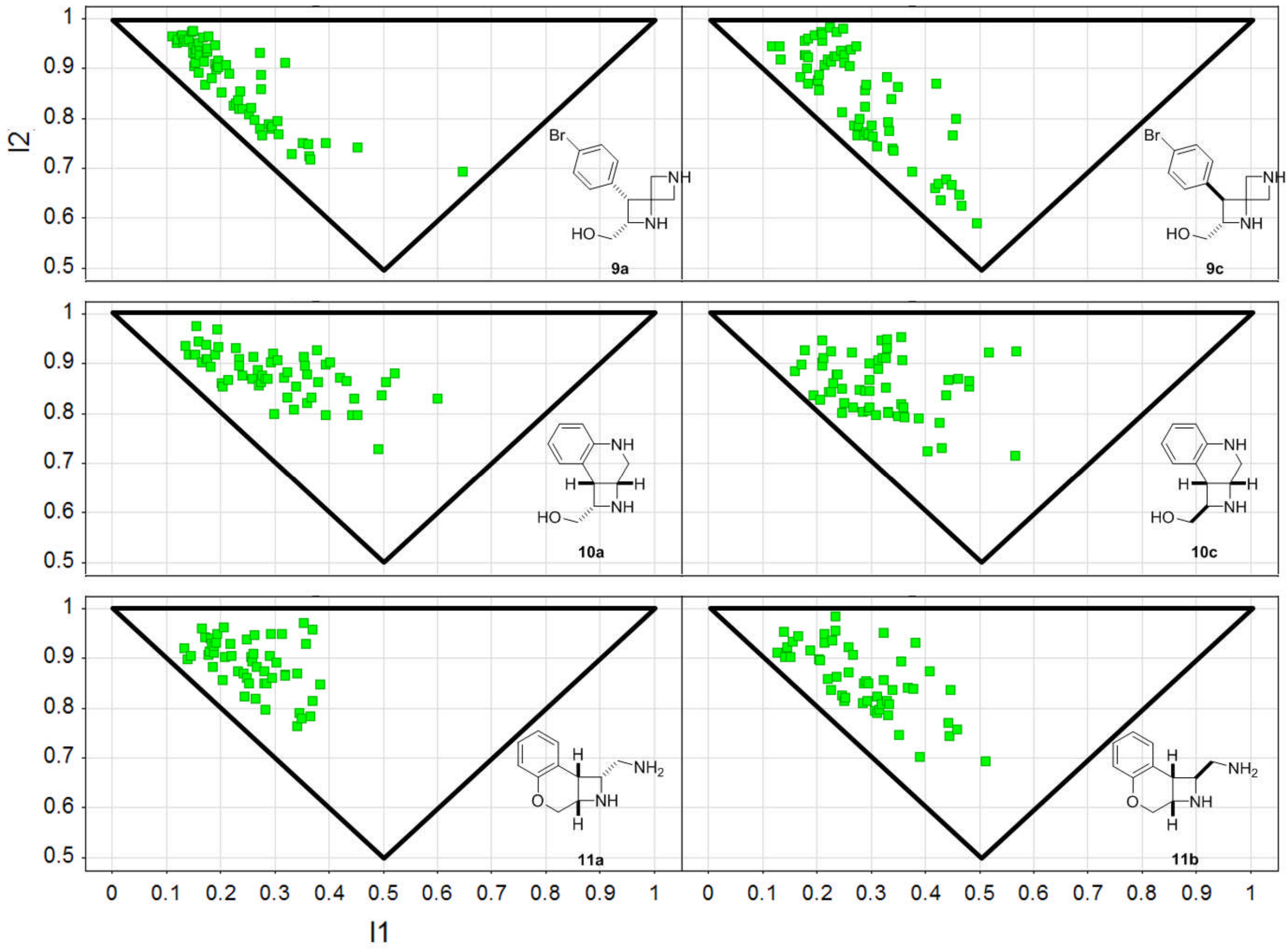

Figure S17. PMI distributions of the 1210 compounds (generated by the process shown in Figure S13) with respect to the parent scaffolds **4-11**.

2.6 Analysis of spirocylic scaffolds 12-13

As summarized in Figure S18, scaffolds **12-13** were decorated once or twice using the Standard LLAMA Capping Groups (SLCGs). The following reaction types were enabled in LLAMA: reductive amination, Suzuki coupling, Buchwald-Hartwig amination, sulfonamide formation, urea formation, alcohol alkylation, carbamate formation, secondary amide alkylation, secondary amide arylation, amide formation, alcohol arylation. A molecular property plot for the entire library (AlogP versus number of Heavy Atoms) is shown in Figure S19, whilst individual molecular property plots for compounds derived from scaffolds **12-13** are shown in Figure S20. A PMI plot (including standard deviations) of the average PMI values for compounds derived from scaffolds **12-13** is shown in Figure S21. The average PMI values are given in Table S6. A PMI plot of low-lying conformers for the 2700 compounds derived through virtual decoration of scaffolds **12-13** are shown in Figure S22, whilst individual plots for each scaffold are shown in Figure S23.

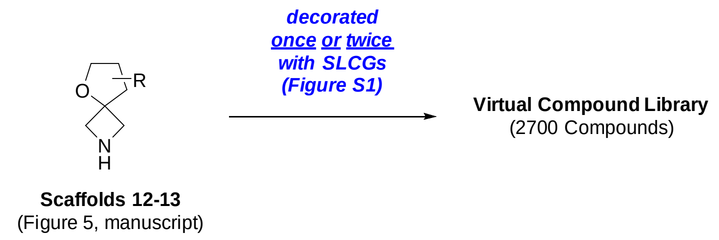

Figure S18. An overview of the decoration process for the azetidine-based scaffolds **12-13**.

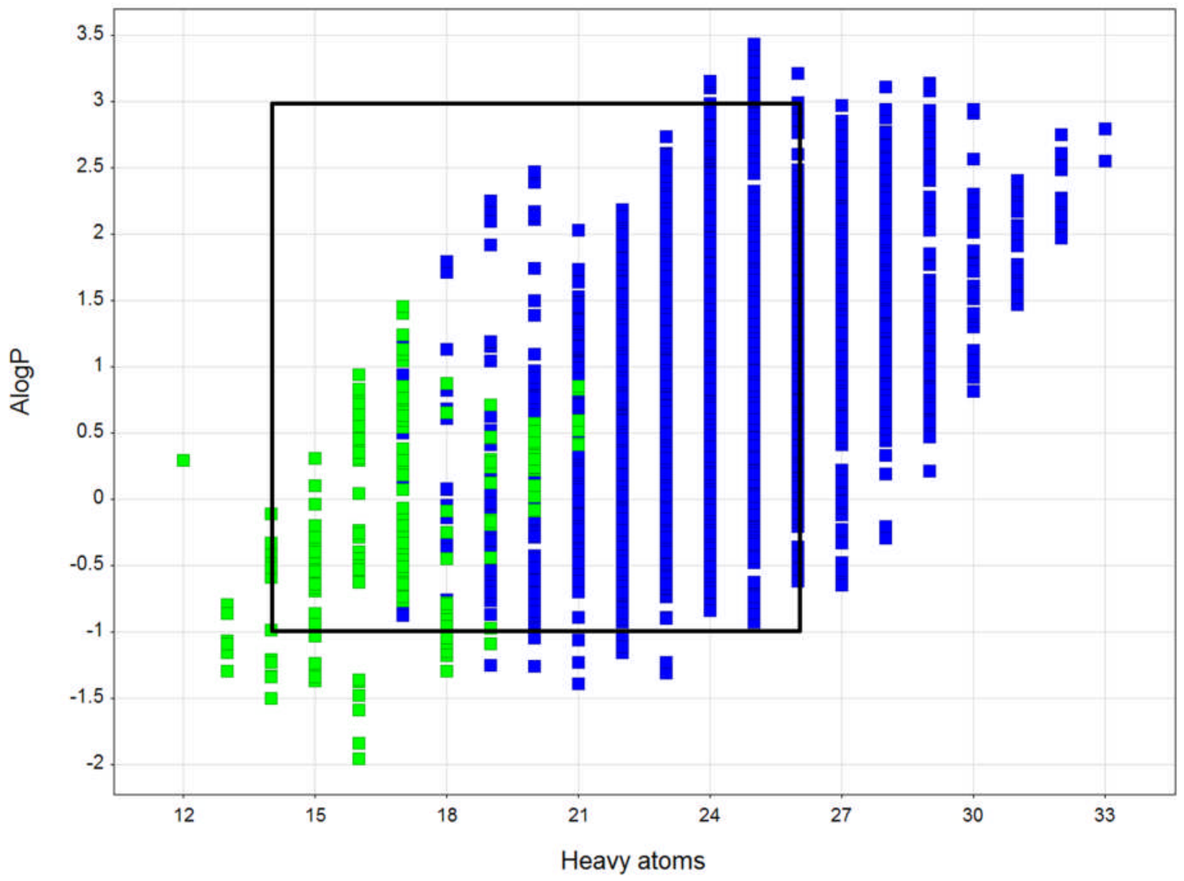

Figure S19. Molecular property analyses of the 2700 virtual compounds derived through decoration of scaffolds **12-13**. The data set includes 182 singly decorated compounds (green squares) and 2518 doubly decorated compounds (blue squares). Lead-like space (−1 ≤ AlogP ≤ 3; 14 ≤ HA ≤ 26) is outlined by the black rectangle. 2074 of the 2700 compounds (77%) are lead-like according to these constraints. 148 of 182 singly decorated compounds (81%) are lead-like; 1926 of 2518 doubly decorated compounds (76%) are lead-like.

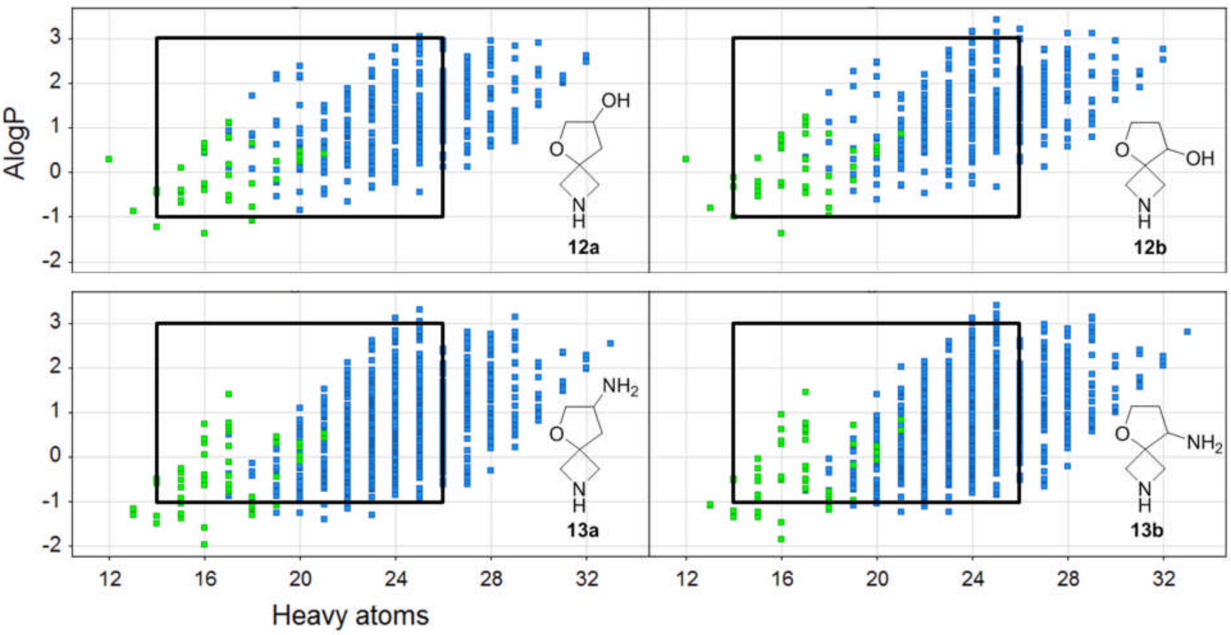

Figure S20. Molecular property analyses of the 2700 virtual compounds with respect to the parent scaffolds **12-13**. Scaffold **12a** has 377 derivatives (39 singly decorated, 338 doubly decorated), of which 284 are lead-like (75%: 33 singly decorated derivatives, 251 doubly decorated derivatives). Scaffold **12b** has 377 derivatives (39 singly decorated, 338 doubly decorated), of which 283 are lead-like (75%: 36 singly decorated derivatives, 247 doubly decorated derivatives). Scaffold **13a** has 973 derivatives (52 singly decorated, 921 doubly decorated), of which 752 are lead-like (77%: 38 singly decorated derivatives, 714 doubly decorated derivatives). Scaffold **13b** has 973 derivatives (52 singly decorated, 921 doubly decorated), of which 755 are lead-like (78%: 41 singly decorated derivatives, 714 doubly decorated derivatives).

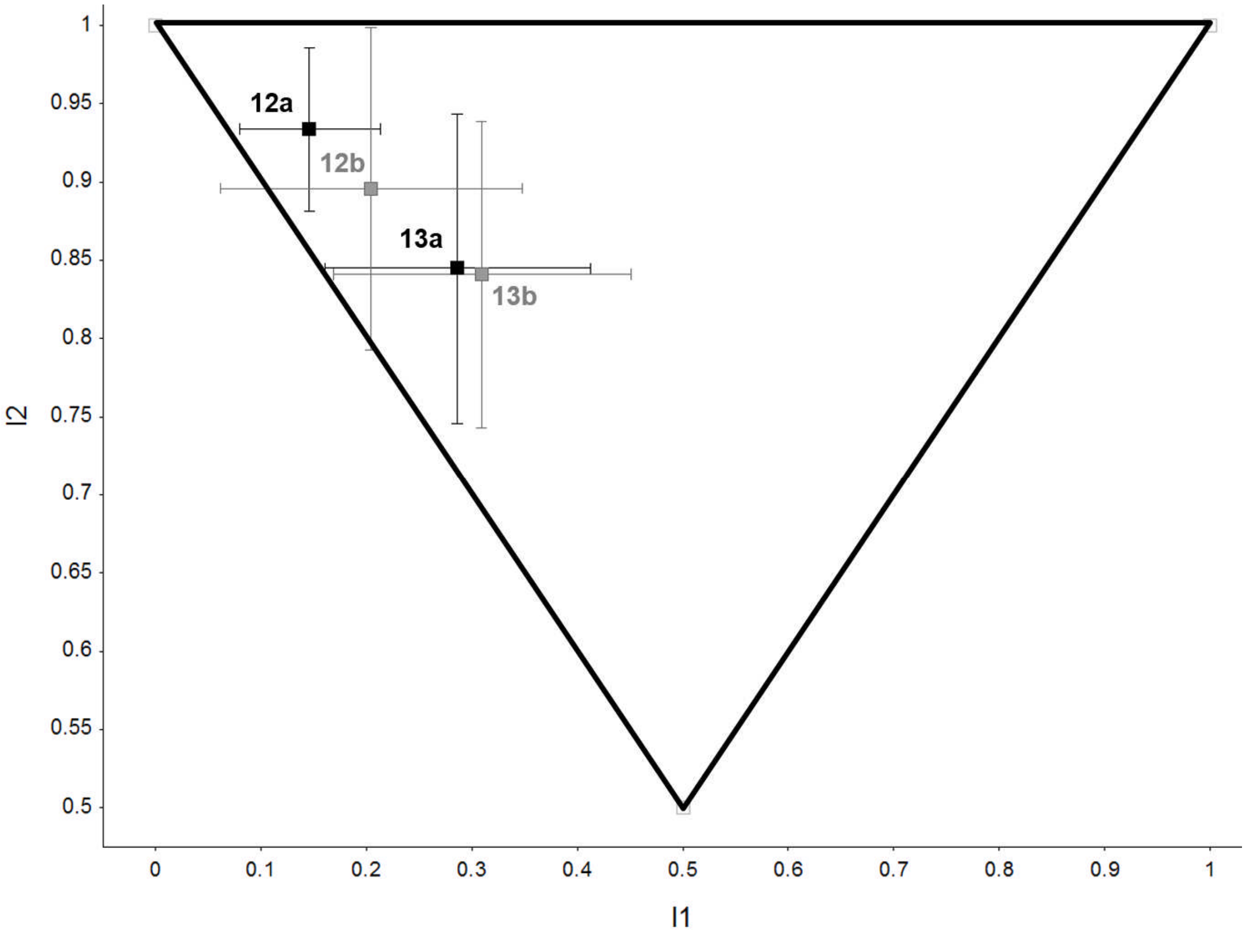

Figure S21. Average PMI values for the compounds derived from scaffolds **12-13** including standard deviations. See table S6 for further information.

Scaffold	No. Derivatives	μI1	μI2	σI1	σI2
**12a**	377	0.146	0.934	0.067	0.052
**12b**	377	0.286	0.845	0.126	0.099
**13a**	973	0.204	0.896	0.143	0.103
**13c**	973	0.309	0.841	0.141	0.098

Table S6. Average PMI values for the compounds derived from scaffolds **12-13** including standard deviations (calculated using Dotmatics Vortex). These values were used to plot Figure 5 (manuscript).

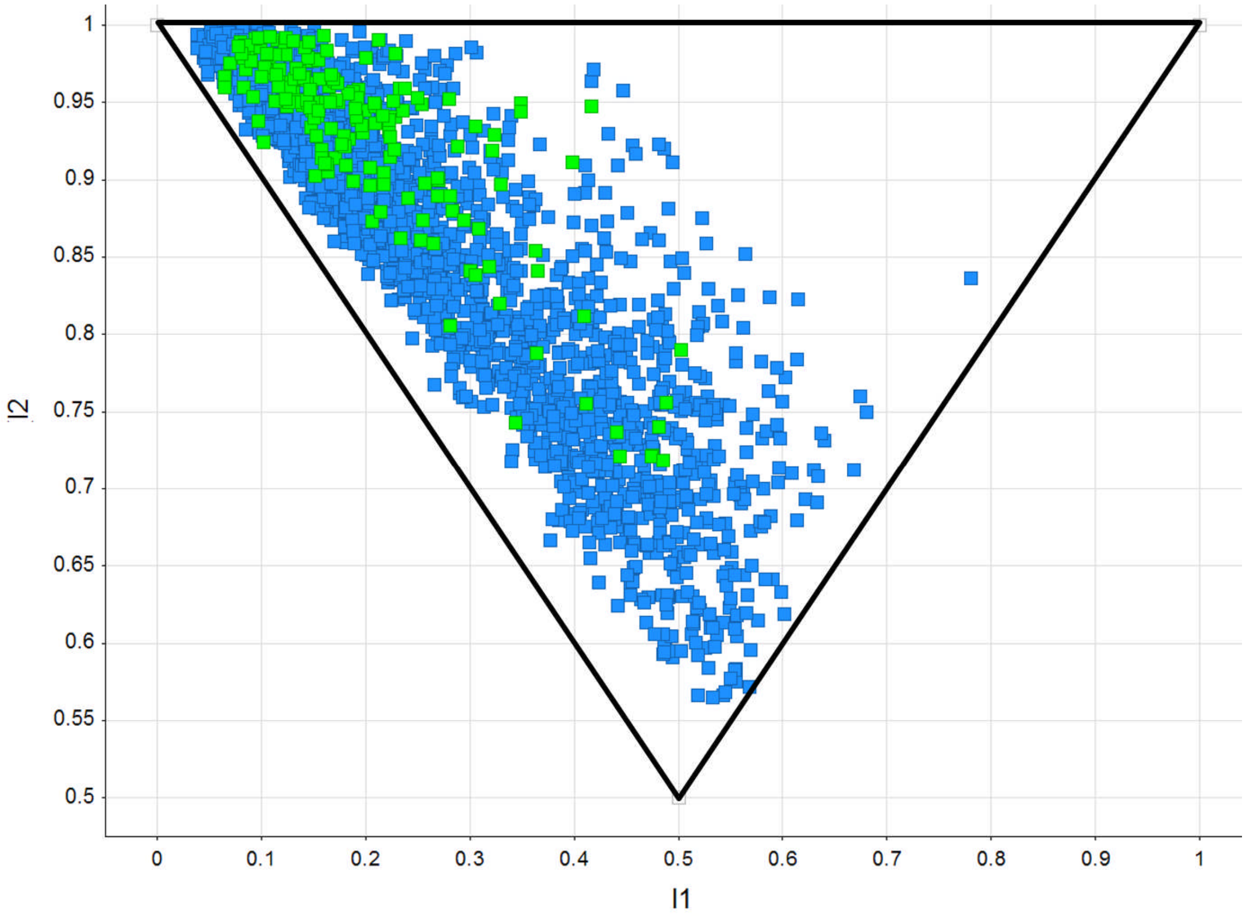

Figure S22. A PMI plot of the 2700 virtual compounds generated in Figure S18 (mean PMI coordinates: I1 = 0.245, I2 = 0.874). Singly decorated derivatives, green squares, have mean PMI coordinates: I1 = 0.197, I2 = 0.930. Doubly decorated derivatives, blue squares, have mean PMI coordinates: I1 = 0.249, I2 = 0.870.

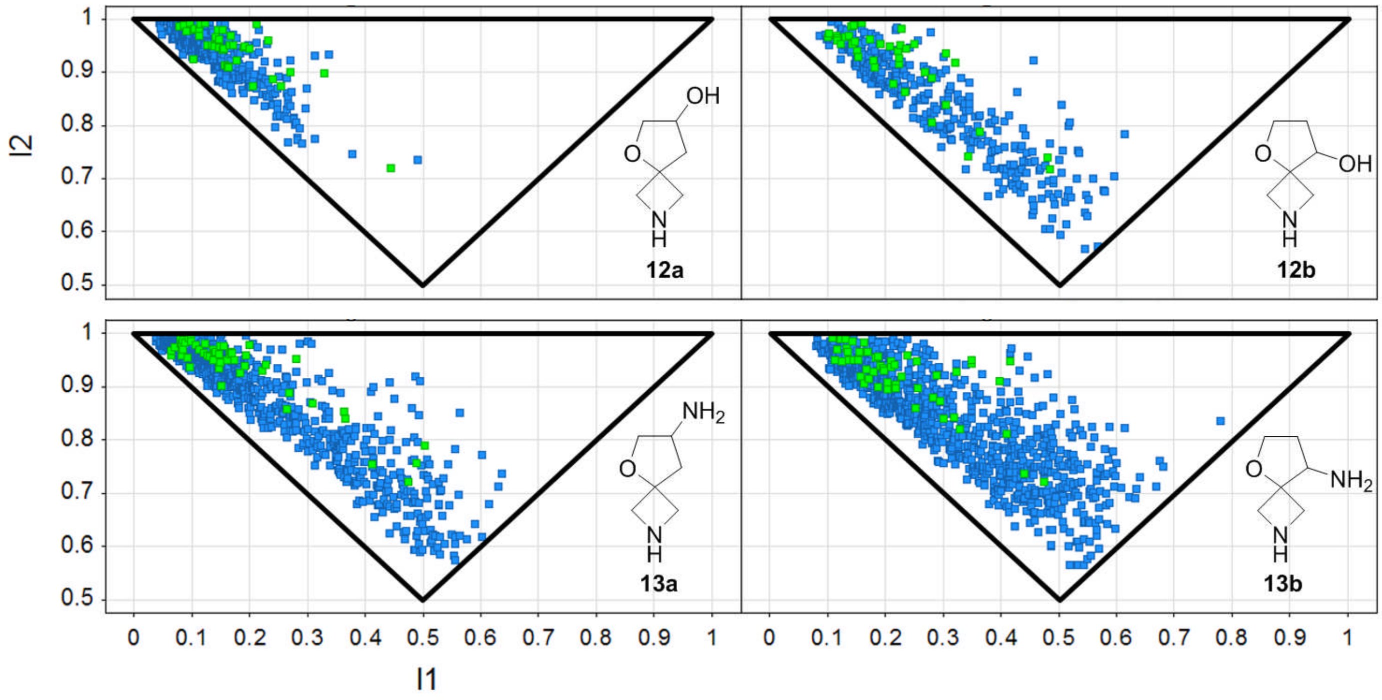

Figure S23. A PMI plot of the 2700 virtual compounds generated in Figure S18 with respect to the parent scaffold **12-13**.

2.7 Analysis of oxetane scaffolds 17a-e

As summarized in Figure S24, virtual decoration of the carboxylic acid functionality in scaffolds **17a-e** with 28 small amines (criteria: ≤8 heavy atoms; commercially available) shown in Figure S25, generated a library of 168 amides. A molecular property plot for the library (AlogP versus number of Heavy Atoms) is shown in Figure S26. Derivatives with (9 ≤ heavy atoms ≤ 17; −1 ≤ AlogP ≤ 3) were considered to be fragment-like.

The 61 fragment-like compounds derived from scaffolds **17a-e** were compared with 257 randomly chosen commercially-available fragments from the eMolecules database and 261 randomly chosen fragments from the GDB-17 database of exhaustively enumerated compounds. Accelrys Pipeline Pilot version 8.5 (Pipeline Pilot v8.5.0.200, Accelrys® Software Inc., 2011) was used to process the selection of these compounds, as shown in Figure S27. The SMILES for the eMolecules compounds are given in Table S7. Plane of best comparison of the three libraries is shown in Figure S28.

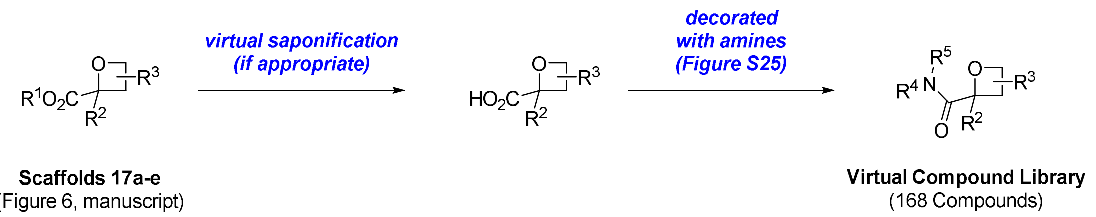

Figure S24. An overview of the decoration process for oxetane-based scaffolds **17a-e**.

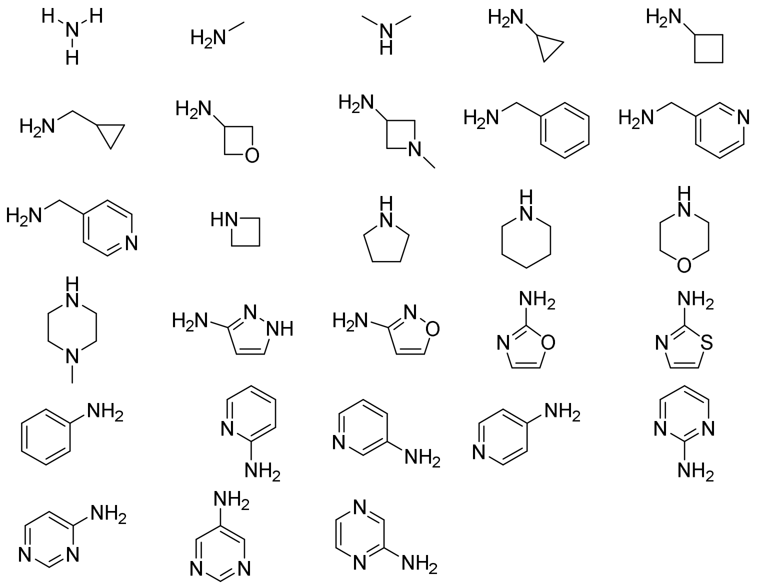

Figure S25. Amines used to decorate scaffolds **17a-e**.

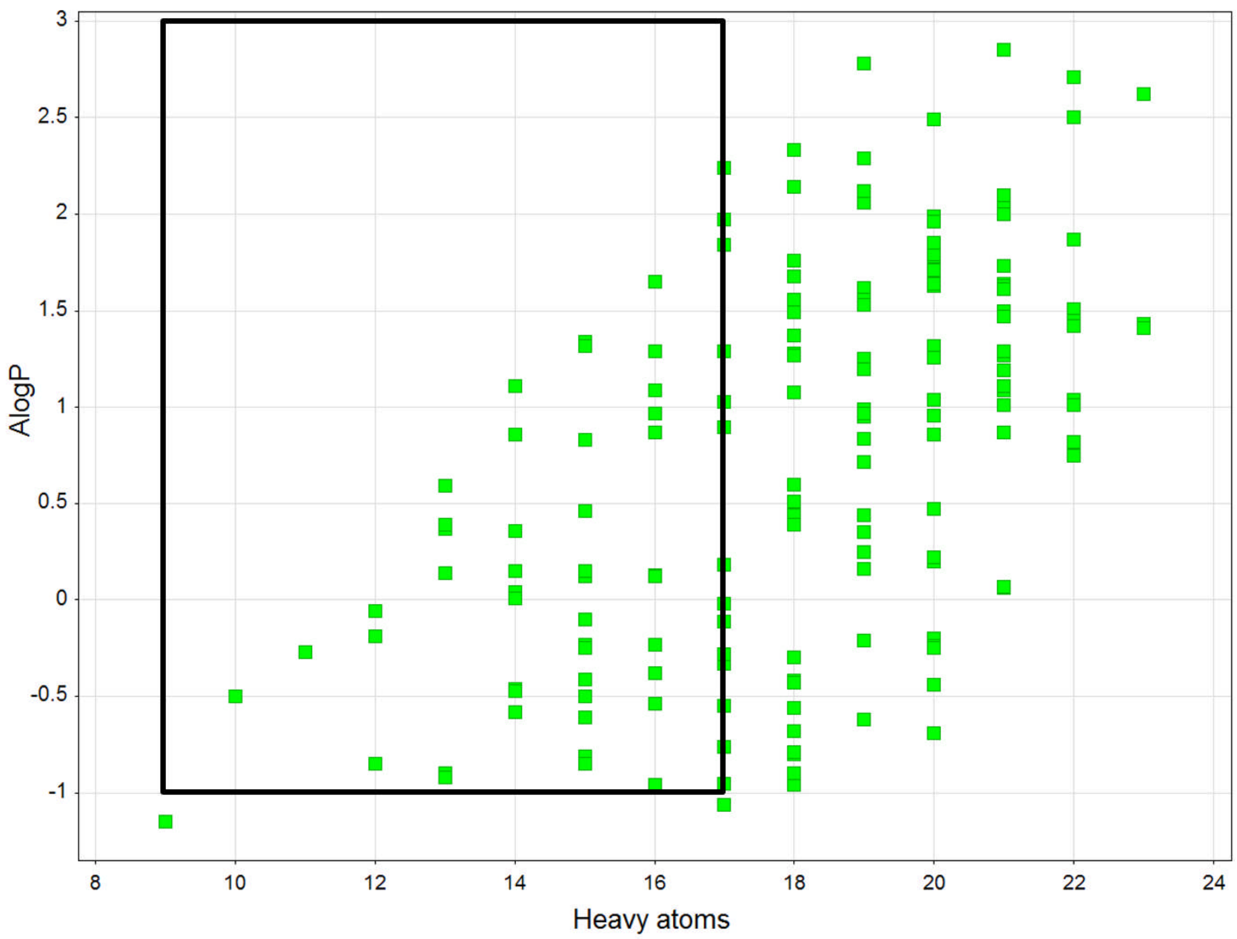

Figure S26. Molecular properties analysis for the 168 compounds generated from scaffolds **17a-e** (Figure S24). The black box highlights the space considered to be fragment-like (9 ≤ heavy atoms ≤ 17; −1 ≤ AlogP ≤ 3), in which 61 of 168 compounds were found (36%).

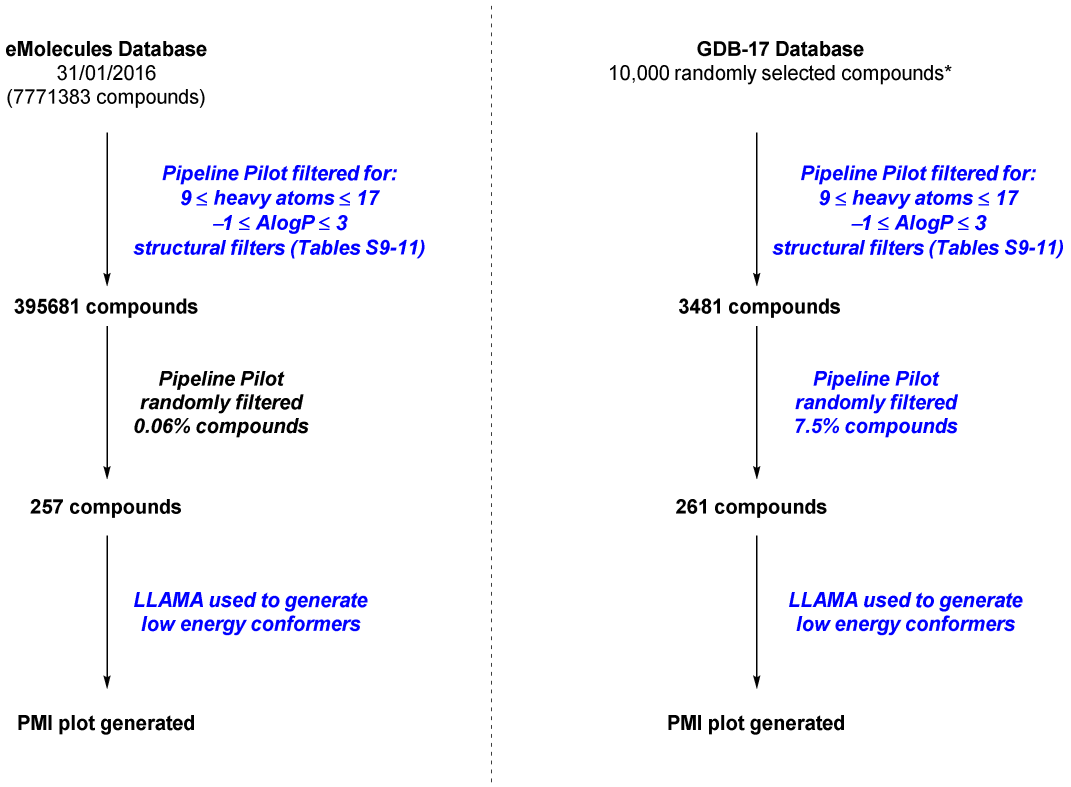

Figure S27. An overview of the enumeration process for selecting compounds from the eMolecules and GDB-17 databases. Pipeline pilot was used to filter the libraries for fragment-like molecular properties and to remove any structural liabilities (further information in Section 2.7.1 and Tables S9-11). The heavy atoms filter counted the following elements: C, N, O, S, F, Cl, Br, I. Libraries of ~260 compounds were randomly selected in Pipeline Pilot. Finally, LLAMA was used to generate low energy 3-D conformers. *The 10,000 GDB-17 compounds were selected at random from a database of 166 billion compounds by Prof. Jean-Louis Reymond and Mahendra Awale (University of Berne) and were provided to us in a personal communication.

Entry	Canonical SMILES
1	OCC1CCCCN1C(=O)CC[n]1c[n]cc1
2	C[n]1c([n][n]c1SCC(N)=O)-c1cc[n]cc1
3	CCC(=O)c1cc(C(O)C(O)=O)c(CO)cc1
4	CCc1[nH]c([n][n]1)S(=O)(=O)CC1CCCCO1
5	COC(=O)C(NC(=O)OC(C)(C)C)C1COC1
6	OC(=O)CCCN1C=Nc2ccccc2C1=O
7	COCCN1Cc2cccc(C(O)=O)c2C1=O
8	CC(C)(C)C(=O)NCC(=O)Nc1c[n]ccc1
9	CN1CCN(CC(N)Cc2ccccc2)CC1
10	Cc1[n]c(CNC(=O)NCc2cc[nH][n]2)c[s]1
11	CCC(C)NC(=O)c1c[n][n](CCC(O)=O)c1
12	Cc1ccc(o1)C(C)(O)CNC(=O)COCC
13	NS(=O)(=O)c1ccc(cc1)OCc1[n]oc[n]1
14	Nc1cc(C#N)c([n]c1OC(F)(F)F)C(N)=O
15	O=C1NCCNc2c1c[n]c1cc(F)ccc12
16	Nc1c[n]c(N)c(OC(F)(F)F)c1CC(O)=O
17	CC(C)CNS(=O)(=O)c1cccc2[n]o[n]c21
18	Cc1[n][n](CC(O)=O)c2[n]ccc(C3CC3)c21
19	COC(=O)C1CCCN1c1cccc(CO)c1
20	COc1ccc([n][n]1)C(=O)N1CC2CC1CC2
21	Cc1[n]cc[n]1Cc1ccc(cc1)NC(N)=O
22	CCNC(=O)N1C2CCCCC2CC1C(O)=O
23	O=S1(=O)COCCN1Cc1cc(Cl)ccc1F
24	Nc1cc(-c2c[n]c(N)c[n]2)c2cc[nH]c2[n]1
25	CSCC(=O)NCCc1[n]oc([n]1)C(F)(F)F
26	COc1c([n]cc(F)c1C(N)=O)OC(F)(F)F
27	O=C(Nc1cc(F)cc(F)c1)C1CCCCN1
28	CS(=O)(=O)N(Cc1cocc1)Cc1ccc[s]1
29	CN(C)S(=O)(=O)NCCCc1cccc(Br)c1
30	CC(=O)NCCOc1ccc2ccccc2[n]1
31	CC(C)(C)CC(=O)O/N=C(/N)c1c[n]ccc1
32	NS(=O)(=O)c1[n]c(O)cc(O)c1OC(F)(F)F
33	CNC(=O)CCc1cc2ccccc2[n]c1O
34	CS(=O)(=O)N1CCN(CC1)c1cccc(Br)c1
35	CN(Cc1ccc(Cl)[s]1)C(=O)C1CCCC1N
36	NS(=O)(=O)NC1(CCCC1)c1cccc(Cl)c1
37	COc1cc(ccc1[C@@H](N)CC#N)C(F)(F)F
38	COc1ccc(cc1)OCCc1[n][n]c(N)[s]1
39	CN(Cc1ccc(cc1)OCC)C(=O)NCC
40	O=C1OC(Nc2cccc[n]2)c2ccccc21
41	CC(C)CCOc1ccc(F)cc1S(N)(=O)=O
42	O=C(Cc1ccc(F)cc1F)c1c[n]ccc1
43	Cc1[n]oc(CNC(=O)c2ccc(Cl)cc2)[n]1
44	CC(C)(C)NCC(=O)Nc1cc(Cl)ccc1F
45	CCSCC[n]1cc(CCO)c([n]1)C(F)(F)F
46	O=C(NC1CCCC1)c1c[n]2CCCCc2[n]1
47	CCc1[n]o[n]c1NC(=O)c1ccccc1F
48	CC(C)(C)[n]1[n]c(c(C#N)c1N)-c1ccco1
49	Cc1cc(NC(=O)Nc2ccccc2)[n]cc1
50	Cc1ccc2cccc(OC(=O)N(C)C)c2[n]1

Entry	Canonical SMILES
51	CN(Cc1ccc(Cl)cc1F)CC1COCC1
52	CN(C(=O)C=Cc1ccco1)c1ccccc1
53	Cc1ccc(cc1)C(OCC(O)=O)C(F)(F)F
54	Cc1o[n]c(C)c1C(=O)NCC1CCCCC1
55	CN1c2ccc(cc2CCC1=O)S(N)(=O)=O
56	CC(=O)Cc1cc(c(cc1)OC)S(N)(=O)=O
57	CO[C@@H]1CN(C[C@H]1NC)C(=O)OC(C)(C)C
58	COc1c(C(=O)OC)c(CN)ccc1C#N
59	CC(=O)Nc1[n][n](c(N)[n]1)-c1ccccc1
60	Nc1cc(c(Cl)cc1)S(=O)(=O)N1CCCC1
61	COc1cc(Br)cc([C@@H]2COCCN2)c1O
62	Cc1c(c[n][n]1C)NS(=O)(=O)c1ccc[s]1
63	Cc1ccc(Cl)cc1N1C(=O)NN=C1CN
64	CC(=O)NCCNC(=O)CSC1CCCC1
65	CN(CC(O)=O)C(=O)CCc1ccccc1
66	COc1ccc(cc1F)C(=O)CCC(O)=O
67	CN(C)/C=C/C(=O)c1cccc2[n]coc12
68	COCCNCCOc1ccc(cc1)OC
69	O=C(Nc1ccc(F)cc1Cl)c1c[n][n][nH]1
70	COc1cc(cc(OC)c1O)[C@@H](N)C(F)F
71	CC(C)(C)OC(=O)N1CC2C=CC(O)C2C1
72	CCOC(=O)c1cc2c(cc1)OC(=O)N2C
73	CCc1[n]c(c[s]1)C(C)NCC(=O)N(C)C
74	COc1cc(ccc1Cl)[C@@H]1COCC(N)=N1
75	CC(C)(CO)[C@@H](N)c1ccc(Br)c(F)c1O
76	O=C1Cc2ccccc2-c2ccc[n]c2N1
77	Nc1cc[n]([n]1)-c1ccc(c[n]1)C(F)(F)F
78	C[C@@H](N)[C@H](O)c1ccc(Cl)cc1C(F)(F)F
79	CC1CN(C2CCOC2=O)c2ccccc21
80	COc1ccc(cc1)/C=C/C(=O)OCC#N
81	COc1ccc(cc1)Oc1ccc(N)c[n]1
82	Cc1cc(NC(C)c2ccco2)[n]c(N)[n]1
83	COc1c(c[n]cc1C(F)(F)F)OC(F)F
84	COc1[n]cc(C#N)c(O)c1OC(F)(F)F
85	OCC1(CCCCC1)NCc1ccccc1
86	COc1cc(ccc1CCC(O)=O)OCC
87	CC(C)(C)c1ccc(cc1)OC/C(/N)=N/O
88	Nc1cc(ccc1)-[n]1[n]ccc1C(F)(F)F
89	CCOC(=O)CC(=O)c1cc(Br)c(F)cc1
90	N#CCOC(=O)c1cc2c(F)cccc2[s]1
91	CCOC(=O)Cc1[n]c2ccc(F)cc2o1
92	N#CCc1[n]ccc(c1O)-c1ccccc1
93	OC(=O)c1c[nH]c2c1c(F)c(F)c(F)c2F
94	Brc1ccc[n]2c([n][n]c21)-c1ccccc1
95	Cc1ccc2NCC(Cc2c1)C(=O)OCC
96	COC1=CC=CN(Cc2ccc(Cl)[s]2)C1=O
97	OC(=O)c1c[n]c2NC(=O)NC(=O)c2c1
98	COCCC1(CCS(=O)(=O)CC1)C(O)=O
99	CCNC1CN(CCC1)CCS(N)(=O)=O
100	CCOC(=O)c1cc2C(=O)NC=N[n]2c1
101	OS(=O)(=O)c1ccc(Cl)c2ccc[n]c12
102	CC(=O)Nc1ccccc1OCC(N)=O

Entry	Canonical SMILES
103	CC(C)CC1NC(=O)C2CCCN2C1=O
104	N[C@@]1(CO)C(=O)Nc2cc(Cl)c(F)cc12
105	CCOC(=O)c1cc2c(N)[n]c[n]c2[nH]1
106	CCC(CCO)NC(=O)Nc1cco[n]1
107	N#CCC(=O)NCc1ccc(F)cc1F
108	CCCNC(N)=NCCc1cc[n][n]1C
109	CC(C)C[C@@H](NC(=O)NCC=C)C(O)=O
110	COCCOc1cc(O)c([s]1)C(=O)OC
111	Cc1cc(NCc2cc[n][n]2C)[n][n]1C
112	Cc1cc(ccc1)C(=O)Nc1[n][nH]c[n]1
113	COCCNC(=O)Nc1ccc(F)cc1
114	COC(=O)c1cc([n]cc1)-c1cc[nH][n]1
115	COc1ccc(c[n]1)[C@@H](N)CC(F)(F)F
116	COc1cccc2cc(c[n]c21)C(N)=O
117	OCc1cc([n]c(O)c1O)OC(F)(F)F
118	NCc1cc[n][n]1CCc1ccccc1
119	Oc1c[n]cc(CC(O)=O)c1C(F)(F)F
120	Cc1ccc([n][n]1)-c1ccc(CO)cc1
121	CCN(Cc1ccccc1)C(=O)CC#N
122	CSC1CC(CC1)N(C)CCS(C)(=O)=O
123	C1NCC21CC(C2)OCc1ccccc1
124	Cc1cc(C)[n]2c([n][n]c2[n]1)SCC=C
125	CC(C)=CC(=O)NC(C)COCC(C)C
126	CSc1ccc(CCC(O)=O)c(c1)C#N
127	Cc1cc(c[n]c1NC)C1CCCN1C
128	OC1CN(Cc2cc(Cl)ccc2F)CC1
129	COc1ccc(Cl)c(O)c1[C@@H](N)C1CC1
130	CC(NC(=O)C(N)CC)c1ccc(Cl)[s]1
131	CNCc1ccc(cc1)-c1cccc[n]1
132	CCCC[C@@H](O)[C@@H](N)c1ccccc1F
133	CC(C)(O)CNCc1cc(Br)ccc1F
134	COC(=O)c1cccc2oc([n]c12)SC
135	Cc1cc(CNCc2ccco2)ccc1
136	CCOc1ccc(cc1)-c1[n]c(N)[n]o1
137	Cc1[n]c(c[s]1)C(C(F)(F)F)S(N)(=O)=O
138	C(NC1CC1)c1cc2ccc[n]c2cc1
139	CCOc1cc(CN)cc2ccccc21
140	OC(=O)c1cc(F)c(c(F)c1)C(F)(F)F
141	OC(=O)Cc1cc(F)c(cc1)C(F)(F)F
142	Nc1cc([n]o1)-c1cc2CCCc2cc1
143	Nc1cc([nH][n]1)C(=O)N1CCOCC1
144	NC(=O)c1cccc2[n]c(CN)oc21
145	CC(C)NCc1cc(c[nH]1)S(N)(=O)=O
146	Nc1cccc2c1CCN2S(N)(=O)=O
147	CCN(Cc1cc[n]cc1)C1CNC1
148	CCCOCc1cc(O)[n]c(CO)[n]1
149	CNCc1ccc(O)c2[n]cccc21
150	Nc1[n]c(cc(N)[n+]1[O-])N1CCCC1
151	NC(=O)CSc1[n][n]c(NC2CC2)[s]1
152	CS(=O)(=O)c1ccc(F)cc1C(O)=O
153	N[C@@H]1CCOc2c(F)cc(cc21)C#N
154	Cc1ccc(c[n]1)[C@@H](O)[C@@H](N)CCC

Entry	Canonical SMILES
155	CNC(C(N)=O)c1cc(Br)ccc1F
156	Cc1cc(NS(=O)(=O)CC)[n](CC)[n]1
157	Cc1[n][nH]cc1CNCCCOCC
158	CC(=O)c1[n]c2ccc(cc2o1)C#N
159	COC(=O)c1c[n]c(cc1)NC1CC1
160	CCC(C)N(CCN)Cc1ccco1
161	COc1cc2C=CC(=O)N(C)c2cc1
162	CC(C)(C)C(=O)NCC1CCNCC1
163	Oc1cc(Br)c(F)cc1C(O)C(O)=O
164	Cc1c2c(CC2=O)c(C)c2c1CC2=O
165	COc1ccc2CCC(C(O)=O)c2c1
166	CCOC(=O)c1cc(O)ccc1C#N
167	CC(=O)c1cccc(c1N)C(F)(F)F
168	CCS(=O)(=O)Nc1ccc(CC)cc1
169	OC(=O)c1cc(F)c[n]c1C(F)(F)F
170	CC=CC(=O)c1cc2OCOc2cc1
171	C/C(=C/C(=O)OC)/Nc1ccccc1
172	CC(C)N(CCNC1CCC1)C(C)C
173	COc1cc(ccc1)C1(CC1)C(C)O
174	Nc1cc[n]c(OCC2CCC2)c1Br
175	Cc1cc2[nH]cc(C(O)=O)c2cc1Cl
176	NC(c1ccc[s]1)c1cc(F)ccc1
177	COC(=O)Cc1ccc(Br)c(F)c1F
178	Cc1[n][n]c(SCC)c2ccccc12
179	NCCC(=O)NCc1cccc[n]1
180	OC(=O)c1cccc2c[n][n]cc21
181	C(c1cc[n]cc1)N1CCNCC1
182	C[n]1[n][n]c2cc(ccc12)C(N)=O
183	C[n]1c([n]c2c[n]ccc12)C(C)N
184	OC(=O)c1c[s]c2N=CNC(=O)c12
185	CN(C)Cc1ccc(cc1)C(N)=N
186	NCC1CN(Cc2ccco2)CC1
187	O=C1CC2C3CC(=O)C4C1C2CC34
188	OC(=O)Cc1cc2cccc[n]2[n]1
189	COC(=O)C(CN)c1ccccc1
190	O=C(CC1CCNCC1)NC1CC1
191	CC1CNCCN1c1[n]ccc[n]1
192	Cc1ccc(F)c(O)c1[C@@H](N)CF
193	CNC(=O)Nc1cc(C)cc(C)[n]1
194	COc1c(F)c(ccc1CN)OC
195	CC(=O)c1cccc2c1c[n][n]2C
196	CC(=O)NC1CC(C)(C)CC(=O)C=1
197	CCCCOc1[n]cccc1CN
198	C/C(=C(/C#N)C(N)=O)/c1ccc[s]1
199	Cc1ccc(c(C#N)[n]1)C(=O)OC
200	FC[C@@H](N1CCOCC1)C(F)(F)F
201	N[C@@H](CF)c1ccc(F)c(F)c1F
202	Cc1ccc(c(O)[n]1)-c1cc[n][nH]1
203	Cc1cc(ccc1)NCCC(N)=O
204	Cc1cc(O)c(c(c1)C#N)C(O)=O
205	C[C@@H](O)[C@@H](N)c1cc(Cl)cc(F)c1
206	CCCCNc1ccc(CO)c[n]1

Entry	Canonical SMILES
207	OCc1cccc([n]1)N1CCCC1
208	COc1ccc(Br)cc1S(C)(=O)=O
209	O=C1Cc2cccc3CCCN1c23
210	Oc1cc[n]c([n]1)N1CCCCC1
211	CCOc1cc[n]c2ccccc12
212	N#Cc1cc(C(O)=O)c(Cl)cc1Cl
213	N[C@@H](CC1CC1)c1ccccc1F
214	Cc1ccccc1C(C)(C)C(O)=O
215	CC(C)(C)C(N)c1ccc(F)cc1
216	CC[n]1[n]cc(N)c1C(=O)NC
217	Cc1[n]c([nH][n]1)C1CCNCC1
218	CC(C)CN1CC2NOCC2C1
219	Cc1cc(CC(O)=O)c(F)c[n]1
220	CSC1=NC(=O)NC2CCCC=21
221	N#CC1C(=O)NC=C2CCCC2=1
222	CC(N)c1c[n]2ccccc2[n]1
223	Cc1[n][nH]c(C)c1NC(=O)CC
224	CC#CCCNC(=O)NC(C)C
225	NC(=O)c1cc(Br)cc(F)c1F
226	OCc1ccc2[nH]c[n]c2c1Br
227	Cc1cc(ccc1F)[C@@H](N)CF
228	Nc1c(Br)cc(F)cc1C(O)=O
229	Nc1cc(ccc1O)C(F)(F)F
230	C=C[C@@H](N)c1cccc(Cl)c1F
231	COc1[n]cc(Cl)cc1OCC
232	FC(F)(F)C(F)(F)COC(F)F
233	CC(C)c1cc(oc1)[C@@H](N)CC
234	CCOC(=O)C=Cc1cc[s]c1
235	C[C@@H](CC(O)=O)Sc1ccc[s]1
236	OC1CCCc2c(Br)cccc21
237	NCC(O)c1ccc(O)cc1
238	COC(=O)c1[n]c(N)cc[n]1
239	OC(=O)CC1CS(O)(O)CC1
240	CC(N)(CN)c1cc[n]cc1
241	Cc1[n]c(COC)[s]c1CO
242	N[C@@H]1C[C@H]1c1ccc(F)cc1
243	O=C1NCc2ccc(Cl)cc21
244	CC(=O)c1cc(CC#N)c[s]1
245	COc1c[n]c(C#N)c(Br)c1
246	COc1cc(CO)ccc1Br
247	NCCC1CS(=O)(=O)CC1
248	CC[n]1cc[n]c1NCC
249	COc1[n]cc(Br)cc1O
250	Oc1c(F)cc(Cl)cc1F
251	CC1CNCC(CO)O1
252	CC(C)[C@@H]1CNCCN1
253	Cc1cc[n](CCO)[n]1
254	CNCC(O)C(F)(F)F
255	Cc1[s]c(C)cc1CN
256	Fc1c[n]cc(Cl)c1F
257	Oc1cccc(Br)c1F

Table S7. Canonical SMILES for the 257 randomly-selected compounds from the eMolecules Database.

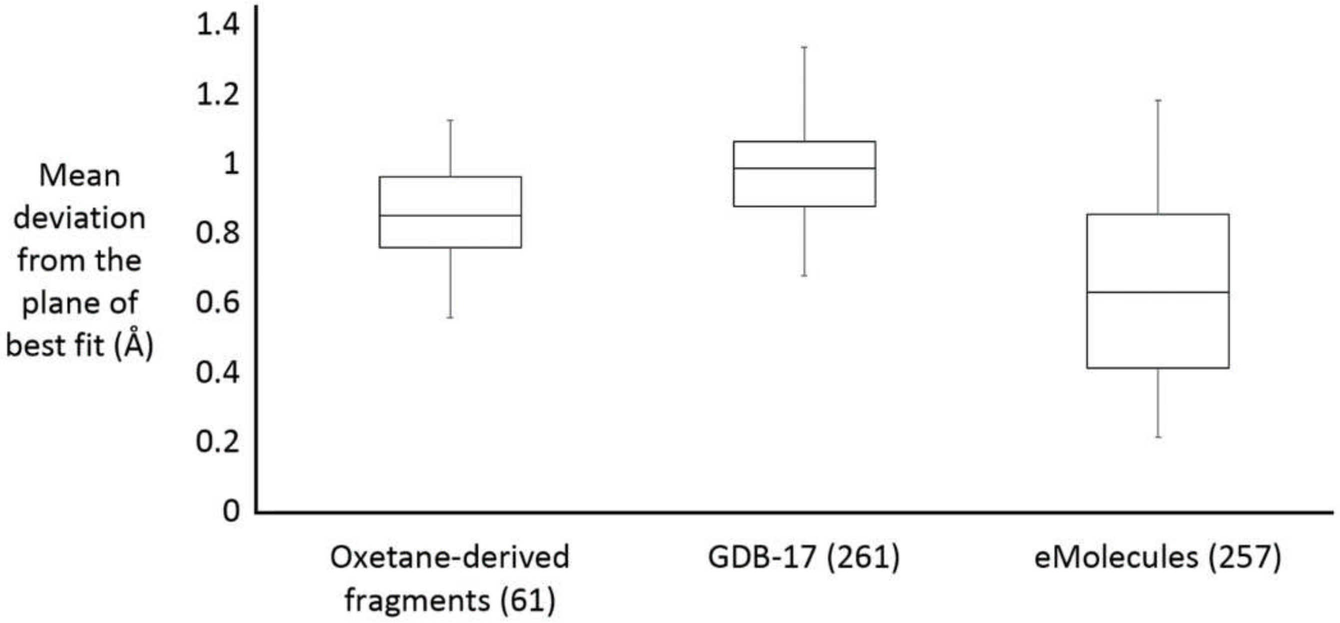

Figure S28. Box and whisker plots to show the deviation from the plane of best fit[2]; for the fragments derived from oxetanes **17a-e**, versus the randomly selected, fragment-like, compounds from the GDB-17 and eMolecules databases. The range of values is shown (bar) together with median, first and third quartile values (box).

2.7.1 Substructure filters

Structural filtering was performed by interrogating two sets of SMARTS definitions using the substructure search tool within Pipeline Pilot. The first set contained 240 definitions (Table S8) as compiled by Shoichet, Simeonev *et al.* and used at the NIH Chemical Genomics Centre.[3] The second set contained 36 definitions (Table S9) and are examples from the 'GSKB' filter as described by Churcher *et al*.[4] In addition, the structural element of the high throughput screening filter embedded in Pipeline Pilot was also used, which comprises the filters for undesirable functionality outlined in Table S10.

Filter	SMARTS
2,3,4-trihydroxyphenyl	c([OH])c([OH])c([OH])
2,4,5-trihydroxyphenyl	c([OH])c([OH])cc([OH])
2halo_pyrazine_3EWG	[#7;R1]1[#6]([F,Cl,Br,I])[#6]([$(S(=O)(=O)),$(C(F)(F)(F)),$(C#N),$(N(=O)(=O)),$([N+](=O)[O-]),$(C=O)])[#7][#6][#6]1
2halo_pyrazine_5EWG	[#7;R1]1[#6]([F,Cl,Br,I])[#6;!$(c-N)][#7][#6]([$(S(=O)(=O)),$(C(F)(F)(F)),$(C#N),$(N(=O)(=O)),$([N+](=O)[O-]),$(C=O)])[#6;!$(c-N)]1
2halo_pyridazine_3EWG	[#7;R1]1[#6]([F,Cl,Br,I])[#6]([$(S(=O)(=O)),$(C(F)(F)(F)),$(C#N),$(N(=O)(=O)),$([N+](=O)[O-]),$(C=O)])[#6][#6][#7]1
2halo_pyridazine_5EWG	[#7;R1]1[#6]([F,Cl,Br,I])[#6][#6][#6]([$(S(=O)(=O)),$(C(F)(F)(F)),$(C#N),$(N(=O)(=O)),$([N+](=O)[O-]),$(C=O)])[#7]1
2halo_pyridine_3EWG	[#7;R1]1[#6;!$(c=O)]([F,Cl,Br,I])[#6]([$(S(=O)(=O)),$(C(F)(F)(F)),$(C#N),$(N(=O)(=O)),$([N+](=O)[O-]),$(C=O)])[#6;!$(c-N)][#6][#6;!$(c-N)]1
2halo_pyridine_5EWG	[#7;R1]1[#6;!$(c=O)]([F,Cl,Br,I])[#6][#6;!$(c-N)][#6]([$(S(=O)(=O)),$(C(F)(F)(F)),$(C#N),$(N(=O)(=O)),$([N+](=O)[O-]),$(C=O)])[#6;!$(c=O);!$(c-N)]1
2halo_pyrimidine_5EWG	[#7;R1]1[#6]([F,Cl,Br,I])[#7][#6][#6]([$(S(=O)(=O)),$(C(F)(F)(F)),$(C#N),$(N(=O)(=O)),$([N+](=O)[O-]),$(C=O)])[#6]1
2-Halopyridine	[F,Cl,Br]-c1n[c,n][c,n][c,n][c,n]1
3halo_pyridazine_2EWG	[#7;R1]1[#6]([$(S(=O)(=O)),$(C(F)(F)(F)),$(C#N),$(N(=O)(=O)),$([N+](=O)[O-]),$(C=O)])[#6]([F,Cl,Br,I])[#6][#6][#7]1
3halo_pyridazine_4EWG	[#7;R1]1[#6][#6]([F,Cl,Br,I])[#6]([$(S(=O)(=O)),$(C(F)(F)(F)),$(C#N),$(N(=O)(=O)),$([N+](=O)[O-]),$(C=O)])[#6][#7]1
4_pyridone_3_5_EWG	[#7,#8,#16]1~[#6;H]~[#6]([$(S(=O)(=O)),$(C(F)(F)(F)),$(C#N),$(N(=O)(=O)),$([N+](=O)[O-]),$(C=O)])~[#6](=O)~[#6]([$(S(=O)(=O)),$(C(F)(F)(F)),$(C#N),$(N(=O)(=O)),$([N+](=O)[O-]),$(C=O)])~[#6;H]1
4halo_pyridine_3EWG	[#7;R1]1[#6;!$(c=O);!$(c-N)][#6]([$(S(=O)(=O)),$(C(F)(F)(F)),$(C#N),$(N(=O)(=O)),$([N+](=O)[O-]),$(C=O)])[#6]([F,Cl,Br,I])[#6][#6;!$(c=O);!$(c-N)]1
4halo_pyrimidine_2_6EWG	[#7]1[#6]([$(S(=O)(=O)),$(C(F)(F)(F)),$(C#N),$(N(=O)(=O)),$([N+](=O)[O-]),$(C=O)])[#7;R1][#6]([F,Cl,Br,I])[#6][#6]1([$(S(=O)(=O)),$(C(F)(F)(F)),$(C#N),$(N(=O)(=O)),$([N+](=O)[O-]),$(C=O)])
4halo_pyrimidine_5EWG	[#7]1[#6][#7;R1][#6]([F,Cl,Br,I])[#6]([$(S(=O)(=O)),$(C(F)(F)(F)),$(C#N),$(N(=O)(=O)),$([N+](=O)[O-]),$(C=O)])[#6]1
acetal	[#6]-O[CH1](-[#6])O[#6]
acid_halide	[S,C](=[O,S])[F,Br,Cl,I]
acrylate	[CH2]=[C;!$(C-N);!$(C-O)]C(=O)
activated_4mem_ring	[#6]1~[$(C(=O)),$(S(=O))]~[O,S,N]~[$(C(=O)),$(S(=O))]1
activated_acetylene	[$(S(=O)(=O)),$(C(F)(F)(F)),$(C#N),$(N(=O)(=O)),$([N+](=O)[O-]),$(C(=O))]C#[C;!$(C-N);!$(C-n)]

Filter	SMARTS
activated_diazo	[N;!R]([$(S(=O)(=O)),$(C(F)(F)(F)),$(C#N),$(N(=O)(=O)),$([N+](=O)[O-]),$(C(=O))])=[N;!R]([$(S(=O)(=O)),$(C(F)(F)(F)),$(C#N),$(N(=O)(=O)),$([N+](=O)[O-]),$(C(=O))])
activated_S#O_3_ring	C1~[O,S]~[C,N,O,S]1[a,N,O,S]
activated_vinyl_ester	O=COC=[$(C(S(=O)(=O))),$(C(C(F)(F)(F))),$(C(C#N)),$(C(N(=O)(=O))),$(C([N+](=O)[O-])),$(C(C(=O)));!$(C(N))]
activated_vinyl_sulfonate	O(-S(=O)(=O))C=[$(C(S(=O)(=O))),$(C(C(F)(F)(F))),$(C(C#N)),$(C(N(=O)(=O))),$(C([N+](=O)[O-])),$(C(C(=O)));!$(C(N))]
acyclic_imide	[C,c][C;!R](=O)[N;!R][C;!R](=O)[C,c]
acyl_123_triazole	[#7;R1]1~[#7;R1]~[#7;R1](-C(=O))~[#6]~[#6]1
acyl_134_triazole	[#7]1~[#7]~[#6]~[#7](-C(=O)[!N])~[#6]1
acyl_activated_NO	O=C(-[!N])O[$([#7;+]),$(N(C=[O,S,N])(C=[O,S,N]))]
acyl_cyanide	C(=O)-C#N
acyl_imidazole	[C;!$(C-N)](=O)[#7]1[#6;H1,$([#6]([*;!R]))][#7][#6;H1,$([#6]([*;!R]))][#6;H1,$([#6]([*;!R]))]1
acyl_pyrazole	[C;!$(C-N)](=O)[#7]1[#7][#6;H1,$([#6]([*;!R]))][#6;H1,$([#6]([*;!R]))][#6;H1,$([#6]([*;!R]))]1
aldehyde	[C,c][C;H1](=O)
aliphatic_chain_6	[CD2;R0][CD2;R0][CD2;R0][CD2;R0][CD2;R0][CD2;R0]
alkynyl_michael_acceptor1	[#6]-C#CC(=O)-[#6,#7,#8]
alkynyl_michael_acceptor2	[CH1]#CC(=O)-[#6,#7,#8]
allene	*=C=*
alpha_dicarbonyl	C(=O)!@C(=O)
alpha_halo_amine	[F,Cl,Br,I,$(O(S(=O)(=O)))]-[CH,CH2;!$(CF2)]-[N,n]
alpha_halo_carbonyl	C(=O)([CH,CH2][Cl,Br,I,$(O(S(=O)(=O)))])
alpha_halo_EWG	[$(C(F)(F)(F)),$(C#N),$(N(=O)(=O)),$([N+](=O)[O-])]-[CH,CH2]-[Cl,Br,I,$(O(S(=O)(=O)))]
alpha_halo_heteroatom	[N,n,O,S;!$(S(=O)(=O))]-[CH,CH2;!$(CF2)][F,Cl,Br,I,$(O(S(=O)(=O)))]
alpha_halo_heteroatom_tert	[N,n,O,S;!$(S(=O)(=O))]-C([Cl,Br,I,$(O(S(=O)(=O)))])(C)(C)
anhydride	[$(C(=O)),$(C(=S))]-[O,S]-[$(C(=O)),$(C(=S)),$(C(=[N;!R])),$(C(=N(-[C;X4])))]
aromatic_azide c	N=[N+]=[N-]
aryl_phosphonate	P(=O)-[O;!R]-a
aryl_thiocarbonyl	a-[S;X2;!R]-[C;!R](=O)
azide	[$(N#[N+]-[N-]),$([N-]=[N+]=N)]
aziridine_diazirine	[C,N]1~[C,N]~N~1
azo_amino	[N]=[N;!R]-[N]
azo_aryl	c[N;!R;!+]=[N;!R;!+]-c
azo_filter1	[N;!R]=[N;!R]-[N]=[*]
azo_filter2	[N;!$(N-S(=O)(=O));!$(N-C=O)]-[N;!r3;!$(N-S(=O)(=O));!$(N-C=O)]-[N;!$(N-S(=O)(=O));!$(N-C=O)]
azo_filter3	[N;!R]-[N;!R]-[N;!R]
azo_filter4	a-N=N-[N;H2]
azoalkanal	[N;R0]=[N;R0]CC=O
azocyanamide	[N;R0]=[N;R0]C#N
bad_boron	[B-,BH2,BH3,$(B(F)(F))]
bad_cations	[C+,F+,Cl+,Br+,I+,Se+]
b-carbonyl_quaternary_nitrogen	C(=O)CC[N+,n+]
benzhydrol	[OH1]-C(-c1ccccc1)-c2ccccc2
benzidine_like	c([N;!+])1ccc(c2ccc([N;!+])cc2)cc1
benzylic-quaternary_nitrogen	cC[N+]
beta_lactam	C1(=O)~[#6]~[#6]N1
beta_lactone	[#6,#15,#16]1(=O)~[#6]~[#6]~[#8,#16]1
betalactam_EWG	C1(=O)~[#6]~[#6]N1([$(S(=O)(=O)[C,c,O&D2]),$(C(F)(F)(F)),$(C#N),$(N(=O)(=O)),$([N+](=O)[O-

Filter	SMARTS
	]),$(C(=O)[C,c,O&D2])])
bis_activated_aryl_ester	O=[C,S]Oc1aaa([$(S(=O)(=O)),$(C(F)(F)(F)),$(C#N),$(N(=O)(=O)),$([N+](=O)[O-]),$(C(=O)O),$(C(=O)N)])aa([$(S(=O)(=O)),$(C(F)(F)(F)),$(C#N),$(N(=O)(=O)),$([N+](=O)[O-]),$(C(=O)O),$(C(=O)N)])1
bis_keto_olefin	CC(=O)[$([C&H1]),$(C-F),$(C-Cl),$(C-Br),$(C-I)]=[$([C&H1]),$(C-F),$(C-Cl),$(C-Br),$(C-I)]C(=O)C
boron_wAr-Head	[C,c]~[#5]
branched_polycyclic_aromatic	a1(a2aa(a3aaaaa3)aa(a4aaaaa4)a2)aaaaa1
carbazide	O=*N=[N+]=[N-]
carbodiimide_isothiocyanate	N=C=[N,O,S]
carbonyl_halide	O=C[F,Cl,Br,I]
chloramidine	[Cl]C([C&R0])=N
crown_ether	[$([O,S,#7;R1;r9,r10,r11,r12,r13,r14,r15,r16,r17,r18][CH,CH2;r9,r10,r11,r12,r13,r14,r15,r16,r17,r18][CH,CH2;r9,r10,r11,r12,r13,r14,r15,r16,r17,r18][O,S,#7;R1;r9,r10,r11,r12,r13,r14,r15,r16,r17,r18][CH,CH2;r9,r10,r11,r12,r13,r14,r15,r16,r17,r18][CH,CH2;r9,r10,r11,r12,r13,r14,r15,r16,r17,r18][O,S,#7;R1;r9,r10,r11,r12,r13,r14,r15,r16,r17,r18]),$([O,S,#7;R1;r9,r10,r11,r12,r13,r14,r15,r16,r17,r18][CH,CH2;r9,r10,r11,r12,r13,r14,r15,r16,r17,r18][CH,CH2;r9,r10,r11,r12,r13,r14,r15,r16,r17,r18][CH,CH2;r9,r10,r11,r12,r13,r14,r15,r16,r17,r18][O,S,#7;R1;r9,r10,r11,r12,r13,r14,r15,r16,r17,r18][CH,CH2;r9,r10,r11,r12,r13,r14,r15,r16,r17,r18][CH,CH2;r9,r10,r11,r12,r13,r14,r15,r16,r17,r18][CH,CH2;r9,r10,r11,r12,r13,r14,r15,r16,r17,r18][O,S,#7;R1;r9,r10,r11,r12,r13,r14,r15,r16,r17,r18]),$([O,S,#7;R1;r9,r10,r11,r12,r13,r14,r15,r16,r17,r18][CH,CH2;r9,r10,r11,r12,r13,r14,r15,r16,r17,r18][CH,CH2;r9,r10,r11,r12,r13,r14,r15,r16,r17,r18][O,S,#7;R1;r9,r10,r11,r12,r13,r14,r15,r16,r17,r18][CH,CH2;r9,r10,r11,r12,r13,r14,r15,r16,r17,r18][CH,CH2;r9,r10,r11,r12,r13,r14,r15,r16,r17,r18][CH,CH2;r9,r10,r11,r12,r13,r14,r15,r16,r17,r18][O,S,#7;R1;r9,r10,r11,r12,r13,r14,r15,r16,r17,r18])]
cyanamide	N[CH2]C#N
cyanidin	[OH]c1cc([OH])cc2=[O+]C(=C([OH])Cc21)c3cc([OH])c([OH])cc3
cyano_phosphonate	P(O[A,a])(O[A,a])(=O)C#N
cyanohydrin	[C;X4](-[OH,NH1,NH2,SH])(-C#N)
cyanophosphonate	P(OCC)(OCC)(=O)C#N
cycloheximide	O=C1CCCC(N1)=O
cytochalasin	O=C1NCC2CCCCC21
di_tri_phosphate	P(=O)([OH])OP(=O)[OH]
diamino_sulfide	[N,n]~[S;!R;D2]~[N,n]
diazo_carbonyl	[$(N=N=C~C=O),$(N#N-C~C=O)]
diazonium	a[N+]#N
dicarbonyl_sulfonamide	[$(N(-C(=O))(-C(=O))(-S(=O))),$(n([#6](=O))([#6](=O))([#16](=O)))]
dihydroxybenzene	[OH1]c1ccc([OH1])cc1
disulfide	SS
disulfide_acyclic	[S;!R;X2]-[S;!R;X2]
disulfonyliminoquinone	S(=O)(=O)N=C1C=CC(=NS(=O)(=O))C=C1
double_trouble_wAr-Head	NC(C[S;D1])C([N;H1]([O;D1]))=O
epoxide_aziridine_thioepoxide	[CH2]1[O,S,N]C1
flavanoid	O=C2CC(a3aaaaa3)Oa1aaaaa12
four_nitriles	C#N.C#N.C#N.C#N
free_thiol	[SH]
halo_5heterocycle_bis_EWG	[#7,#8,#16]1[#6]([$(S(=O)(=O)),$([F,Cl]),$(C(F)(F)(F)),$(C#N),$(N(=O)(=O)),$([N+](=O)[O-]),$(C(=O))])[#6]([$(S(=O)(=O)),$([F,Cl]),$(C(F)(F)(F)),$(C#N),$(N(=O)(=O)),$([N+](=O)[O-]),$(C(=O))])[#7][#6]1([Cl,Br,I])
halo_acrylate	[$([C;H2]),$([C&H1;$(C-F)]),$([C&H1;$(C-Cl)]),$([C&H1;$(C-Br)]),$([C&H1;$(C-I)]),$(C(F)F),$(C(Cl)Cl),$(C(Br)Br),$(C(I)I),$(C(F)Cl),$(C(F)Br),$(C(F)I),$(C(Cl)Br),$(C(Br)I)](=[$([C&H1;$(C(-C(=O)))]),$(C(F)(C(=O))),$(C(Cl)(C(=O))),$(C(Br)(C(=O))),$(C(I)(C(=O))),$(C(C)(C(=O))),$(C(c)(C(=O)))])

Filter	SMARTS
halo_imino	C(=[#7])([Cl,Br,I,$(O(S(=O)(=O)))])
halo_olefin_bis_EWG	C([Cl,Br,I,$(O(S(=O)(=O)))])=C([$(S(=O)(=O)),$(C(F)(F)(F)),$(C#N),$(N(=O)(=O)),$([N+](=O)[O-]),$(C=O)])([$(S(=O)(=O)),$(C(F)(F)(F)),$(C#N),$(N(=O)(=O)),$([N+](=O)[O-]),$(C=O)])
halo_phenolic_carbonyl	C(=O)Oc1c([Cl,F])[cH1,$(c[F,Cl])]c([F,Cl])[cH1,$(c[F,Cl])]c1([F,Cl])
halo_phenolic_sulfonyl	S(=O)Oc1c([Cl,F])[cH1,$(c[F,Cl])]c([F,Cl])[cH1,$(c[F,Cl])]c1([F,Cl])
halogen_heteroatom	[!C;!c;!H][F,Cl,Br,I]
hemiacetal	[#6]-O[CH1](-[#6])[OH1]
hetero_silyl	[Si]~[!#6]
heteroaryl_sulfonate	a-S(=O)(=O)-O-[$([a&!#6]),$(c[a&!#6]),$(cc[a&!#6]),$(ccc[a&!#6]),$(cccc[a&!#6]),$(ccccc[a&!#6])]
HOBT_ester	O=C(-[!N])O[$(nnn),$([#7]-[#7]=[#7])]
hydrazine2	[#7]!@-N!@=C
hydrazine	[N;X3;!$(N-S(=O)(=O));!$(N-C(F)(F)(F));!$(N-C#N);!$(N-C(=O));!$(N-C(=S));!$(N-C(=N))]-[N;X3;!$(N-S(=O)(=O));!$(N-C(F)(F)(F));!$(N-C#N);!$(N-C(=O));!$(N-C(=S));!$(N-C(=N))]
hydrazothiourea	[N;!R]=NC(=S)N
hydroxamate_wAr-Head	C([N;H1]([O;D1]))=O
hyperval_sulfur	[$([#16&D3]),$([#16&D4])]=,:[#6]
Imine1	[#6;R0]C([#6;R0])=[NH1]
Imine2	[#6;R0][CH1]=[NH1]
isonitrile	[N+]#[C-]
Lawesson_reagent_derivatives	P(=S)(S)S
linear_polycyclic_aromatic_I	[$(a12aaaaa1aa3a(aa(aaaa4)a4a3)a2),$(a12aaaaa1aa3a(aaa4a3aaaa4)a2),$(a12aaaaa1a(aa5)a3a(aaa4a3a5aaa4)a2)]
linear_polycyclic_aromatic_II	[$(a12aaaa4a1a3a(aaaa3aa4)aa2),$(a12aaaaa1a3a(aaa4a3aaaa4)aa2),$(a1(a(aaaa4)a4a3a2aaaa3)a2aaaa1)]
maleimide_etc	[$([C;H1]),$(C(-[F,Cl,Br,I]))]1=[$([C;H1]),$(C(-[F,Cl,Br,I]))]C(=O)[N,O,S]C(=O)1
meldrums_acid_etc	O=C1OC(C)(C)OC(C1)=O
metal	[$([Ru]),$([Mg]),$([Rh]),$([Se]),$([se]),$([Pd]),$([Sc]),$([Bi]),$([Sb]),$([Ag]),$([Ti]),$([Al]),$([Cd]),$([V]),$([In]),$([Cr]),$([Sn]),$([Mn]),$([La]),$([Fe]),$([Er]),$([Tm]),$([Yb]),$([Lu]),$([Hf]),$([Ta]),$([W]),$([Re]),$([Co]),$([Os]),$([Ni]),$([Ir]),$([Cu]),$([Zn]),$([Ga]),$([Ge]),$([As]),$([as]),$([Y]),$([Zr]),$([Nb]),$([Ce]),$([Pr]),$([Nd]),$([Sm]),$([Eu]),$([Gd]),$([Tb]),$([Dy]),$([Ho]),$([Pt]),$([Au]),$([Hg]),$([Tl]),$([Pb]),$([Ac]),$([Th]),$([Pa]),$([Mo]),$([U]),$([Tc]),$([Te]),$([Po]),$([At])]
michael_acceptor6	[#6,#7]-&!@[#6](=&!@[CH])-&!@C(=O)-&!@[C,N,O,S]
michael_acceptor5	N#CC(=C)C#N
michael_acceptor_misc	O=C1[O,N]C~[N,C]C1=[C,N]
michael_acceptor_misc2	*~\C=C1/CC2=CC=CC=C2N1
michael_acceptor_vinyl2	[CH2]=C-C(=O)-[#6,#7,#8]
misc_10_carbon_sb_chain	[C;!R]-[C;!R]-[C;!R]-[C;!R]-[C;!R]-[C;!R]-[C;!R]-[C;!R]-[C;!R]-[C;!R]
misc_2_free_phos	P([O;D1])=O.P([O;D1])=O
misc_2_N_quats	[N,n;H0;+;!$(N~O);!$(n~O)].[N,n;H0;+;!$(N~O);!$(n~O)]
misc_2_sulfonic_acid	[C,c]S(=O)(=O)[O;D1].[C,c]S(=O)(=O)[O;D1]
misc_3_COOH	C(=O)[O;D1].C(=O)[O;D1].C(=O)[O;D1]
misc_3_iodine	[#53].[#53].[#53]
misc_4_basic_N	[N;!$(N(=[N,O,S,C]));!$(N(S(=O)(=O)));!$(N(C(F)(F)(F)));!$(N(C#N));!$(N(C(=O)));!$(N(C(=S)));!$(N(C(=N)));!$(N(#C));!$(N-c)].[N;!$(N(=[N,O,S,C]));!$(N(S(=O)(=O)));!$(N(C(F)(F)(F)));!$(N(C#N));!$(N(C(=O)));!$(N(C(=S)));!$(N(C(=N)));!$(N(#C));!$(N-c)].[N;!$(N(=[N,O,S,C]));!$(N(S(=O)(=O)));!$(N(C(F)(F)(F)));!$(N(C#N));!$(N(C(=O)));!$(N(C(=S)));!$(N(C(=N)));!$(N(#C));!$(N-c)].[N;!$(N(=[N,O,S,C]));!$(N(S(=O)(=O)));!$(N(C(F)(F)(F)));!$(N(C#N));!$(N(C(=O)));!$(N(C(=S)));!$(N(C(=N)))

Filter	SMARTS
	;!$(N(#C));!$(N-c)]
misc_4_nitro	[$([N+](=O)[O-]),$(N(=O)=O)].[$([N+](=O)[O-]),$(N(=O)=O)].[$([N+](=O)[O-]),$(N(=O)=O)].[$([N+](=O)[O-]),$(N(=O)=O)]
misc_5_phenolic_OH	a[O;D1].a[O;D1].a[O;D1].a[O;D1].a[O;D1]
misc_7_aliphatic_OH	C[O;D1].C[O;D1].C[O;D1].C[O;D1].C[O;D1].C[O;D1].C[O;D1]
misc_7_total_hal	[Cl,Br,I].[Cl,Br,I].[Cl,Br,I].[Cl,Br,I].[Cl,Br,I].[Cl,Br,I].[Cl,Br,I]
misc_8_CF2_or_CH2	[CH2,$(CF2);R0][CH2,$(CF2);R0][CH2,$(CF2);R0][CH2,$(CF2);R0][CH2,$(CF2);R0][CH2,$(CF2);R0][CH2,$(CF2);R0][CH2,$(CF2);R0]
monensin	O1CCCCC1C2CCCO2
monofluoroacetate	[C;H2](F)C(=O)[O,N,S]
nitrate	[#6]-O-[N+](=O)[O-]
nitro_aromatic	(a-[N+](=O)[O-].a-[N+](=O)[O-])
nitroalkane	C[N+](=O)[O-]
nitrone	[C;!R]=[N+][O;D1]
nitrosamine	N-[N;X2](=O)
nitroso	[N&D2](=O)
NO_phosphonate	P(=O)ON
ortho_hydroiminoquinone	c1c([N;D1])c([N;D1])c[cH1][cH1]1
ortho_hydroquinone	a1c([O,S;D1])c([O,S;D1])a[cH1][cH1]1
ortho_nitrophenyl_carbonyl	[#6]1(-O-[C;!R](=[O,N;!R]))[#6]([$(N(=O)(=O)),$([N+](=O)[O-])])[#6][#6][#6][#6]1
ortho_quinone	[CH1,$(C(-[Cl,Br,I]))]1=CC(=[O,N,S;!R])C(=[O,N,S])C=[CH1,$(C(-[Cl,Br,I]))]1
oxaziridine	C1~[O,S]~N1
oxime	[$(C=N[O;D1]);!$(C=[N+])][#6][#6]
oxonium	[o+,O+]
P_S_halide	[P,S][F,Cl,Br,I]
para_hydroiminoquinone	a1[cH1]c([N;D1])[cH1]ac([N;D1])1
para_hydroquinone	a1[cH1]c([O,S;D1])[cH1]ac([O,S;D1])1
para_nitrophenyl_ester	[#6]1(-O(-[C;!R](-[!N])(=[O,N;!R])))[#6][#6][#6]([$(N(=O)(=O)),$([N+](=O)[O-])])[#6][#6]1
para_quinone	[CH1,$(C(-[Cl,Br,I]))]1=[CH1,$(C(-[Cl,Br,I]))]C(=[O,N,S])[CH1,$(C(-[Cl,Br,I]))]=[CH1,$(C(-[Cl,Br,I]))]C1(=[O,N,S])
paraquat_like	[#6]1[#6][#6]([#6]2[#6][#6][#7;+][#6][#6]2)[#6][#6][#7;+]1
pentafluorophenylester	C(=O)Oc1c(F)c(F)c(F)c(F)c1(F)
perchloro_cp	C1(Cl)(Cl)C(Cl)C(Cl)=C(Cl)C1(Cl)
perhalo_dicarbonyl_phenyl	c1(C=O)c([Br,Cl,I])c([Br,Cl,I])c([Br,Cl,I])c([Br,Cl,I])c1(C=O)
perhalo_ketone	O=CC(-[F,Cl,Br,I])(-[F,Cl,Br,I])-[F,Cl,Br,I]
perhalo_phenyl	c1c([F,Br,Cl,I])c([F,Br,Cl,I])c([F,Br,Cl,I])c([F,Br,Cl,I])c1([F,Br,Cl,I])
peroxide	[#8]~[#8]
phenolate_bis_EWG	O=[C,S]Oc1aaa([$(S(=O)(=O)),$(C(F)(F)(F)),$(C#N),$(N(=O)(=O)),$([N+](=O)[O-]),$(C(=O)O),$(C(=O)N)])aa([$(S(=O)(=O)),$(C(F)(F)(F)),$(C#N),$(N(=O)(=O)),$([N+](=O)[O-]),$(C(=O)O),$(C(=O)N)])1
phos_serine_wAr-Head	NC(COP(O)(O)=O)C(O)=O
phos_threonine_wAr-Head	NC(C(C)OP(O)(O)=O)C(O)=O
phos_tyrosine_wAr-Head	NC(Cc1ccc(OP(O)(O)=O)cc1)C(O)=O
phosphite	[c,C]-[P;v3]
phosphonate_esters	COP(=O)(=O)[C,c]
phosphonium	[#15;+]~[!O]
phosphoramide	NP(=O)(N)N
phosphorane	C=P

Filter	SMARTS
phosphorous_nitrogen_bond	[#15]~[N,n]
phosphorus_phosphorus_bond	P~P
phosphorus_sulfur_bond	P~S
polyacidic4	[C,S,P](=O)[OH].[C,S,P](=O)[OH].[C,S,P](=O)[OH].[C,S,P](=O)[OH]
polyazoanthracene	c12:[c,n]:[c,n]:[c,n]:[c,n]:c1[c,n]c3:[c,n]:[c,n]:[c,n]:[c,n]:c3[c,n]2
polyazophenanthrene	c12:[c,n]:[c,n]:[c,n]:[c,n]:c1:[c,n]:[c,n]:c3:[c,n]:[c,n]:[c,n]:[c,n]:c23
polyene	C=[C;!R][C;!R]=[C;!R][C;!R]=[C;!R]
polyhalo_phenol_a	c1c([O;D1])c(-[Cl,Br,I])c(-[Cl,Br,I])cc1.c1c([O;D1])c(-[Cl,Br,I])c(-[Cl,Br,I])cc1
polyhalo_phenol_b	c1c([O;D1])c(-[Cl,Br,I])cc(-[Cl,Br,I])c1.c1c([O;D1])c(-[Cl,Br,I])cc(-[Cl,Br,I])c1
polyhalo_phenol_c	c1c([O;D1])ccc(-[Cl,Br,I])c(-[Cl,Br,I])1.c1c([O;D1])ccc(-[Cl,Br,I])c(-[Cl,Br,I])1
polyhalo_phenol_d	c(-[Cl,Br,I])1c([O;D1])c(-[Cl,Br,I])ccc1.c(-[Cl,Br,I])1c([O;D1])c(-[Cl,Br,I])ccc1
polyhalo_phenol_e	c1c([O;D1])ccc(-[Cl,Br,I])c(-[Cl,Br,I])1.c1c([O;D1])ccc(-[Cl,Br,I])c(-[Cl,Br,I])1
polysulfide	[S;D2]-[S;D2]-[S;D2]
porphyrin	[#6;r16,r17,r18]~[#6]1~[#6]~[#6]~[#6](~[#6])~[#7]1
primary_halide_sulfate	[CH2][Cl,Br,I,$(O(S(=O)(=O)[!$(N);!$([O&D1])]))]
propiolactone	C1(=O)OCC1
quat_N_acyl	[N,n;+]!@C(=O)
quat_N_N	[N,n;R;+]!@[N,n]
quaternary_C_Cl_I_P_S	[C+,Cl+,I+,P+,S+]
quaternary_nitroxy	C[N+](-[O-])(C)C
quinone_methide	[#6;!$([#6](-[N,O,S]))]1=[#6;!$([#6](-[N,O,S]))][#6](=[#6])[#6;!$([#6](-[N,O,S]))]=[#6;!$([#6](-[N,O,S]))][#6]1(=[O,N,S])
rhodanine	C(=C)1SC(=S)NC(=O)1
secondary_halide_sulfate	[CH;!$(C=C)][Cl,Br,I,$(O(S(=O)(=O)[!$(N);!$([O&D1])]))]
squalestatin	C12OCCC(O1)CC2
sulf_D2_nitrogen	[S;D2](-[N;!$(N(=C));!$(N(-S(=O)(=O)));!$(N(-C(=O)))])
sulf_D2_oxygen_D2	[S;D2][O;D2]
sulf_D3_nitrogen	[S;D3](-N)(-[c,C])(-[c,C])
sulfite_sulfate_ester	[C,c]OS(=O)O[C,c]
sulfonate	COS(=O)(=O)[C,c]
sulfonium	[S+;X3;$(S-C);!$(S-[O;D1])]
sulfonyl_anhydride	[$(C(=O)),$(S(=O)(=O))][O,S](S(=O)(=O))
sulfonyl_halide	S(=O)(=O)[F,Cl,Br,I]
sulfonyl_heteroatom	[!#6;!#1;!#11;!#19]O(S(=O)(=O)(-[C,c]))
sulphonyl_cyanide	S(=O)(=O)C#N
tertiary_halide_sulfate	[C;X4](-[Cl,Br,I,$(O(S(=O)(=O)[!$(N);!$([O&D1])]))])(-[c,C])(-[c,C])(-[c,C])
thio_hydroxamate	[S;D2]([$(N(=C)),$(N(-S(=O)(=O))),$(N(-C(=O)))])
thio_xanthate	[S;!R]-[C;!R](=[S;!R])(-[S;!R])
thioamide	[#6]C([#7H2])=S
thiocarbonate	SC(=O)[O,S]
thiocyanate	SC#N
thioester	[S;!R;H0]C(=[S,O;!R])([!O;!S;!N])
thioketone	CC(=S)C
thiol_wAr-Head	NC(C[S;D1])C(O)=O
thiopyrylium	c1[S,s;+]cccc1
thiosulfoxide	[C,c][S;X3](~O)-S
thiourea	C([#7H2])([#7H2])=S
tri_phosphoric_esters	([#6]OP(=O)(-*)O[#6].[#6]OP(=O)(-*)O[#6].[#6]OP(=O)(-*)O[#6])

Filter	SMARTS
triacyloxime	C(=O)N(C(=O))OC(=O)
triamide	[$(N(-C(=O))(-C(=O))(-C(=O))),$(n([#6](=O))([#6](=O))([#6](=O)))]
triaryl_phosphine_oxide	P(=O)(a)(a)(a)
trichloromethyl_ketone	[$(C(=O));!$(C-N);!$(C-O);!$(C-S)]C(Cl)(Cl)(Cl)
triflate	OS(=O)(=O)(C(F)(F)(F))
trifluoroacetate_ester	C(F)(F)(F)C(=O)O
trifluoroacetate_thioester	C(F)(F)(F)C(=O)S
trifluoromethyl_ketone	[$(C(=O));!$(C-N);!$(C-O);!$(C-S)]C(F)(F)(F)
trihalovinyl_heteroatom	C(-[Cl,Br,I])(-[Cl,Br,I])=C(-[Cl,Br,I])(-[N,O,S])
trinitro_aromatic	[$(a1aaa([$(N(=O)(=O)),$([N+](=O)[O-])])a([$(N(=O)(=O)),$([N+](=O)[O-])])a1([$(N(=O)(=O)),$([N+](=O)[O-])])),$(a1aa([$(N(=O)(=O)),$([N+](=O)[O-])])a([$(N(=O)(=O)),$([N+](=O)[O-])])aa1([$(N(=O)(=O)),$([N+](=O)[O-])])),$(a1a([$(N(=O)(=O)),$([N+](=O)[O-])])aa([$(N(=O)(=O)),$([N+](=O)[O-])])aa1([$(N(=O)(=O)),$([N+](=O)[O-])]))]
trinitromethane_derivative	C([$([N+](=O)[O-]),$(N(=O)=O)])([$([N+](=O)[O-]),$(N(=O)=O)])([$([N+](=O)[O-]),$(N(=O)=O)])
tris_activated_aryl_ester	[$(O=[C,S]Oc1a([$(S(=O)(=O)),F,$(C(F)(F)(F)),$(C#N),$(N(=O)(=O)),$([N+](=O)[O-]),$(C(=O)O),$(C(=O)N)])a([$(S(=O)(=O)),F,$(C(F)(F)(F)),$(C#N),$(N(=O)(=O)),$([N+](=O)[O-]),$(C(=O)O),$(C(=O)N)])a([$(S(=O)(=O)),F,$(C(F)(F)(F)),$(C#N),$(N(=O)(=O)),$([N+](=O)[O-]),$(C(=O)O),$(C(=O)N)])aa1),$(O=[C,S]Oc1a([$(S(=O)(=O)),F,$(C(F)(F)(F)),$(C#N),$(N(=O)(=O)),$([N+](=O)[O-]),$(C(=O)O),$(C(=O)N)])a([$(S(=O)(=O)),F,$(C(F)(F)(F)),$(C#N),$(N(=O)(=O)),$([N+](=O)[O-]),$(C(=O)O),$(C(=O)N)])aaa([$(S(=O)(=O)),F,$(C(F)(F)(F)),$(C#N),$(N(=O)(=O)),$([N+](=O)[O-]),$(C(=O)O),$(C(=O)N)])1),$(O=[C,S]Oc1a([$(S(=O)(=O)),F,$(C(F)(F)(F)),$(C#N),$(N(=O)(=O)),$([N+](=O)[O-]),$(C(=O)O),$(C(=O)N)])aa([$(S(=O)(=O)),F,$(C(F)(F)(F)),$(C#N),$(N(=O)(=O)),$([N+](=O)[O-]),$(C(=O)O),$(C(=O)N)])a([$(S(=O)(=O)),F,$(C(F)(F)(F)),$(C#N),$(N(=O)(=O)),$([N+](=O)[O-]),$(C(=O)O),$(C(=O)N)])a1),$(O=[C,S]Oc1a([$(S(=O)(=O)),F,$(C(F)(F)(F)),$(C#N),$(N(=O)(=O)),$([N+](=O)[O-]),$(C(=O)O),$(C(=O)N)])aa([$(S(=O)(=O)),F,$(C(F)(F)(F)),$(C#N),$(N(=O)(=O)),$([N+](=O)[O-]),$(C(=O)O),$(C(=O)N)])aa([$(S(=O)(=O)),F,$(C(F)(F)(F)),$(C#N),$(N(=O)(=O)),$([N+](=O)[O-]),$(C(=O)O),$(C(=O)N)])1)]
trisub_bis_act_olefin	[[CH;!R];!$(C-N)]=C([$(S(=O)(=O)),$(C(F)(F)(F)),$(C#N),$(N(=O)(=O)),$([N+](=O)[O-]),$(C(=O))])([$(S(=O)(=O)),$(C(F)(F)(F)),$(C#N),$(N(=O)(=O)),$([N+](=O)[O-]),$(C(=O))])
unacceptable_atoms1	[!#6;!#7;!#8;!#16;!#1;!#3;!#9;!#11;!#12;!#15;!#17;!#19;!#20;!#30;!#35]
unacceptable_atoms2	[!#6;!#7;!#8;!#16;!#1;!#3;!#9;!#11;!#12;!#15;!#17;!#19;!#20;!#30;!#35;!#53]
vinyl_carbonyl_EWG	[C;!R]([$(S(=O)(=O)),$(C(F)(F)(F)),$(C#N),$(N(=O)(=O)),$([N+](=O)[O-]),$(C=O)])([$(S(=O)(=O)),$(C(F)(F)(F)),$(C#N),$(N(=O)(=O)),$([N+](=O)[O-]),$(C=O)])=[C;!R]([C;!R](=O))([!$([#8]);!$([#7])])
vinyl_sulfone	O=S([#6]=[#6])([#6]=[#6])=O
vinyloxazole	[N,C]=CC1=COC=N1
2,3,4-trihydroxyphenyl	c([OH])c([OH])c([OH])

Table S8. Undesirable functionality SMARTS definitions utilised by the NIH.[3]

Filter	SMARTS
thiocarbonyl	[c,C]=[S;X1]
termalkyne	[CH]#C
quinonepara	O=[#6]1[#6]~[#6][#6](=O)[#6]~[#6]1
nonpeptidic_macrocycle	[!R0!r3!r4!r5!r6!r7!r8!$([N;!H0,$(N1[CH2][CH2][CH2][CH1]1)][CH]C=O)!$([CH]([N;!H0,$(N1[CH2][CH2][CH2][CH1]1)])C=O)!$(C(=O)[CH][N;!H0,$(N1[CH2][CH2][CH2][CH1]1)])]
nitrogen_oxygen_bond	*-[n,N]-[O;H0;R0]
methyl_ester_x2	[$([CH3]OC=O)].[$([CH3]OC=O)]
imide	O=C([#6])NC(=O)[#6]
exocyclic_double_bond_toC	[R;#7,#8,#16,#6X3][R]=!@C
ethyl_ester_x2	[$([CH2](OC=O)[CH3])][CH3].[$([CH2](OC=O)[CH3])][CH3]
ester_deep_in_mol	*[#6]C(=O)[O;R0][#6;$(*(OC=O)**),$(*(OC=O)(*)*)]
enolether	C=!@C[OD2]
conjugated_C=C	C=[C;R0][C;R0]=C
benzyl_ester	[$([CH2](OC=O)c1[cH][cH][cH][cH][cH]1)]c1[cH][cH][cH][cH][cH]1
aromatic_tricyclic1	c1ccc3c(c1)[C;!$(C=O)]c2ccccc23
allyl_ester	[$([CH2](OC=O)[CH]=[CH2])][CH]=[CH2]
alkylNandNonC	N[CX4]!@N
alkCl	[C][Cl!$(ClC(Cl)(Cl))]
alkBr	CBr
acyclic_sulphur_michael_acceptor	[C!$(*[Nv3X3])]=!@[C!$(*[Nv3X3])][S!$(*[Nv3X3])]=O
acyclic_imine	[C!$(*(=N)[N,n])]=!@[Nv3!$(*O)]
acyclic_hydrazine	[Nv3X3!$(*(C=O)NC=O)]-!@[Nv3X3!$(*(C=O)NC=O)]
acetyl_x2	[CH3]C(=O)O.[CH3]C(=O)O
acetal	[OX2;$(OC[OX2])][C;!$(C1(O)CNCCO1);!$(C1(O)(CO)OC(CO)C(O)C1O);!$(C1(O)OC(CO)C(O)C(O)C1O)][OX2][!a]
OCO_protecting_group	[O;R0][C;X4][O;R0]
N-SO_group	N[S;!$(S(=O)(=O))]=O
C=N=O_gp	C=N=O
C(=O)CC(=O)_gp	[c,C]C(=O)[C!H0!R]C(=O)[C,c]
4_fused_ring_sys	[R2][R3][R2][R2][R2]
C#C	C#C.C#C
C#C-c_gp	cC#[C!H1]
3_mem_ring_with_het	[S,O,N;r3]
acylcarbamate	O=[S,C]NC(=O)O
anyNO	[Nv3,n]=O
phenol_x2	[OH][c;$(c1ccccc1)].[OH][c;$(c1ccccc1)]
formamide	[#7;!$(N[OH])][CH1]=O
benzyl_halide	[CX4](a)[F,Cl,Br,I;!$(FC(F)F)]

Table S9. Undesirable functionality SMARTS definitions that comprise the 'GSKB' filter.[4]

Filter	
Acyl halide	Disulfide
Aldehyde	Hydrazine (terminal)
Alkyl halide	Isocyanate
Anhydride	Isothiocyanate
Diazo	Peroxide
Dicarbonyl	Quaternary ammonium

Table S10. Undesirable functionality filters used in the ‘HTS Filter’ embedded in Pipeline Pilot.

3.0 References

[1] A. S. Deeming, C. J. Russell, M. C. Willis, *Angew. Chem. Int. Ed. Engl.* **2015**, *54*, 1168–71.

[2] N. C. Firth, N. Brown, J. Blagg, *J. Chem. Inf. Model.* **2012**, *52*, 2516–25.

[3] A. Jadhav, R. S. Ferreira, C. Klumpp, B. T. Mott, C. P. Austin, J. Inglese, C. J. Thomas, D. J. Maloney, B. K. Shoichet, A. Simeonov, *J. Med. Chem.* **2010**, *53*, 37–51.

[4] A. Nadin, C. Hattotuwagama, I. Churcher, *Angew. Chem. Int. Ed.* **2012**, *51*, 1114–1122.
